# The Roles of Plasticity and Selection in Rapid Phenotypic Changes at the Pacific Oyster Invasion Front in Europe

**DOI:** 10.1111/mec.17684

**Published:** 2025-02-07

**Authors:** Alexandra Kinnby, Chloé Robert, Jonathan N. Havenhand, Göran Broström, Luc Bussière, Pierre De Wit

**Affiliations:** ^1^ Department of Marine Sciences University of Gothenburg Göteborg Sweden; ^2^ Nofima AS Tromsø Norway; ^3^ Centre for Marine Evolutionary Biology (CeMEB) Göteborg Sweden; ^4^ Department of Biological and Environmental Sciences University of Gothenburg Göteborg Sweden; ^5^ Gothenburg Global Biodiversity Centre (GGBC) Göteborg Sweden

**Keywords:** *Crassostrea (Magallana) gigas*, fertilisation, invasion biology, low‐salinity adaptation

## Abstract

Invasive species present significant management challenges worldwide due to their ability to rapidly adapt to novel environments. The Pacific oyster *Crassostrea gigas* , a globally distributed invasive species, arrived in western Sweden in 2006 but has not yet colonised the low salinity waters of the Baltic Sea, presumably because low salinities act as a barrier to reproduction. We used classic mating designs to investigate fertilisation rates and heritability of embryonal salinity tolerance (in 8‰–33‰) in oysters from three locations with different invasion history and salinity (*established*, 33‰; *past* invasion front, 23.5‰; and *present* invasion front, 16‰). We found that fertilisation rates at lower salinities increased with proximity to the range front, with a pronounced heritable component. We then used whole‐genome sequencing of oysters from the *present* invasion front to identify genomic regions showing stronger deviations from Mendelian inheritance in larval full‐sib families reared in low salinity compared to controls. These regions contained coding sequences for Histones and ribosomal DNA, with the paternal genotype explaining a significant proportion of the deviation, suggesting the involvement of sperm in modulation of low‐salinity tolerance at fertilisation and early development. Furthermore, we found no evidence of recent bottlenecks along the invasion front. We conclude that the Pacific oyster has developed low‐salinity tolerant reproductive phenotypes at the *present* invasion front through acclimation and natural selection. Given the strong heritability for tolerance to low‐salinities at fertilisation, the species likely has the potential to adapt further to low‐salinity conditions and may invade the Baltic Sea.

## 1 Introduction

Global biodiversity is acutely threatened. Current extinction rates—between 1000 and 10,000 times faster than the natural background rate (Pimm et al. 2014)—are resulting in the loss of key ecosystem services such as food provisioning, erosion protection, and carbon management (Worm et al. 2006). Effective management of declining biodiversity has proven problematic but is fundamental to maintaining a sustainable relationship with the natural environment. Doing this will require well‐informed, bespoke management strategies which address the root causes of the crisis (Roberts 2010). One of the most important of these causes is the impact of invasive species, which may be introduced through natural as well as anthropogenic processes (e.g., Lowry et al. 2013; Milardi et al. 2022).

Invasive species typically enter novel ecosystems through expansions at range edges (Legault et al. 2020) or anthropogenic vectors (Ricciardi et al. 2021), which can have negative impacts on native species and the environment (Allendorf and Lundquist 2003). These expansions are often linked to climate change (Legault et al. 2020), and as invasive species by definition have few or no natural predators in their new ecosystem, they frequently outcompete native species, and often cause local extinctions (biological invasions are one of the global change drivers most strongly associated with species extinctions; Vitousek et al. 1997; Crystal‐Ornelas and Lockwood 2020). Most invasive species share a suite of phenotypic traits such as high reproductive potential, high adaptability or plasticity, broad environmental tolerance, and efficient dispersal, which together allow them to rapidly colonise, establish, and build self‐sustaining populations from which they can spread further (Kolar and Lodge 2001). High adaptive plasticity (the ability of an organism to express different phenotypes in response to varying stimuli or inputs from the environment without genetic change) can provide a powerful advantage to invasive species (Davidson et al. 2011). Adaptive plasticity interacts with evolutionary change by bringing the mean phenotype closer to the fitness optimum, thereby relaxing the selective pressure on the population. While this lower pressure could slow down the process of adaptation to the novel environment, it also allows for maintenance of a greater population size, which can avoid bottleneck effects or accumulation of deleterious alleles due to stochastic events (Allendorf and Lundquist 2003; Chevin and Lande 2015). This can be especially important at the invasion front if the propagule pressure is high enough. However, phenotypic plasticity can also lead to trade‐offs that limit the benefits of plasticity. For example, optimization for marginal environments can increase susceptibility to predators or grazers—the brown alga *Fucus vesiculosus* exhibits a plastic response to increased *p*CO_2_ by growing faster, but in doing so it also becomes more susceptible to grazing (Kinnby et al. 2021). Limitations also arise as a result of reductions in genetic diversity due to bottlenecks at invasion fronts. If there is no potential for local adaptation due to insufficient genetic diversity at the invasion front, or if maladaptation occurs due to accumulation of deleterious alleles, the invasion will stagnate (Kirkpatrick and Barton 1997; García‐Ramos and Rodríguez 2002).

During an invasion, individuals at the advancing edge of a population are exposed to novel biotic and abiotic variables, which can lead to changes in the genetic makeup of the population at the front of the expansion. At the range front, population sizes are generally low, leading to bottlenecks. Bottlenecks can reduce a species' ability to adapt by reducing genetic diversity (Spielman et al. 2004). However, they can also allow for rapid changes in genetically encoded phenotypic traits along the expansion front, as seen in e.g., green crabs on the east coast of North America (Tepolt and Palumbi 2020). Range front populations often experience different selection pressures to those in the native range, which favour certain alleles or genetic variants to become more common (Excoffier et al. 2009; although stochastic processes associated with the bottleneck might interfere with local adaptation, Eckert et al. 2008). For example, allelic surfing or hitchhiking can occur: processes by which genetic drift can drive up the frequencies of neutral or deleterious alleles and prevent them from being purged by selection, eventually causing fitness decreases along the expansion front and stopping the range expansion (Excoffier and Ray 2008; Gagnaire et al. 2015). The balance among these genetic processes is important in determining how likely a species is to expand and colonise new geographic areas (Tsutsui et al. 2000; Sakai et al. 2001; Lee 2002).

**FIGURE 1:**
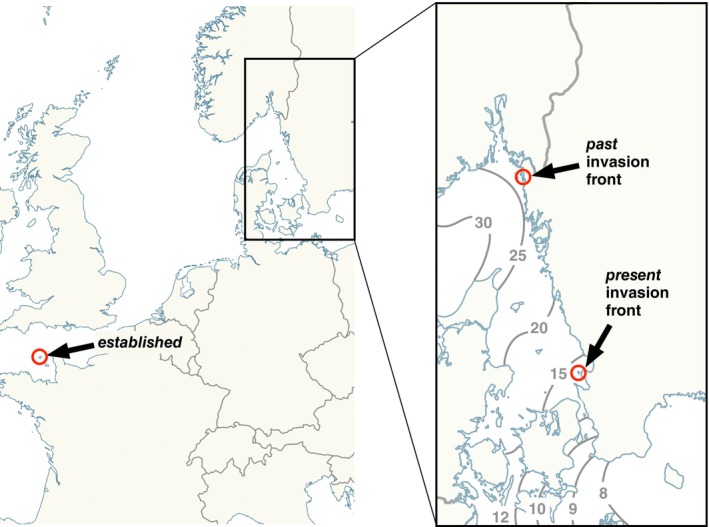
Map of the study area, marking the sites used for oyster collection (modified from Wikimedia Commons). Average SSS (Sea Surface Salinity) isohalines (inset) taken from Svensson et al. (2014). “*established”* site = Guernsey, Channel Is.; “*past* invasion front” = Svallhagen, Tjärnö, Sweden; *“present* invasion front” = Hallands Väderö, Sweden (see Table 1 for more details).

One of the most common marine invasive species worldwide is the Pacific oyster, *Crassostrea (Magallana) gigas* (Thunberg, 1789). Native to the western Pacific Ocean, it was introduced to Europe in the mid‐20th century for aquaculture due to its high growth and reproductive rate, and is now established across almost all European coasts (except for the Baltic Sea; Diederich et al. 2005; Cardoso et al. 2007; Wrange et al. 2010). Attempts to establish a Pacific oyster aquaculture industry on the Swedish west coast in the 1970s were unsuccessful, and it was first in 2007, that the species was reported as invasive (Faust et al. 2017; Wrange et al. 2010). Populations of Pacific oysters established rapidly on the northern west coast of Sweden (Wrange et al. 2010), but sexually mature oysters from the central west coast were scarce even in 2015 (Havenhand & Kinnby, unpubl.). Today, the Pacific oyster is well established in shallow waters along the Swedish marine (western) coastline and is expanding southward towards areas of lower salinity (< 15‰; Figure 1). Although the prevalent surface current in this region is northwards, episodic weather‐driven, southward flows, may facilitate this spread. The effects of reduced salinity on marine invertebrate reproduction vary, but often include reduced fertilisation success due to decreased sperm viability, changes to sperm motility, decreased egg quality, and/or increased polyspermy (Allen and Marshall 2014). Prior investigations of fertilisation success of invasive Pacific oysters in Sweden 10 years ago indicated that oysters from the northern west coast were better able to reproduce in lower salinities than oysters from an established population in the British Channel Islands (Kinnby 2015; Havenhand and Kinnby unpubl.). How much further populations on the Swedish west coast have now adapted to lower salinities, and the genomic nature of those adaptations is however unknown. Salinity gradients close to the current invasion front are very sharp (Figure 1), yet beyond this immediate region, much of the southern Baltic has a salinity of ~8‰. The possibility that further adaptation of Pacific oysters might facilitate invasion into these low‐salinity Baltic waters warrants urgent investigation.

Here, we tested the hypothesis that Pacific oysters along an invasion front with a strong environmental gradient have the potential to adapt rapidly to new salinity conditions. In order to separate the effects of plasticity and natural selection, we used classic breeding designs to determine the relative contributions of genetics and plasticity to salinity tolerance at fertilisation in oysters sampled at three different localities along an invasion front from fully marine into brackish water (Figure 1). The three sampling locations represented: (1) fully marine waters (ca. 35‰) in the region where Pacific oysters were first introduced in Europe (from hereon called “*established*” site); (2) waters of ca. 23‰ (range 12‰–34‰) at the past invasion front in Sweden (Wrange et al. 2010; from hereon called “*past* invasion front”); (3) waters of ca. 16‰ (range 9‰–20‰) at the present‐day invasion front in Sweden (from hereon called “*present* invasion front”). We compared fertilisation success among oysters from the different locations, using multiple diallel crosses (North Carolina II design) to determine phenotypic responses, and to estimate the narrow‐sense heritability (*h* ^2^) of fertilisation success at five different salinities in each of the different locations. We then used whole‐genome sequencing, focusing only on oysters from the *present* invasion front, to scan for genomic regions associated with embryonic survival in low salinity (compared to a reference salinity), using deviations from Mendelian inheritance patterns to separate the salinity effect from family‐specific mortalities (genetic load). To our knowledge, this is the first study to separate genetic and plastic effects on oyster fertilisation in low salinities and to examine the genomic mechanism behind low‐salinity adaptation. Our results have important implications for the management of marine invasive species.

## 2 Methods

### 2.1 Oyster Culture

Sexually mature Pacific oysters ( *C. gigas* ) were obtained from three different locations (Figure 1): 1, a site where oysters have been *established* for over 60 years in the English Channel; 2, from the *past* invasion front (oysters present since 2006) on the northern west coast of Sweden (close to the Tjärnö Marine Laboratory, TML); 3, from the *present* invasion front in southwestern Sweden (Hallands Väderö, colonised sometime after 2014). Oysters from the *established* site were provided by Guernsey Sea Farms, Vale, Guernsey, in April–June 2015. Oysters from the *past* invasion front were collected in April–June in both 2015 and 2022 in order to investigate temporal changes in tolerance. Oysters from the *present* invasion front were collected in April, 2022. All oysters were held at TML (58°52′36.4″ N 11°6′42.84″ E) where they were kept in seawater at the same salinity as their origin at the time of collection: 33‰ for the *established* site, 23.5‰ for the *past* invasion front, and 16‰ for the *present* invasion front. Oysters were fed daily with microalgae (*Skeletonema marinoi, Rhodomonas salina*, and *Chaetoceros* spp.).

### 2.2 Seawater Treatments

Experiments were conducted with 0.22 μm Millipore‐filtered deep seawater (FSW; salinity = 33‰) at 20°C ± 0.6°C, which was diluted as required. Five salinity treatments (FSW_*t*_) were examined for each location, chosen to reflect the salinity at each location at the time of collection, and the variance in salinity for that location (Table 1). In order to maintain comparability among sites, we chose treatment values with steps of 5‰. For the *established* and the *past* invasion front, test salinities were 33‰, 28‰, 23‰, 18‰, and 13‰, for the *present* invasion front, the test salinities were 28‰, 23‰, 18‰, 13‰, and 8‰ (NB although the mean salinity at the *established* site was 35.3‰ it was not possible to obtain this salinity at TML without addition of artificial sea salt. Rather than do this, and risk changing the quality of the treatment water we chose instead to use FSW from the TML seawater supply; 33.2‰, 28.1‰–34.9‰, mean, min–max respectively). Reduced salinity levels were achieved by diluting FSW with filtered fresh tap water (FFW) based on observations that tap water more closely reflects the alkalinity of brackish seawater in the region than distilled water. Salinity levels were verified with a conductivity meter (Cond 3210, WTW) calibrated with a 35‰ salinity standard (Hanna Instruments).

**TABLE 1:** Location and salinity characteristics of sampling sites and experimental treatments (SSS = “Sea Surface Salinity”)^a^.

Population	Sampling year	Location	Grid reference	Environmental salinity (‰) mean (min – max)	Experimental salinities (FSW_*t*_, ‰) (“ambient” acclimatisation salinity in bold)
*“established”*	2015	Guernsey, Channel Is.	49°29′49.3″ N, 2°30′8.4″ W	**35.3** (34.8–35.6) ^1^	**33**, 28, 23, 18, 13
*“past* **invasion front** *”*	2015, 2022	Svallhagen, Tjärnö, Sweden	58°52′7.4″ N, 11°9′17.3″ E	**23.5** (11.9–34.5) ^2^	33, 28, **23**, 18, 13
*“present* **invasion front** *”*	2022	Hallands Väderö, Sweden	56°26′39.8″ N, 12°34′9.8″ E	**15.9** (9.7–20.2) ^3^	28, 23, **18**, 13, 8

^a^ 1: SSS data from Copernicus (1993–2023; https://doi.org/10.48670/moi‐00059). 2: SSS data from Tjärnö Marine Laboratory (2014–2017; https://www.weather.loven.gu.se/tjarno/weather‐graph.shtml?tag=Salinity5m). 3: SSS data from in situ EXO‐6P Sonde (April–June 2022; 3.5 m depth).

### 2.3 Gametes and Fertilisation

Gametes were extracted from the gonads either by drilling a hole through the upper shell and inserting a Pasteur pipette (Havenhand and Schlegel 2009) or strip‐spawning (Beaumont et al. 2010, 76). Immediately after extraction, sperm were placed on ice in Eppendorf tubes (one per male) to maximise longevity (Havenhand and Schlegel 2009), and sperm concentration was determined by haemocytometer with a subsample of sperm immobilised in Lugol's solution. Eggs were stored in 10 mL FSW at their respective ambient salinities in an incubator at 20°C. Egg concentrations were determined from repeat counts of 0.1 mL aliquots of egg suspension using an Olympus SZX16 microscope with SDF PLAPO 0.5XPF objective at 0.7x magnification. Eggs were left in their ambient salinities for an hour (Song et al. 2009). This permits the eggs not only to hydrate and “round up”, mimicking natural maturation (Arakawa 1990), but also makes them less vulnerable to polyspermy (Stephano and Gould 1988). Following this hour FFW or FSW was added stepwise, to avoid osmotic shock, during a further hour to yield treatment salinities.

To reduce the risk of polyspermy at fertilisation, sperm was added in 3 increments, 2 min apart (Allen and Marshall 2014). The final sperm concentration was optimised to give no more than a 50% fertilisation rate in the salinity treatment corresponding to the time/location of collection. This was done in order to maximise the sensitivity of the assay and allow for increases as well as decreases in fertilisation success at different salinities (Marshall 2006; Havenhand and Schlegel 2009). After 12 min gametes were separated through centrifugation (5 min at 2000rcf, 20°C; Centrifuge 5819 R, Eppendorf), thus stopping fertilisation (Havenhand and Schlegel 2009). The supernatant containing sperm was discarded and the pellet of fertilised eggs was resuspended in 6.5 mL FSW_*t*_, divided among three 25 mm Petri dishes (to provide partial technical replication), and placed in an incubator (20°C) to develop.

For each population we created multiple blocks of diallel crosses of 3 Males × 3 Females following a classic animal breeding design (North Carolina II; Lynch and Walsh 1998). Each block used gametes from 6 new adults (total of 9 crosses [3 M × 3F] fertilised in 5 salinities with 3 technical replicates per cross & salinity = 135 experimental units per block). In 2015, six blocks were obtained from each of the *established* site and the *past* invasion front, for a total of 18 males and 18 females used per population. In 2022, we obtained 5 experimental blocks per site with oysters from the *past* and *present* invasion front, for a total of 15 males and 15 females used per site.

### 2.4 Fertilisation Success

Two hours after fertilisation, each Petri dish was photographed (Nikon D810 digital SLR; Olympus SZX16 microscope with SDF PLAPO 0.5XPF objective at 5x magnification). Fertilisation success was analysed from the digital images (43 megapixels) in Adobe Photoshop 22.3.0 by counting the number of embryos displaying cell division (*n* = 100 embryos counted per Petri dish). Fertilisation success in the different treatments was then visualised as boxplots in R. In addition, mean fertilisation rates and 95% binomial confidence intervals for each sire (i.e., half‐sibling families) were plotted for each salinity treatment using *ggplot* in R.

### 2.5 Quantitative Genetics

To estimate quantitative genetic parameters, we fit a generalised linear mixed model using Bayesian methods within the *brms* package (Bürkner 2017) in R. The response variable, fertilisation success (%), was transformed into binary data (100 data points per Petri dish) using the *fullfact* package (Houde and Pitcher 2016). We analysed the data using the model:

$$\text{response}\sim \mathrm{sal}+\left(\mathrm{sal}-1\;|\;p\;|\;\mathrm{gr}\left(\text{Sire}\right)\right)+\left(1\;|\;\mathrm{Dam}\right)$$

Where ‘sal’ represents the fixed effect of salinity, ‘(sal− 1 | *p* | gr(Sire))’ is the random effect of sire in the animal model (*‘p’* indicates varying slopes, ‘gr’ indicates grouped for each level of Sire), and ‘(1 | Dam)’ is the random effect of dam (i.e., maternal effects). The sire effect was allowed to vary by treatment with broad *t*‐distribution priors (except for the genetic correlations for which Lewandowski Kurowicka Joe priors were used; Lewandowski et al. 2009), and the model was tasked with estimating correlations across treatments to reconstruct the G‐matrix. Dam effects were not allowed to similarly vary by treatment as genomic results showed that the sperm ribosome and nucleosome are the primary components determining fertilisation success (see Discussion). To ensure sufficient warm‐up and chain mixing, the models were run for 2000 iterations, with the first 1000 iterations designated as warm‐up, after ensuring convergence by visual examination. Samples from the posterior distributions were then used to estimate heritabilities for each population and salinity level, and to examine uncertainty in the genetic correlations. Heritabilities and genetic correlations were illustrated with ridge plots using the *ggridges* package in R. All R code used for the quantitative genetic analyses can be found at: https://github.com/DeWitP/gigas_lowsal_adaptation.

### 2.6 Genomic Data Analysis

The 45 full‐sib families of oyster embryos from the crosses of oysters from the *present* invasion front in 13‰ (from here on “low salinity” treatment) and 23‰ (from here on “reference” treatment) were allowed to develop until D‐stage larvae (24 h post‐fertilisation), after which they were fixed in 95% ethanol. Mantle tissue samples from the 30 parental oysters (15 male and 15 female) were also taken and stored in 95% ethanol. DNA was extracted from the tissue samples and from the pools of D‐stage larvae from the two salinities using a Qiagen DNEasy Blood & Tissue kit with the standard protocol. DNA from the pools of larvae were further purified using a Zymo Clean and Concentrator kit, after which DNA amount was assessed using a QuBit BR assay. Sequencing libraries were prepared using the Illumina DNA PCR‐free protocol (https://www.illumina.com/products/by‐type/sequencing‐kits/library‐prep‐kits/dna‐pcr‐free‐prep.html) at the Swedish National Genomics Infrastructure. Due to low DNA amounts, successful libraries were prepared for 37 pools of larvae from 23‰ and from 35 families in 13‰ (of which 33 families were present in both salinities). Parents and larvae were multiplexed and sequenced in one lane of a S4‐600 flowcell in an Illumina NovaSeq6000 (2*150 bp reads). To increase coverage in the larval pools for accurate allele frequency estimations, libraries were additionally sequenced in two 10B‐300 flowcell lanes in an NovaSeqXPlus instrument (2*150 bp reads).

All bioinformatic analyses were performed on the Texas Advanced Computing Center's “LoneStar 6” supercomputer (https://tacc.utexas.edu/). All custom scripts and commands used in bioinformatic data analysis can be found here: https://github.com/DeWitP/gigas_lowsal_adaptation. Raw sequence data were quality‐trimmed and pruned of Nextera adapter sequences using Trim Galore (https://github.com/FelixKrueger/TrimGalore), after which they were mapped to the chromosome‐level assembly of Peñaloza et al. (2021) using BWA‐MEM (Li 2013). Duplicate reads were removed using Picard *MarkDuplicates* (https://broadinstitute.github.io/picard/), and InDel realignment was performed using GATK 3.6 (https://gatk.broadinstitute.org/). The three bam files for each larval pool were then merged into one, after which sequence coverage across the genome were plotted for each library using the *wgscoverageplotter* tool of the *jvarkit* package (https://github.com/lindenb/jvarkit).

The parental oysters were genotyped at SNP variant sites (InDels were excluded) using the *UnifiedGenotyper* tool in GATK 3.6. The vcf files were filtered using hard filters, as recommended by the Broad Institute (https://gatk.broadinstitute.org/hc/en‐us/articles/360035531112#2). Filters applied were: Normalised variant quality (QualByDepth (QD)) < 2.0; Variant quality (QUAL) < 30; Strand Odds Ratio (SOR) > 3.0; Fisher Strand (FS) > 60.0; Root mean square mapping quality (MQ) < 40.0; Mapping quality rank sum test (MQRankSum) < −12.5; Rank sum test for position within reads (ReadPosRankSum) < −8.0. Also, all loci where all parents were non‐reference homozygotes were removed. After this step, the *vcftools* package (https://vcftools.github.io) was used to keep only biallelic SNPs polymorphic in the parental dataset, no missing data allowed. Data for both alleles at all SNP loci identified in the parents were then extracted from the larval pools using *freebayes* (https://github.com/freebayes/freebayes) with the ‐pooled_continuous flag recommended for pooled samples of high but unknown ploidy, after which read counts for the reference and alternative alleles were extracted using the GATK *VariantsToTable* tool. Parental genotypes were also extracted from the vcf file to a genotype matrix using *vcftools* with the −012 flag. *vcftools* was also used on the parental genotype dataset with the ‐relatedness flag to examine relatedness among parents.

Deviations from Mendelian inheritance were examined in the two salinities using repeated G–tests of goodness‐of‐fit comparing observed and expected allele read counts in the larval pools for each family. Parental genotypes were used to calculate expected frequencies of reference and alternative alleles using R. Expected allele read counts were then obtained by multiplying the expected allele frequencies by the sequencing depth for each SNP locus. Only loci with both expected allele read counts > 5 were used for G‐tests (as this is an assumption of the test). G‐scores for individual SNP loci were calculated using the *RVAideMemoire* package in R. Individual G‐scores were then summed to Total G‐scores in 100‐Kb blocks along the chromosomes, along with information about the number of SNPs (degrees of freedom) used for the calculation. The relationship between the number of SNPs in a block and the Total G‐score was plotted in both salinities for each family using *ggplot2* in R. As the expected G‐score under the null hypothesis is equal to the sample size, total G‐scores for each block were standardised by dividing by the number of individual G tests within each block (G_S_). Thereafter, the effect of the low‐salinity treatment was separated from family‐level effects or bottle effects by subtracting G_S_ at reference salinity from G_S_ in low salinity for each family, generating a parameter from hereon called ∆G_S_ (the difference in deviation from Mendelian inheritance observed in reference vs low salinity, where a greater deviation in low salinity gives a positive ∆G_S_). Mean ∆G_S_ and bootstrapped 95% confidence intervals (1000 iterations) across all families (*n* = 33) were then calculated using the *boot* package and plotted using *ggplot2* in R for all 100‐Kb blocks along the genome.

In 100‐Kb blocks, where the lower bounds of the ∆G_S_ confidence intervals did not overlap the upper bounds of the main distribution, individual SNP locus ∆G scores were calculated along the length of the block. As above, only loci with both expected allele read counts > 5 in both salinities were used. Mean ∆G and bootstrapped 95% confidence intervals were then calculated and plotted for loci with data for a minimum of six families. Within‐block regions of high ∆G were then examined in the genome browser for annotations (https://www.ncbi.nlm.nih.gov/gdv/browser/genome/?id=GCF_902806645.1) and well annotated sequences inside the peak regions were BLASTed against the whole assembly in order to search for similar sequences elsewhere. In addition, the short‐read alignments in the peak regions were examined visually using IGV (Thorvaldsdóttir et al. 2013). Overviews of the alignments in the peak regions in Linkage Groups (LG = “chromosomes”) 6 and 9 (see results) were plotted in family tetrads (Female, Male, Larvae in 13‰, Larvae in 23‰) across all families.

To examine if deviations from Mendelian expectations were associated with the genotypes of the sperm or the eggs, individual SNP ∆G‐scores for loci within the two peak regions in LG6 and LG9 were also entered into a generalised linear model of the format:

$$\text{∆G}\sim \text{matrix}+\left(1\;|\;M\right)+\left(1\;|\;F\right)+\text{MHET}+\text{FHET}$$

where “matrix” = breeding block (*n* = 4, one block removed due to low sample size); M = male identity; F = female identity; MHET = male heterozygote (0/1); FHET = female heterozygote (0/1). This was implemented using the *lme4* package in R.

To search for signatures of recent selective sweeps or population bottlenecks in the high ∆G_S_ blocks, nucleotide diversity (π) and Tajima's D was calculated for 10‐Kb blocks along LG6 and LG9 in the oysters collected at the *present* invasion front using *vcftools*, after which they were plotted in R.

To observe variation in patterns of relatedness along the genomes of the parents from the *present* invasive front, we performed local PCA on continuous segment of the genomes. The *vcftools* package was used to convert the vcf file into a beagle file to run windowed analyses (sizes of 5000 SNPs) following the scripts available from the Therkildsen lab (https://github.com/therkildsen‐lab/genomic‐data‐analysis/blob/master/scripts/run_local_pca.sh). We used *PCAngsd* (Meisner and Albrechtsen 2018) to calculate a SNP covariance matrix for each window. Then, the R package *lostruct* (Li and Ralph 2019) was used to compute local PCA coordinates, calculate the distance matrix between windows and plot the resulting MDS (Multidimensional Scaling). The first five dimensions were displayed on the final plot.

## 3 Results

### 3.1 Fertilisation Success

**FIGURE 2:**
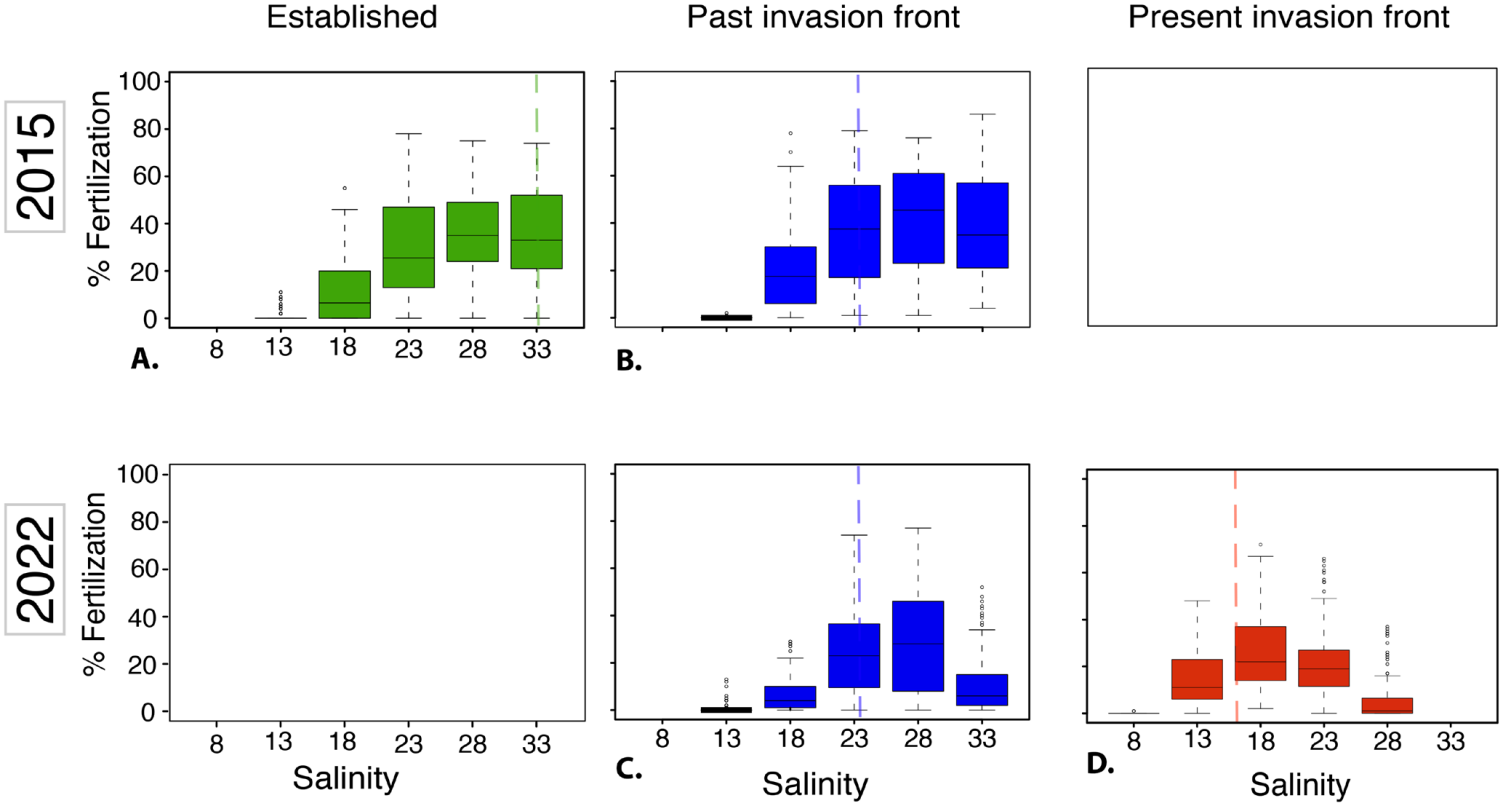
Fertilisation rates in Pacific oysters originating from an *established* site (green), *past* invasion front (blue), and *present* invasion front (red) with the fertilisation success (%) on the y‐axis and the different treatment salinities on the x‐axis. Panel A and B were experiments conducted in year 2015 and C and D were conducted in 2022. Dashed coloured lines in the panels indicate the salinity at which the parents were reared.

In 2015, the oysters from the *established* site had relatively high fertilisation success (median > 20%) in all salinities above 23‰ (note that sperm concentrations were adjusted to ensure maximum fertilisation success was 50% in the “native” salinity—see Methods). Fertilisation success was noticeably lower in 18‰ (median 5%), and very low, although not zero, in 13‰ (median 0%) (Figure 2A). A similar pattern was found for oysters from the *past* invasion front where median fertilisation success was higher than in oysters from the established site at all salinities except 33‰ (*cf* Figure 2A,B).

For the second round of experiments, conducted in 2022, the fertilisation success of oysters from the *past* invasion front was low at 33‰ (median < 5%), peaked at intermediate salinities of 23‰–28‰ (median > 20% fertilisation), was greatly reduced at 18‰ (median < 5% fertilisation), and almost non‐existent at 13‰ (median 0%) (Figure 2C). Oysters from the *present* invasion front, by contrast, showed low fertilisation success at 28‰ (median 0%), higher at 23‰ (median ca. 19%), peaked at 18‰ (median > 20%), and was much higher than in oysters from the *past* invasion front at 13‰ (median ca. 15%). At the lowest salinity, 8‰, only one family (one M × F combination) fertilised successfully, and then at very low rates (2%, Figure 2D). No obvious sire‐specific patterns in fertilisation rates could be observed (Data S1).

### 3.2 Quantitative Genetics and Heritability Estimation

**FIGURE 3:**
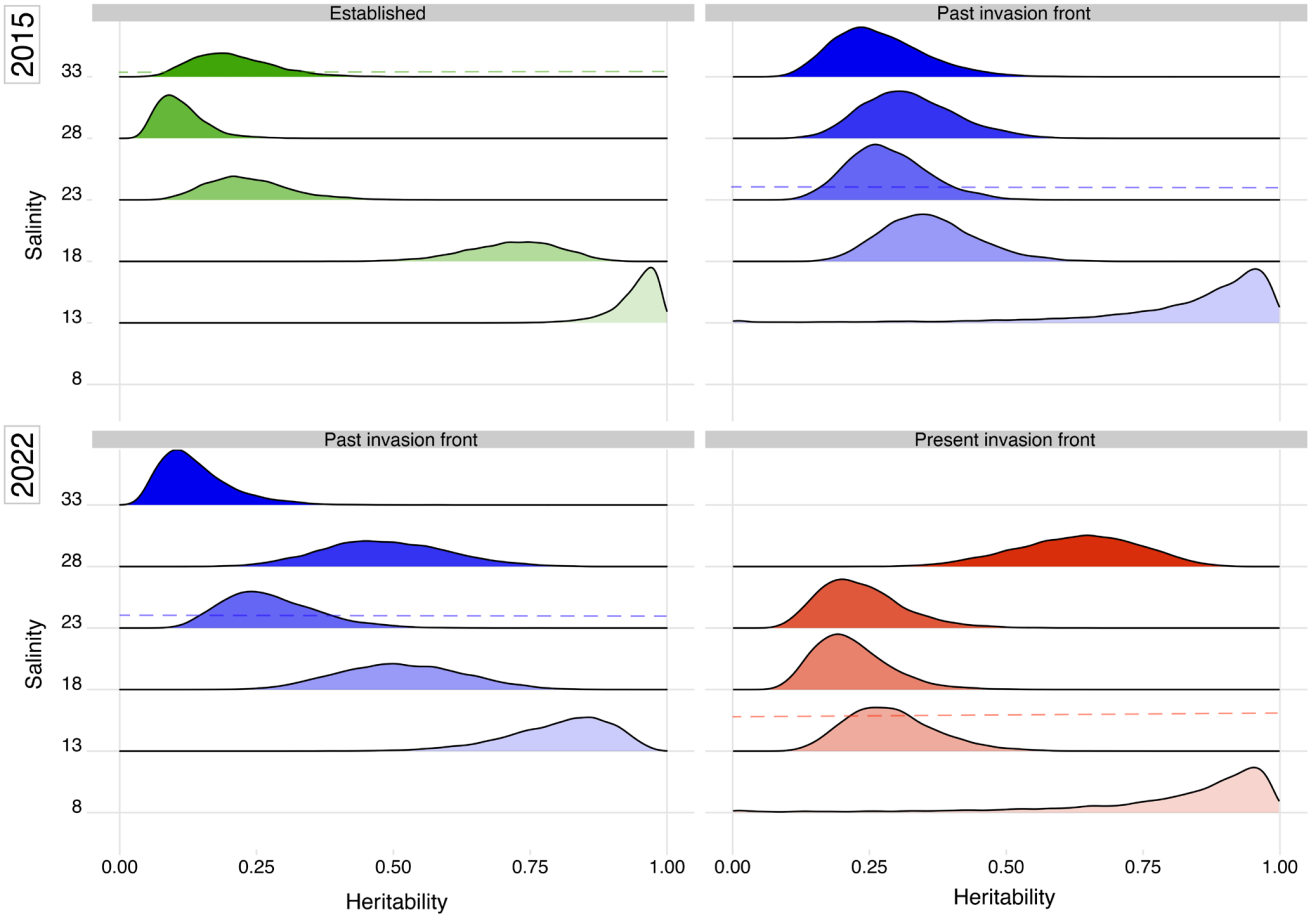
Heritability plots with posterior probability densities. Dashed coloured lines in the panels indicate the salinity at which the parents were reared (33‰, 23.5‰, and 15.9‰ for the established population, past invasion front, and present invasion front respectively).

We found that the heritability patterns of fertilisation success differed among sampling locations (Figure 3). The overall pattern observed was that heritabilities were always approaching 1 at extreme low salinities, while they were low‐intermediate in other treatments. We here report the 90% Highest Posterior Density Intervals (HPDIs; Data S2). In oysters from the *established* location we found that heritabilities were generally low (0.05–0.35) at high salinities (23‰–33‰), then exhibited an increase to 0.58–0.85 at 18‰ and to 0.89–1.00 at 13‰. For the population at the *past* invasion front in 2015 we found similar heritabilities (0.13–0.49) at all salinities except for the lowest (13‰), where *h* ^*2*^ increased to 0.58–1.00. However, when the experiments with oysters from the same location were repeated in 2022, we found that heritability had decreased somewhat in the lowest salinity (13‰), while it had increased at intermediate salinities (18‰–28‰). For oysters from the *present* invasion front we found low levels of heritability (0.11–0.42) at the three intermediate salinities (23‰, 18‰, and 13‰), higher heritability (0.44–0.80) in the highest salinity, and the highest heritability (0.52–1.00) at the lowest salinity (8‰). Genetic variances were consistently highest at the lowest salinities across all four sampled groups of oysters (Table 2, diagonals). The genetic correlations (Table 2, below diagonals) also showed trends, although less clearly. In general, there was a trend for correlations across salinities to be higher for more similar salinities (i.e., those nearer the diagonal) while correlations for more divergent salinities were often lower. There was little evidence of genetic trade‐offs in the form of negative genetic correlations except in two cases: performance of oysters from the *established* site in 2015 in the highest salinity covaried negatively with performance in salinities of 18‰ and 23‰ (90% HDPI: −0.63—0.09); and for oysters from the *present* invasion front performance in the lowest salinity (8‰) covaried negatively with performance in the highest salinity (28‰; 90% HDPI: −0.72—0.26, Data S3). See ridge plots, Data S2 for a better appreciation of the uncertainty in these correlation estimates.

**TABLE 2:** Genetic variances (dark blue), covariances (light blue), and correlations (green) for fertilisation success in five different salinities for different populations of *Crassostrea gigas.* Ambient/acclimatisation salinity for the oysters from the established site was 33‰, for the past invasion front was 23.5‰, and for the present invasion front was 15.9‰.

Established site 2015					
	Salinity 13	Salinity 18	Salinity 23	Salinity 28	Salinity 33
Salinity 13	45.7919	2.0782	−0.5151	0.5158	1.7837
Salinity 18	0.1619	4.3349	0.889	−0.0105	−0.3689
Salinity 23	−0.1076	0.6107	0.4816	0.1392	−0.1126
Salinity 28	0.204	−0.0073	0.4464	0.1912	0.1523
Salinity 33	0.4596	−0.2857	−0.2628	0.5244	0.4075
Past invasion front 2015					
	Salinity 13	Salinity 18	Salinity 23	Salinity 28	Salinity 33
Salinity 13	21.6094	0.9206	0.3492	0.3895	0.5058
Salinity 18	0.2632	0.8018	0.4685	0.2729	0.2048
Salinity 23	0.1204	0.7019	0.555	0.5042	0.1974
Salinity 28	0.1141	0.3745	0.8181	0.677	0.1673
Salinity 33	0.1826	0.321	0.3714	0.2851	0.5205
Past invasion front 2022					
	Salinity 13	Salinity 18	Salinity 23	Salinity 28	Salinity 33
Salinity 13	8.5393	1.934	0.5618	0.4577	0.5093
Salinity 18	0.5224	1.7836	0.3919	−0.033	0.0498
Salinity 23	0.2631	0.3852	0.6053	0.0409	0.0852
Salinity 28	0.1173	−0.0204	0.0354	1.5826	0.0905
Salinity 33	0.3903	0.086	0.2219	0.1474	0.2534
Present invasion front 2022					
	Salinity 8	Salinity 13	Salinity 18	Salinity 23	Salinity 28
Salinity 8	18.5973	0.5543	0.1242	−0.2696	−1.5039
Salinity 13	0.1925	0.5976	0.2881	0.1648	0.4625
Salinity 18	0.0539	0.5883	0.4032	0.1948	−0.024
Salinity 23	−0.1172	0.3202	0.4585	0.4512	−0.0552
Salinity 28	−0.2511	0.3546	−0.0244	−0.0545	2.7726

### 3.3 Genomics

Sequencing coverage across the parents from the *present* invasion front was ca. 5x, while for the larval pools mean coverage was about 14‐20x. Coverage plots for all samples can be found at: https://github.com/DeWitP/gigas_lowsal_adaptation/tree/main/mapping_to_variant_detection_LoneStar6/coverage_plots. All alignment and duplicate statistics can be found in the supplemental data (Data S4). A total of 22,132,741 SNPs were genotyped in the parents from the *present* invasion front after all filtering steps. Of these, allele read counts could be extracted from the larval pools for 19,142,908 SNPs in the 33 families which were sequenced in both low (13‰) and “reference” (23‰) salinity. All parents were found to be unrelated to each other (Data S5).

**FIGURE 4:**
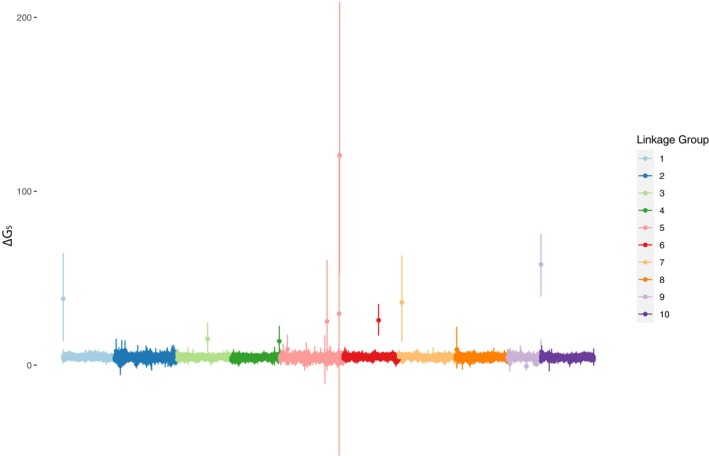
∆G_S_ scores for 100‐Kb blocks along the 10 linkage groups (=chromosomes) of the genome assembly (shown in different colours for clarity), with bootstrapped (*n* = 1000) 95% confidence intervals. G_S_ scores were calculated for oyster larval (24 h old) full‐sib pools (*n* = 33 families) from the *present* invasion front grown in a low salinity treatment (13‰) and a control salinity (23‰) by first calculating the Total G‐score for all SNPs per 100‐Kb block (the deviation between the observed allele frequency and expectation given parental genotypes), then standardising by the number of SNP loci per block. ∆G_S_ scores were then calculated as: ∆G_S_ = G_S_(treatment) ‐ G_S_(control) for each family, to control for all other factors than treatment.

**FIGURE 5:**
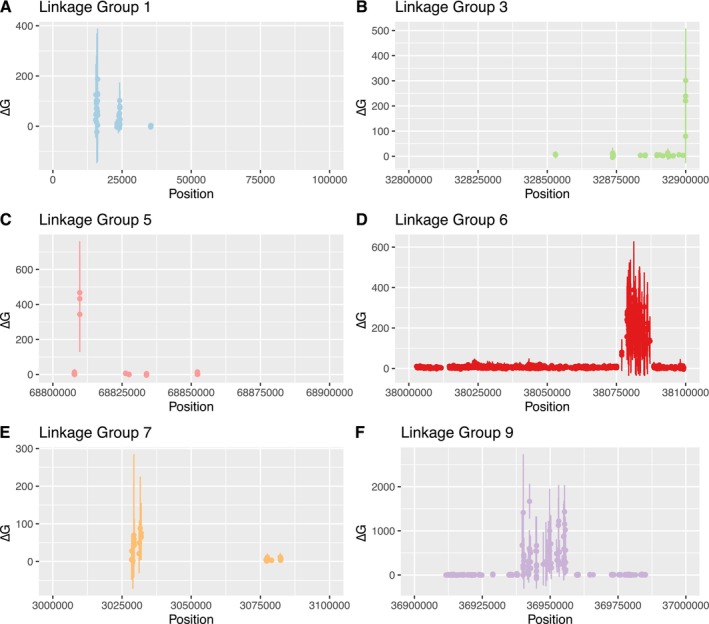
∆G scores for individual SNP loci within high ∆G_S_ blocks (see Figure 4) in larval (24 h old) oysters from the *present* invasion front, grown in a low salinity treatment (13‰) and a control salinity (23‰). Individual G scores (the deviation between the observed allele frequency and expectation given parental genotypes) were calculated for SNPs for each full‐sib family in loci with > 5 expected read counts of both alleles in both treatment and control. ∆G scores were then calculated as ∆G = G(treatment) ‐ G(control), to control for all other factors than treatment, in loci with G scores for a minimum of 6 families. The plot shows mean ∆G scores across families with bootstrapped (*n* = 1000) 95% confidence intervals. The LG6 block contains multiple histones, and the LG9 block contains the ribosomal SSU and LSU according to the genome reference annotation. Remaining blocks contain no clearly annotated features.

Mean ∆G_S_ scores across families were slightly positive but close to zero genome‐wide (Data S6), and Total G‐scores scaled with the number of loci with steeper slopes in low‐salinity than in reference salinity for most families (Data S7). However, six 100‐Kb blocks spread throughout the genome had confidence intervals which were completely non‐overlapping with the main ∆G_S_ distribution (Figure 4). These six blocks were all located in different chromosomes (linkage groups): LG1, LG3, LG5, LG6, LG7 and LG9. In four of these, the within‐block distribution of SNP loci was highly patchy (Figure 5A–CE), although some indications could be seen of peaks at positions LG1:15,421‐24,264, LG3:32,899,948‐32,899,999, LG5:68,809,729‐68,809,741 and LG7:3,028,542‐3,031,907. Two of the blocks had a more even within‐block distribution of data, and clear peaks could be observed at LG6:38,076,916‐38,087,110 and LG9:36,939,689‐36,955,733 (Figure 5D,F). The peak in LG6 corresponds to a protein‐coding region coding for the entire Nucleosome (Histones H2A, H2B, H3 and H4) as well as linker Histone H1. The peak region in LG9 contains both Large and Small Ribosomal Subunit (LSU and SSU) rDNA. In BLAST searches of the genome assembly, no other copies of ribosomal DNA were found. One other region containing several copies of histones H1, H2A, H2B and H3 was found at LG7:18,956,000—19,000,000. This region did not exhibit an elevated ∆G_S_ in our data, however.

Generalised linear mixed modelling showed that in both peak regions in LG6 and LG9, the genotype of the sire was the strongest determinant of the ∆G score (*p* = 0.007 in the LG6 peak; *p* = 9*10^−8^ in the LG9 peak), while the genotype of the mother was not significant in either of the peak regions (*p* = 0.50 in LG6; *p* = 0.11 in LG9; all model output is given in Data S8). An examination of the short‐read alignments in both of these regions showed that sequencing depth was orders of magnitude higher inside the peak regions than in surrounding genomic regions (Data S9), suggesting that these regions might consist of tandem repeated arrays encoding multiple copies of histones and rDNA which have been incorrectly collapsed in the reference genome assembly.

Nucleotide diversity (π) and Tajima's D did not exhibit any pattern of being different in or near the high ∆G_S_ blocks (red dots in Data S10), although a general reduction of genetic diversity was observed at the end of LG9 (Data S10D).

Although there was considerable variation in eigenvector loading along the chromosomes in the results from the local PCA (Data S11), there were no signals of chromosomal rearrangements (chromosomal blocks of large numbers of linked SNPs with deviating eigenvector loadings) observed across the genomes of the parents from the current invasion front.

## 4 Discussion

We found evidence that invading Pacific oysters ( *C. gigas* ) on the Swedish west coast are increasingly able to reproduce in new, lower salinity, conditions at the advancing invasion front. Specifically, our results show that oysters close to the *present* invasion front (salinity 15.9‰), and oysters in a location which was first colonised ≤ 15 years ago (*past* invasion front, salinity 23.5‰), were better able to reproduce in low salinities than oysters from the *established* site (35.3‰). At the *present* invasion front we observed maximum fertilisation success at 18‰, moderate fertilisation at 13‰, and very low (but non‐zero) levels of fertilisation at 8‰. This result is striking considering the lower reproductive limit of *C. gigas* has been reported to be 15‰ (Wiltshire 2007; Nehring 2011), and indeed we found that oysters from an *established* site in the English Channel showed little, to no, ability to fertilise successfully in salinities below 18‰.

Given the magnitude and speed of these changes, it is reasonable to wonder whether the main cause of this phenotypic shift might be plasticity rather than genetic adaptation—not least because the high dispersal capacity of oyster larvae makes it more difficult for locally adapted populations to evolve. Prior work on *C. gigas* in their native range has, however, found adaptation to local environments over surprisingly small spatial scales (Li et al. 2018), suggesting that some mechanism might act to prevent long‐distance gene flow. This balance between plasticity and adaptation is supported by our heritability estimates, which showed that in the central salinity range typically experienced by *C. gigas* , additive genetic variation only accounted for a modest part (*h* ^*2*^ ≈ 25%) of the observed differences in fertilisation rates, whereas when oysters experienced salinities outside of this range, the response was almost exclusively genetically determined (Figure 3). The negative genetic correlations between high and low salinity treatments seen at the *present* invasion front (Table 2) also indicate genetic trade‐offs, where segregation of alleles permitting fertilisation in low salinities seems to impose genetic constraints on performance in saltier conditions. However, the imprecision in our estimates of genetic correlations makes it hard to draw firm conclusions about the relative magnitudes of correlations, as does the fact that this pattern is not consistent across oysters from all four locations.

Almost all previous work on salinity tolerances of oysters has focused on the Eastern oyster ( *Crassostrea virginica* ), where small‐scale local phenotypic differences have been found both in adults (McCarty et al. 2020; Swam et al. 2022) and larvae (Eierman and Hare 2013; Scharping et al. 2019). Sharping et al. (2019) found that the fitness optimum of larvae from a mesohaline estuary was shifted by 7‰ compared to larvae from a nearby polyhaline estuary, while Eierman and Hare (2013) found local differences in larval tolerance even within the same estuary, driven by salinity of the parental environment. Both of these studies suggest an ability for rapid local adaptation in this species. As in our study of *C. gigas* , salinity tolerance in *C. virginica* has been found to be determined genetically to a large extent. McCarty et al. (2020) found that acute low‐salinity tolerance in adults was heritable (*h* ^*2*^ = 0.34–0.59 in two different experiments), and Griffiths et al. (2021) showed that transgenerational plasticity had little effect on low‐salinity survival and growth of larvae, but that there was a strong genetic component (*h* ^*2*^ = 0.68 ± 0.25; mean ± 95% CI). Similarly, Sirovy et al. (2021) found that while plasticity in salinity tolerance in adult oysters was generally high, it did not differ among individuals. As a consequence, genotype‐by‐environment (GxE) interactions were low, constraining the evolution of differences in plasticity.

Our genomic analysis of families from the *present* invasion front identified two regions which consistently exhibited a stronger deviation from Mendelian inheritance in low salinity than in the control. One of these, a 10 kB‐block containing annotated genes coding for the nucleosome, was located on linkage group 6. The other, a 16 kB‐block containing annotated genes coding for the ribosome, was located on linkage group 9. Both histones and ribosomal DNA are known to typically occur as tandemly repeated arrays in genomes (e.g., Roehrdanz et al. 2010), something not observed in the used reference genome sequence, raising the possibility that the two identified blocks actually consist of multiple copies which have been incorrectly collapsed into one sequence in the reference. Our observations of increased sequencing depth in both blocks compared to surrounding regions (see Data S9) are consistent with this idea. While this does not explain our observations of differences in deviation from Mendelian inheritance across treatments, it makes it impossible to identify individual SNPs possibly involved in low salinity tolerance, as we cannot tell apart SNP variation from variation among different gene copies using the current reference assembly.

We also found that the paternal (but not the maternal) genotype had a significant influence on the difference in deviation from Mendelian inheritance between low salinity and control in oysters at the *present* invasion front. This suggests that the genetic composition of the sperm pool is the main target of natural selection in low‐salinity conditions. One hypothesis that might explain this observation could be that the ribosome and nucleosome of the sperm might affect their ability to maintain swimming capacity in low salinity. Sperm motility in *C. gigas* from the *past* invasion front has been shown to be negatively affected by reduced salinity (albeit not greatly and with considerable variability among males; average motility in 13‰ was ~75% of that in 33‰; Falkenberg et al. 2019). Broadly equivalent results have been obtained in *C. virginica* (Nichols et al. 2021; MacKenzie Tackett et al. 2024). The extent to which these changes are due to ribosomal, or nucleosomal, structure is unknown. In many species (including many molluscs) the histones that normally bind the DNA to form chromatin in somatic cells are replaced in sperm by a series of sperm nuclear basic proteins (SNBP). These SNBP's further condense the chromatin, better protect the sperm DNA from damage (by, for example, low salinity), and greatly reduce the volume of the sperm nucleus (Török and Gornik 2018). In *C. gigas* sperm, however, SNBP's are reported to be absent and the DNA is packaged by histones (Sellos 1985). Given that sperm motility varies markedly among individual *C. gigas* (sperm of some males responded *positively* to reduced salinity; Falkenberg et al. 2019), it would be highly informative to compare the sperm swimming performance of individuals/lineages with high vs low salinity tolerance.

Notwithstanding our clear genomic evidence that the nucleosome and the ribosome appear to be under low‐salinity selection in males at the *present* invasion front, there are additional processes that can influence fertilisation success in low salinities. For example, it has been shown that the genomic vesicle of the *C. gigas* egg can break down more slowly in low salinity (Li et al. 2021; also in *C. rhizophorae* , Lopes et al. 2024). Effects of salinity on ribosomal protein synthesis can also have consequences for fertilisation. Gong and Li (2023) found ribosomal downregulation in low salinity in *C. nippona*, and identified a number of potential ribosomal low‐salinity stress markers. Finally, it is important to consider that selection will occur at timepoints other than fertilisation. It has been found that the environmental tolerances of sperm and offspring can co‐vary, and that experience of the sperm both inside and outside the father can cause phenotypic shifts in the offspring (Marshall 2015). More research is needed to clarify this phenomenon.

Considering that the genomic data was generated from just one generation of recombination from the parental generation, it is interesting that the spatial resolution of these data is quite high, identifying regions under low‐salinity selection in the size range of 10 kB. As the genomic data from the larval pools consist of reads from pools of a large number of larvae from each family, there could have occurred hundreds of recombination events in each pool, increasing our spatial resolution. In addition, as we calculated means across families, differences in the location of recombination events in the different families may also have increased our spatial resolution. The observation that the identified regions might represent collapsed arrays of tandemly repeated regions could also explain the seemingly high recombination here. Recent studies in a variety of different species have found chromosomal rearrangements to be often involved in rapid local adaptation (e.g., Barth et al. 2017; Mérot et al. 2018; Koch et al. 2021). While we did not observe evidence of large‐scale structural variants with divergent evolutionary histories (e.g., inversions) in our data, we cannot rule out smaller‐scale processes being important. For example, we were not able to investigate gene expansion or reductions in the histone‐ and ribosomal‐coding regions due to the potential merging of gene copies in the reference genome. Importantly though—any chromosomal rearrangements occurring during meiosis would be expected to be inherited equally by larvae in both salinity treatments unless there was a differential selective pressure applied to these chromosomal regions across treatments.

Furthermore, we found no clear signs of selective sweeps in the high ∆G regions in the oysters collected at the current invasion front (e.g., a reduction in genetic diversity). When comparing overall heterozygosity of the oysters at the invasion front to an unpublished RAD dataset including Pacific oysters from across northern Europe (Per Erik Jorde, pers.comm.), no differences were observed. The absence of signs of recent selection is surprising considering the strong selective pressure imposed by the low salinity on fertilisation rates, however, Pacific oysters are known to be immensely malleable to evolution, with very high genetic diversity including also a high genetic load (Plough et al. 2016). Moreover, Pacific oysters have rather high recombination rates—even with hundreds of meiotic events in each larval pool and potentially collapsed repeated arrays, our observations of ∆G peaks in the 10‐kB size range in just one generation is quite surprising. This biological feature might facilitate the rapid adaptation of *C. gigas* to novel environments without exhibiting obvious genetic signatures of selective sweeps or bottlenecks.

Invasive species are one of several key drivers of the current biodiversity crisis. Being able to predict future invasions, as well as the limits of the adaptive capabilities of invaders in novel environments, will be crucial to management on local, regional, and global scales. Here, we show that Pacific oysters ( *C. gigas* ) at the *present* invasion front at the entrance to the Baltic Sea have the capacity to evolve to reproduce at lower salinities than previously reported. This indicates the potential for *C. gigas* to colonise the Baltic Sea in the near future. We also found none of the classic signs of reduced genetic diversity at an invasion front, indicating that Pacific oysters in western Sweden still have a strong capacity to adapt further to new environments. Collectively, these results argue strongly that the species should be monitored closely for further spread. A variety of marine species have successfully colonised the Baltic Sea since its opening ca. 10,000 years ago, and many of these show a strong genetic cline precisely where the *present* invasion front of the Pacific oyster is currently located (Johannesson et al. 2020), indicating that adaptation from marine to brackish environments is possible over relatively short timescales. The ramifications of a Pacific oyster invasion for the biodiversity of the Baltic are difficult to assess at present, but as the Baltic is already a low‐diversity region, with a relatively simple food web (Reusch et al. 2018), the consequences could be dramatic.

## Author Contributions

Alexandra Kinnby, Jonathan N. Havenhand and Pierre De Wit designed the research. Alexandra Kinnby, Jonathan N. Havenhand and Chloé Robert performed the experimental work. Alexandra Kinnby, Chloé Robert, Pierre De Wit, Jonathan N. Havenhand and Luc Bussière performed data analyses. Alexandra Kinnby, Pierre De Wit and Jonathan N. Havenhand wrote the initial version of the manuscript. All co‐authors contributed to the final version of the text.

## Conflicts of Interest

The authors declare no conflicts of interest.

## Supporting information


**Data S1**. Fertilisation rates per sire across the different treatments in the different sampled study areas. For clarity, sires for each site are sorted by performance in the highest salinity.

**Data S2**. 90% HDPI intervals for the posterior densities of the heritability and the genetic correlation estimates.

**Data S3**. Ridge plots showing posterior density of all pairwise genetic correlations across salinity treatments in the four groups of oysters.

**Data S4**. Alignment and duplicate read statistics for all samples included in the study. Note that all larval full‐sib families were sequenced in three replicates.

**Data S5**. Relatedness among sequenced parents from the current invasion front. The estimate ranges from 0 (unrelated) to 1 (clones).

**Data S6**. Histogram of the distribution of ∆G scores in all families.

**Data S7**. G‐scores as a function of number of loci per block in salinity 13 (red) and 23 (blue).

**Data S8**. Output of linear models testing the effect of male and female heterozygosity on the ∆G values in the peak regions of LG6 (left) and LG9 (right).

**Data S9**. Alignment summary plots of the two annotated high ∆G peak regions in LG6 and LG9 showing sequencing depth along the reference sequence as well as sequence divergence from the reference sequence (highlighted in colour:). The sequencing depth scale for each plot (min‐max) is given in brackets on the top left of each plot. Plots are organised in family tetrads for each family from crosses of oysters from the *present* invasion front, from top to bottom: Female (Mother), Male (Father), Larval full‐sib family in salinity 13‰, Larval full‐sib family in 23‰.

**Data S10**. Nucleotide diversity and Tajima’s D along LG6 and 9.

**Data S11**. Local PCA across the genomes of the parents from the current invasion front, for each scaffold. Window size of 10,000 SNPs. The first five dimensions of an MDS on the eigenvector loadings are shown.

## Data Availability

Raw data on fertilisation rates are available in supplement; all raw genomic data are available on NCBIs Short Read Archive under BioProject PRJNA1190893. Variant call sets (vcf files for parents and larvae) are available on Dryad and can be cited as: [dataset] Kinnby et al. 2024; Low‐salinity adaptation in Pacific oysters; Dryad: https://doi.org/10.5061/dryad.v6wwpzh5q.
