# Supplementary material for: The Roles of Plasticity and Selection in Rapid Phenotypic Changes at the Pacific Oyster Invasion Front in Europe
Source: Mol Ecol. 2025 Feb 7;34(23):e17684. doi: 10.1111/mec.17684 (PMC12684338; doi:10.1111/mec.17684)
Supplement: Supplementary file 1 — Data S1. Fertilisation rates per sire across the different treatments in the different sampled study areas. For clarity, sires for each site are sorted by performance in the highest salinity. [file MEC-34-e17684-s007.pdf]

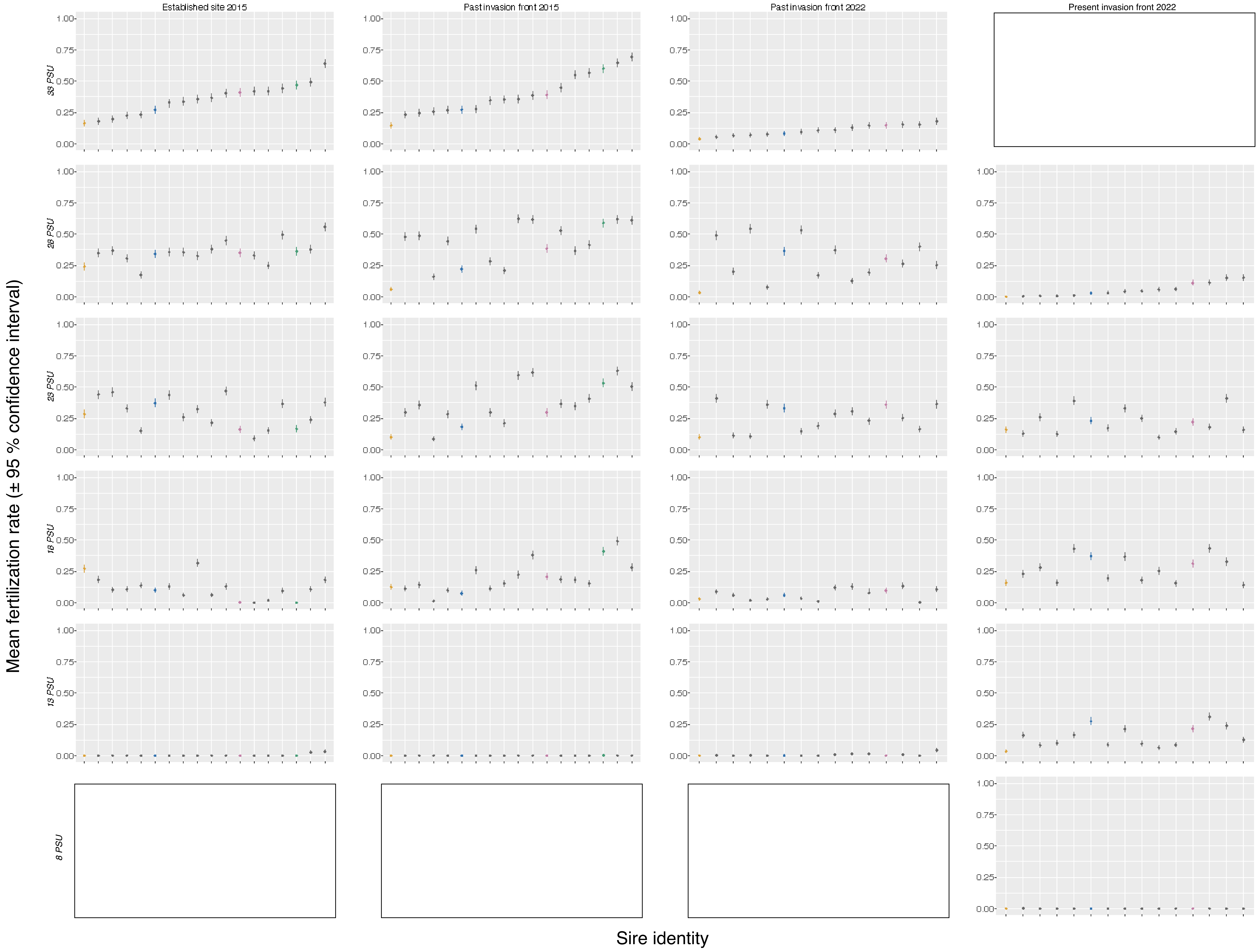

Supplementary Data S1. Fertilization rates per sire across the different treatments in the different sampled study areas. For clarity, sires for each site are sorted by performance in the highest salinity.
