# Supplementary material for: The Roles of Plasticity and Selection in Rapid Phenotypic Changes at the Pacific Oyster Invasion Front in Europe
Source: Mol Ecol. 2025 Feb 7;34(23):e17684. doi: 10.1111/mec.17684 (PMC12684338; doi:10.1111/mec.17684)
Supplement: Supplementary file 2 — Data S2. 90% HDPI intervals for the posterior densities of the heritability and the genetic correlation estimates. [file MEC-34-e17684-s009.docx]

Supplementary Data S2. 90 % HDPI intervals for the posterior densities of the heritability and the genetic correlation estimates.

| **Population** | **Salinity** | **Lower hdpi** | **Upper hdpi** |
| --- | --- | --- | --- |
| Established 2015 | 13 | 0.887 | 0.996 |
| Established 2015 | 18 | 0.58 | 0.852 |
| Established 2015 | 23 | 0.111 | 0.353 |
| Established 2015 | 28 | 0.045 | 0.171 |
| Established 2015 | 33 | 0.0932 | 0.324 |
| Past invasion front 2015 | 13 | 0.576 | 0.998 |
| Past invasion front 2015 | 18 | 0.219 | 0.494 |
| Past invasion front 2015 | 23 | 0.165 | 0.402 |
| Past invasion front 2015 | 28 | 0.182 | 0.456 |
| Past invasion front 2015 | 33 | 0.134 | 0.398 |
| Past invasion front 2022 | 13 | 0.663 | 0.954 |
| Past invasion front 2022 | 18 | 0.328 | 0.691 |
| Past invasion front 2022 | 23 | 0.139 | 0.408 |
| Past invasion front 2022 | 28 | 0.297 | 0.666 |
| Past invasion front 2022 | 33 | 0.0405 | 0.232 |
| Present invasion front 2022 | 8 | 0.519 | 0.996 |
| Present invasion front 2022 | 13 | 0.161 | 0.423 |
| Present invasion front 2022 | 18 | 0.109 | 0.323 |
| Present invasion front 2022 | 23 | 0.114 | 0.354 |
| Present invasion front 2022 | 28 | 0.441 | 0.805 |

90% Highest Posterior Density Intervals (HPDIs) for heritabilities

90% Highest Posterior Density Intervals (HPDIs) for genetic correlations

| population | Treats | lower_hdpi | upper_hdpi |
| --- | --- | --- | --- |
| Established 2015 | cor_s… | -0.272 | 0.613 |
| Established 2015 | cor_s… | -0.557 | 0.399 |
| Established 2015 | cor_s… | -0.258 | 0.633 |
| Established 2015 | cor_s… | 0.0846 | 0.876 |
| Established 2015 | cor_s… | 0.352 | 0.874 |
| Established 2015 | cor_s… | -0.434 | 0.442 |
| Established 2015 | cor_s… | -0.631 | 0.0922 |
| Established 2015 | cor_s… | 0.137 | 0.789 |
| Established 2015 | cor_s… | -0.597 | 0.0647 |
| Established 2015 | cor_s… | 0.223 | 0.835 |
| Past invasion front 2015 | cor_s… | -0.214 | 0.777 |
| Past invasion front 2015 | cor_s… | -0.405 | 0.624 |
| Past invasion front 2015 | cor_s… | -0.412 | 0.630 |
| Past invasion front 2015 | cor_s… | -0.286 | 0.728 |
| Past invasion front 2015 | cor_s… | 0.519 | 0.890 |
| Past invasion front 2015 | cor_s… | 0.0698 | 0.701 |
| Past invasion front 2015 | cor_s… | 0.00202 | 0.647 |
| Past invasion front 2015 | cor_s… | 0.693 | 0.948 |
| Past invasion front 2015 | cor_s… | 0.0773 | 0.684 |
| Past invasion front 2015 | cor_s… | -0.0411 | 0.629 |
| Past invasion front 2022 | cor_s… | 0.187 | 0.881 |
| Past invasion front 2022 | cor_s… | -0.125 | 0.679 |
| Past invasion front 2022 | cor_s… | -0.295 | 0.547 |
| Past invasion front 2022 | cor_s… | -0.00612 | 0.820 |
| Past invasion front 2022 | cor_s… | 0.0453 | 0.729 |
| Past invasion front 2022 | cor_s… | -0.395 | 0.381 |
| Past invasion front 2022 | cor_s… | -0.288 | 0.497 |
| Past invasion front 2022 | cor_s… | -0.398 | 0.442 |
| Past invasion front 2022 | cor_s… | -0.166 | 0.615 |
| Past invasion front 2022 | cor_s… | -0.287 | 0.593 |
| Present invasion front 2022 | cor_s… | 0.313 | 0.853 |
| Present invasion front 2022 | cor_s… | -0.0254 | 0.674 |
| Present invasion front 2022 | cor_s… | 0.0187 | 0.715 |
| Present invasion front 2022 | cor_s… | 0.143 | 0.776 |
| Present invasion front 2022 | cor_s… | -0.431 | 0.373 |
| Present invasion front 2022 | cor_s… | -0.46 | 0.332 |
| Present invasion front 2022 | cor_s… | -0.326 | 0.738 |
| Present invasion front 2022 | cor_s… | -0.489 | 0.603 |
| Present invasion front 2022 | cor_s… | -0.666 | 0.450 |
| Present invasion front 2022 | cor_s… | -0.78 | 0.264 |
