# Supplementary material for: The Roles of Plasticity and Selection in Rapid Phenotypic Changes at the Pacific Oyster Invasion Front in Europe
Source: Mol Ecol. 2025 Feb 7;34(23):e17684. doi: 10.1111/mec.17684 (PMC12684338; doi:10.1111/mec.17684)
Supplement: Supplementary file 3 — Data S3. Ridge plots showing posterior density of all pairwise genetic correlations across salinity treatments in the four groups of oysters. [file MEC-34-e17684-s010.pdf]

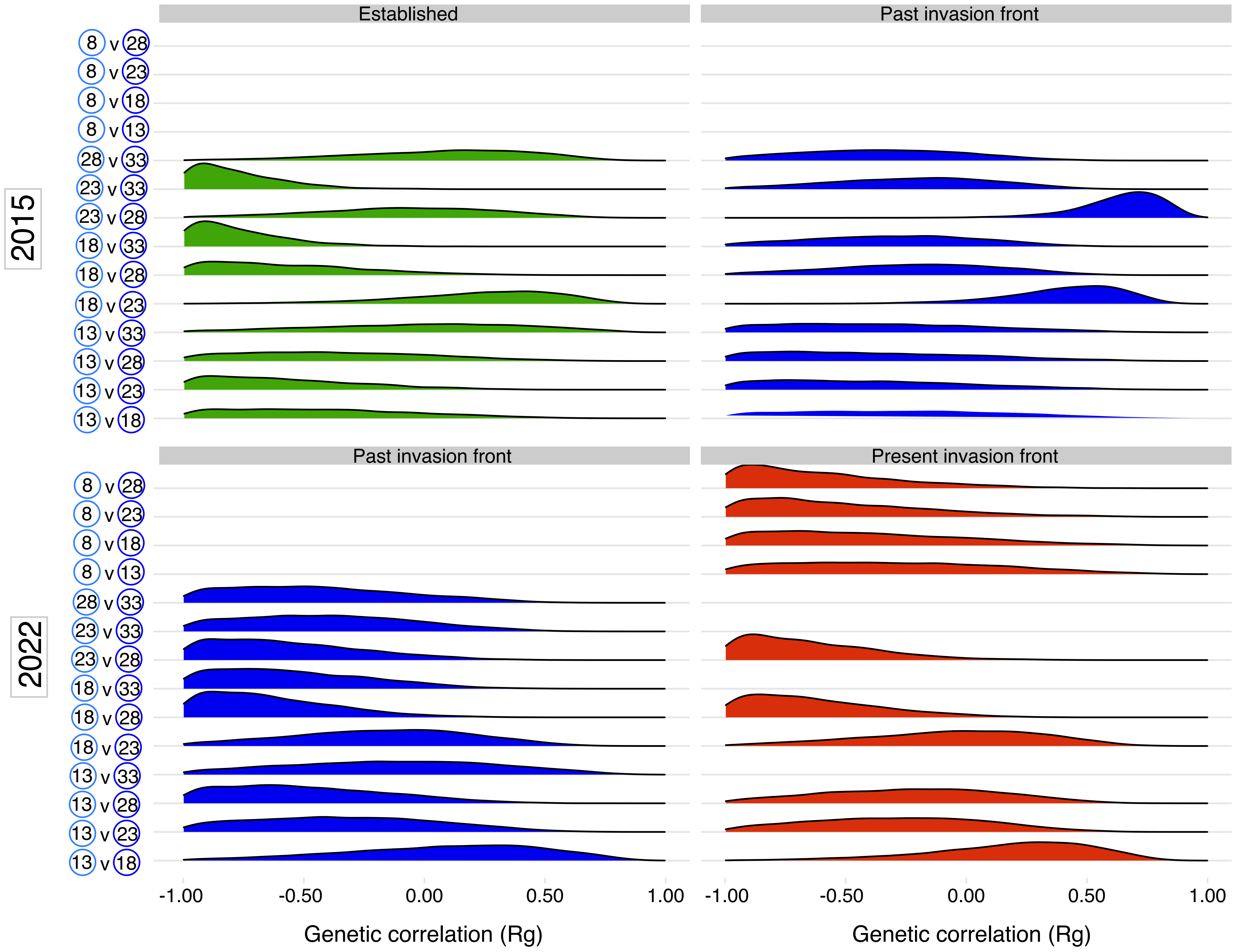

Supplementary Data S3. Ridge plots showing posterior density of all pairwise genetic correlations across salinity treatments in the four groups of oysters.
