# Supplementary figures and images for: The Roles of Plasticity and Selection in Rapid Phenotypic Changes at the Pacific Oyster Invasion Front in Europe
Source: Mol Ecol. 2025 Feb 7;34(23):e17684. doi: 10.1111/mec.17684 (PMC12684338; doi:10.1111/mec.17684)

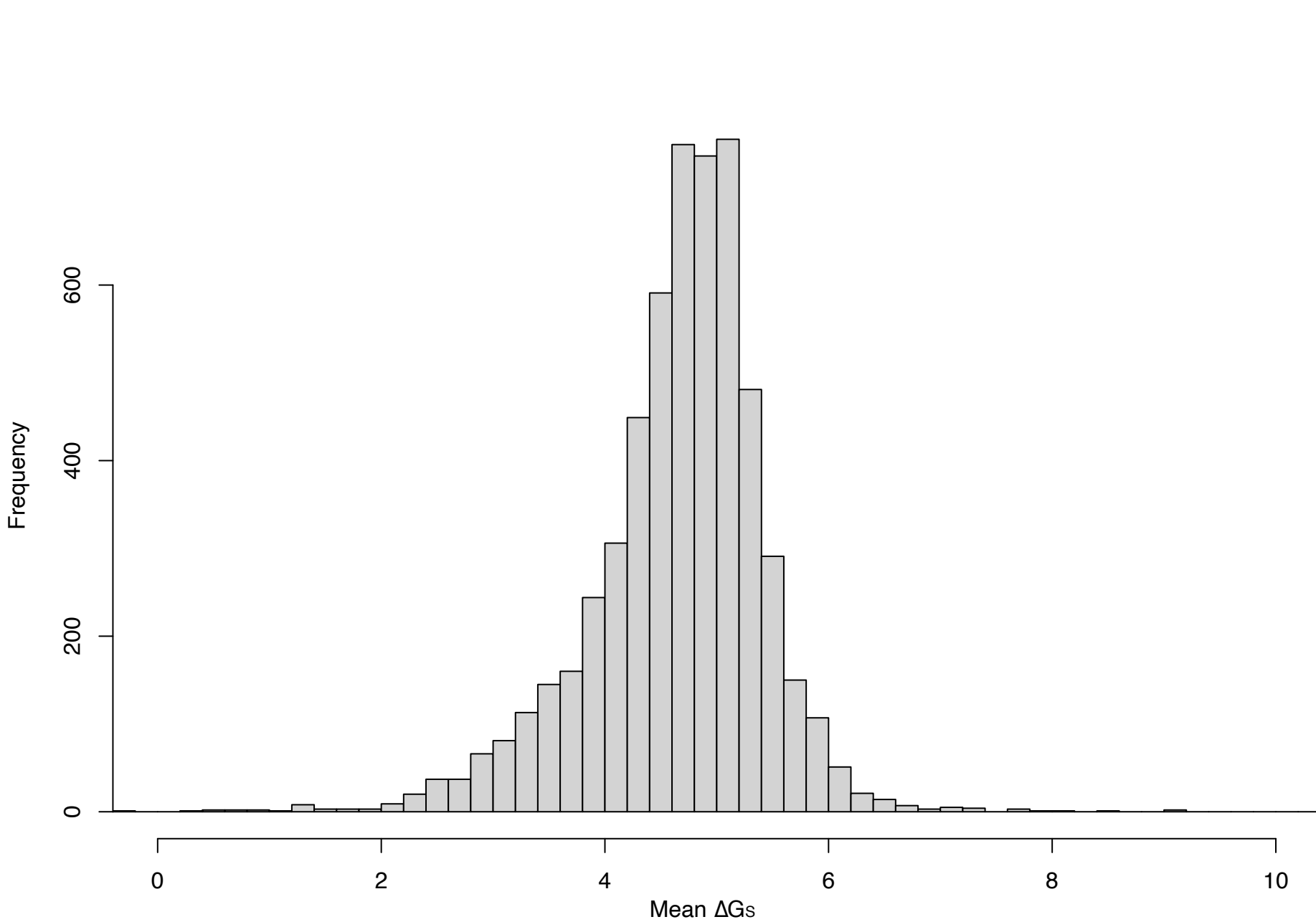

Supplementary Data S6. Histogram of the distribution of  $\Delta G$  scores in all families.

Supplement: Supplementary file 6 — Data S6. Histogram of the distribution of ∆G scores in all families. [file MEC-34-e17684-s003.pdf]

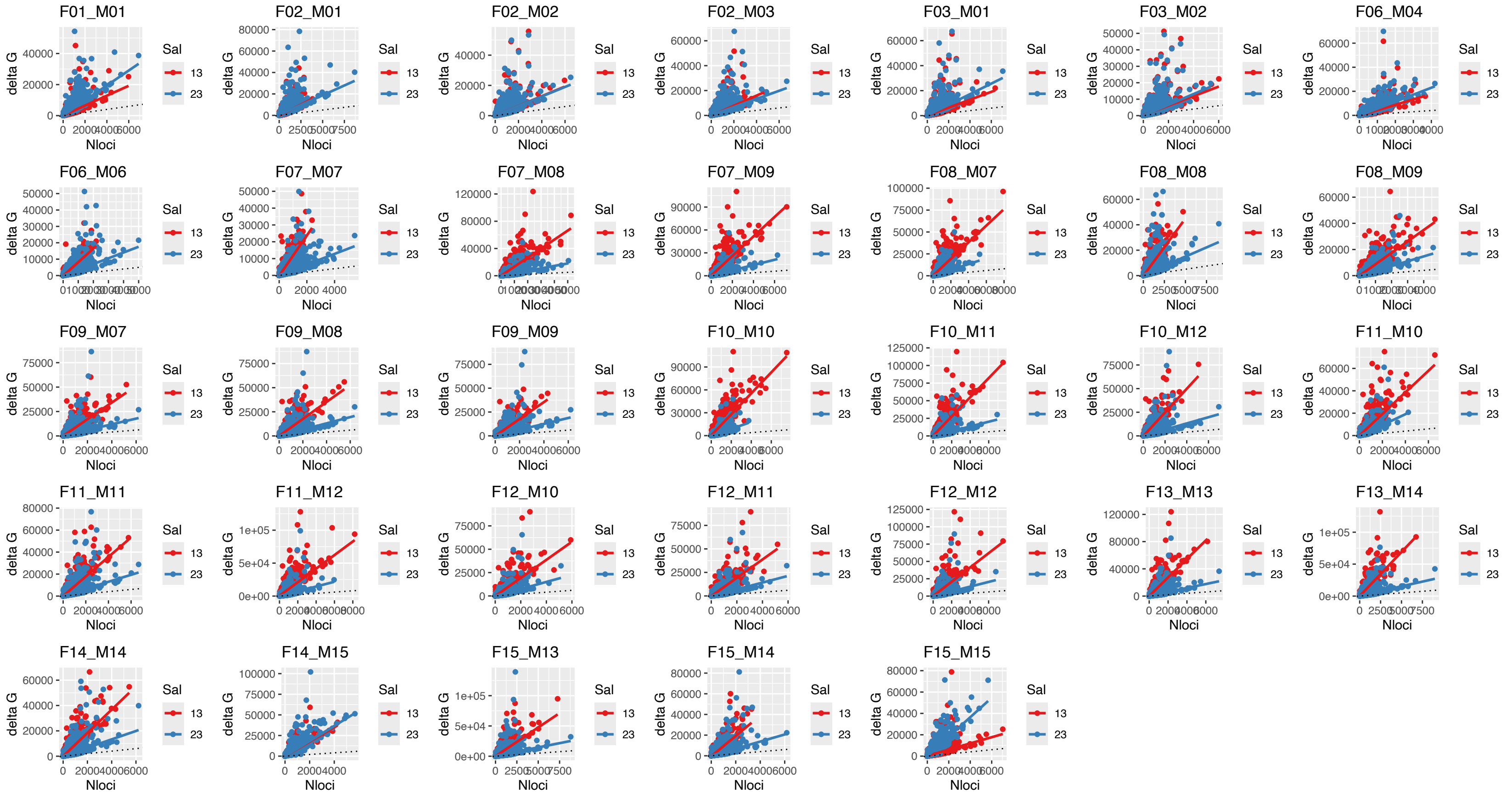

Supplementary Data S7. G-scores as a function of number of loci per block in salinity 13 (red) and 23 (blue).

Supplement: Supplementary file 7 — Data S7. G‐scores as a function of number of loci per block in salinity 13 (red) and 23 (blue). [file MEC-34-e17684-s006.pdf]

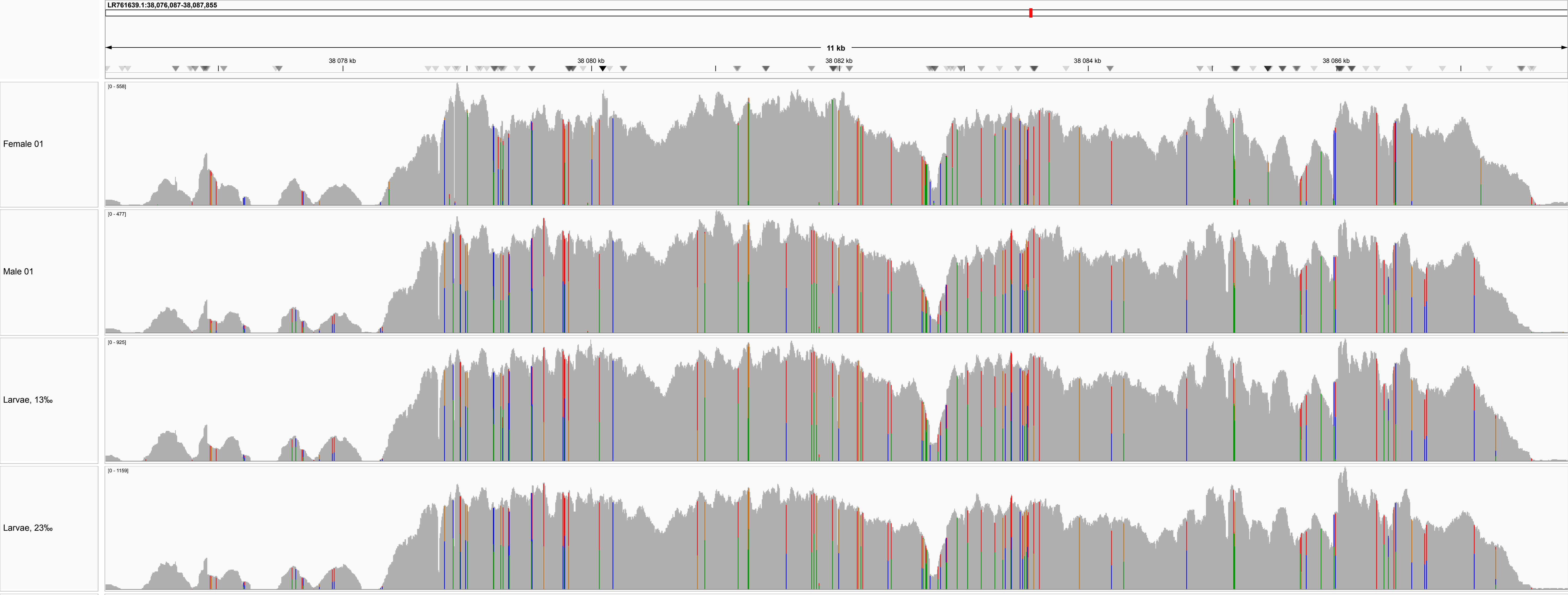

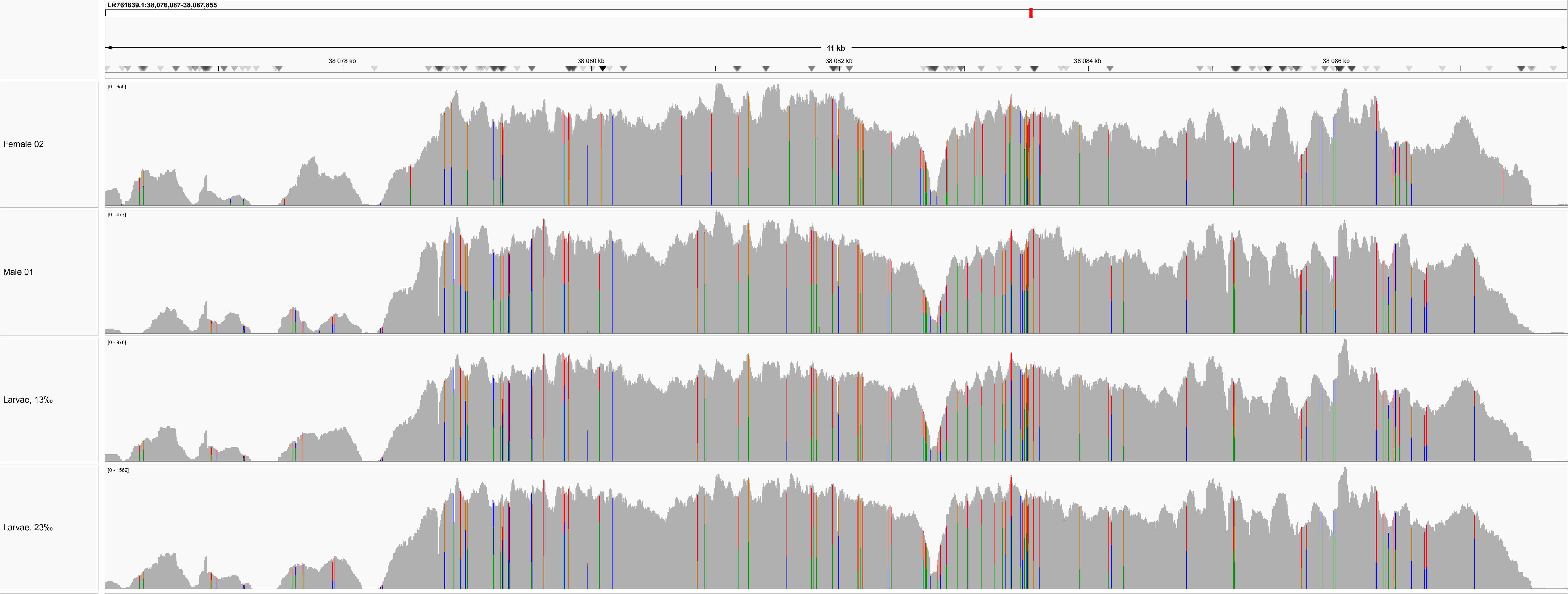

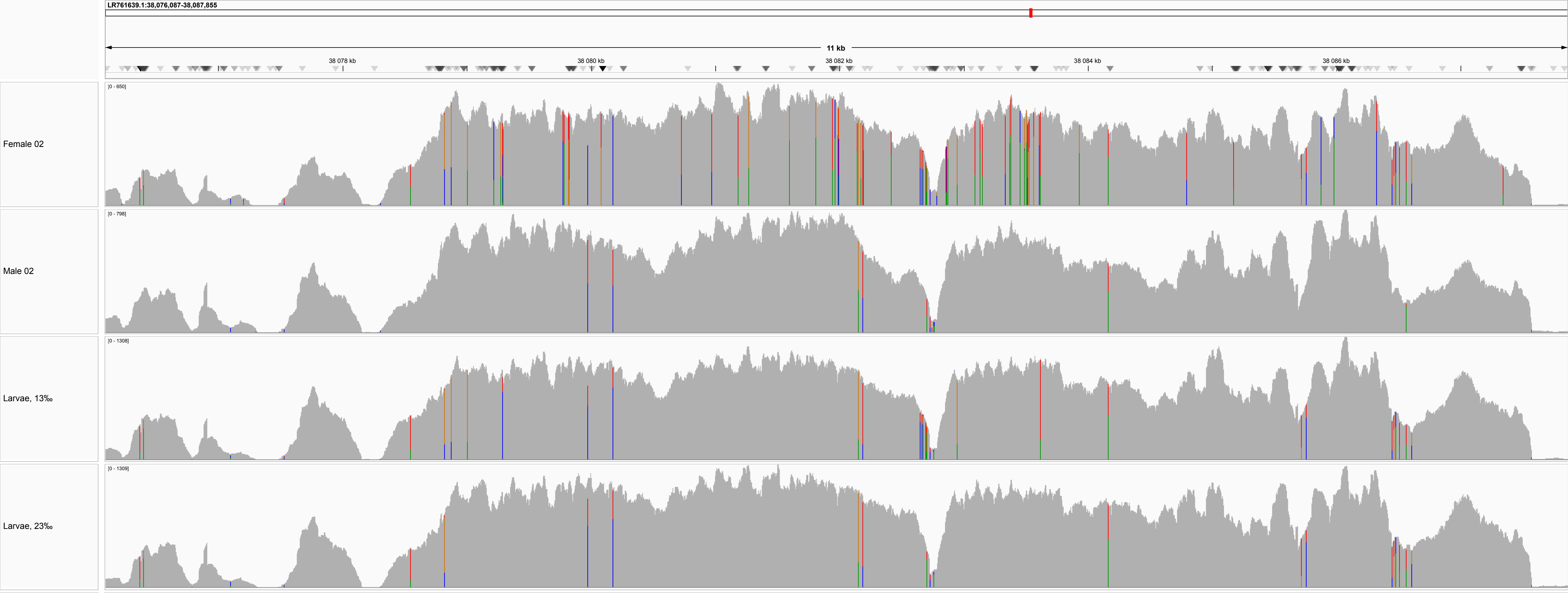

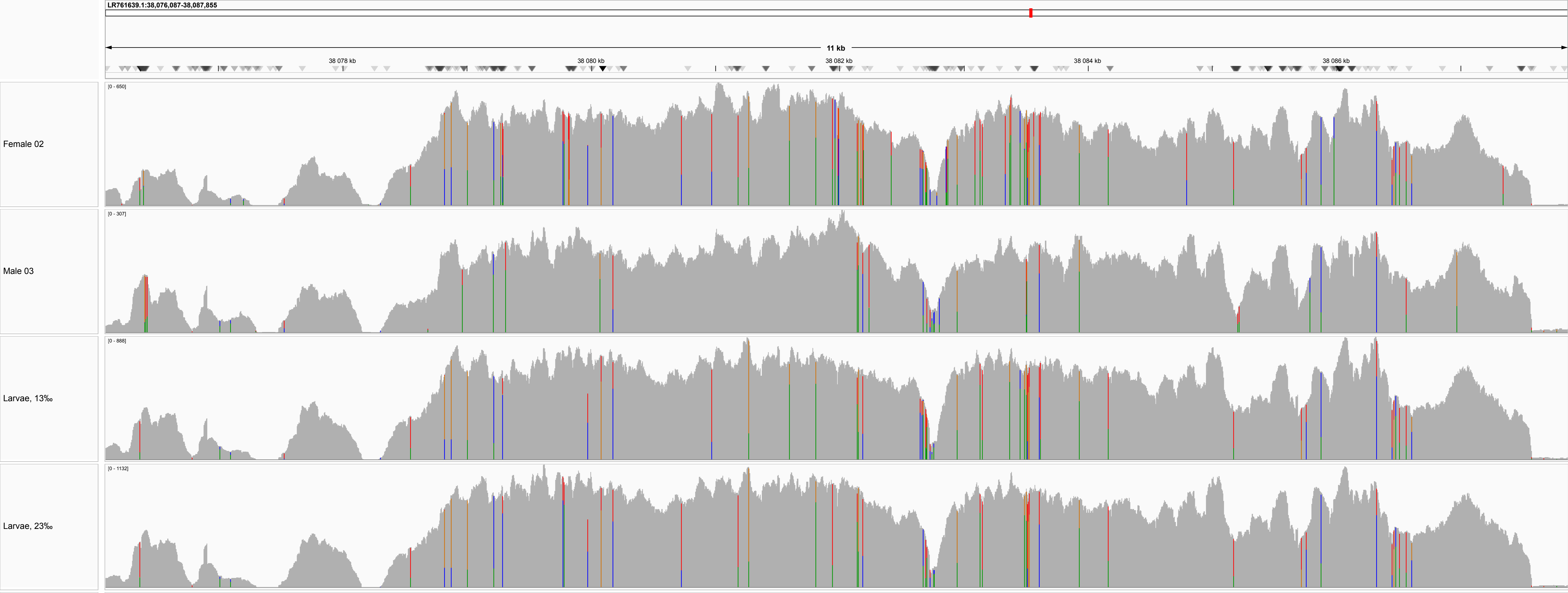

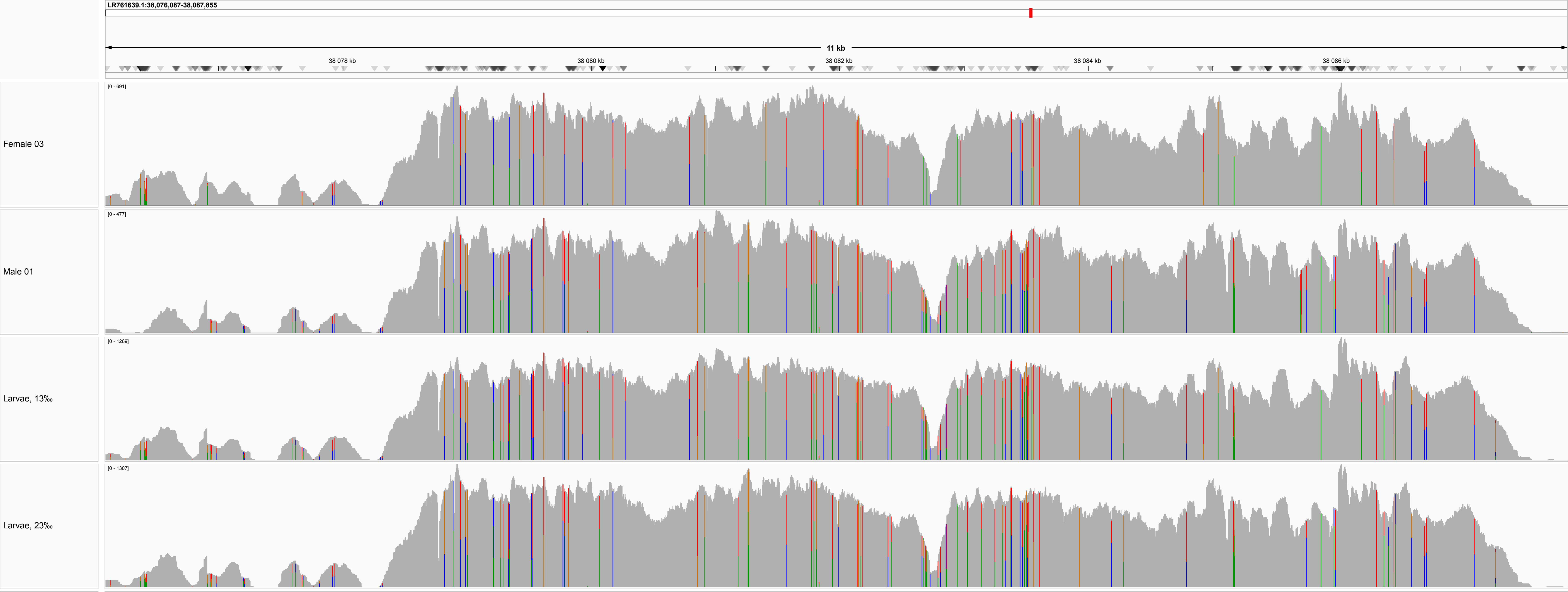

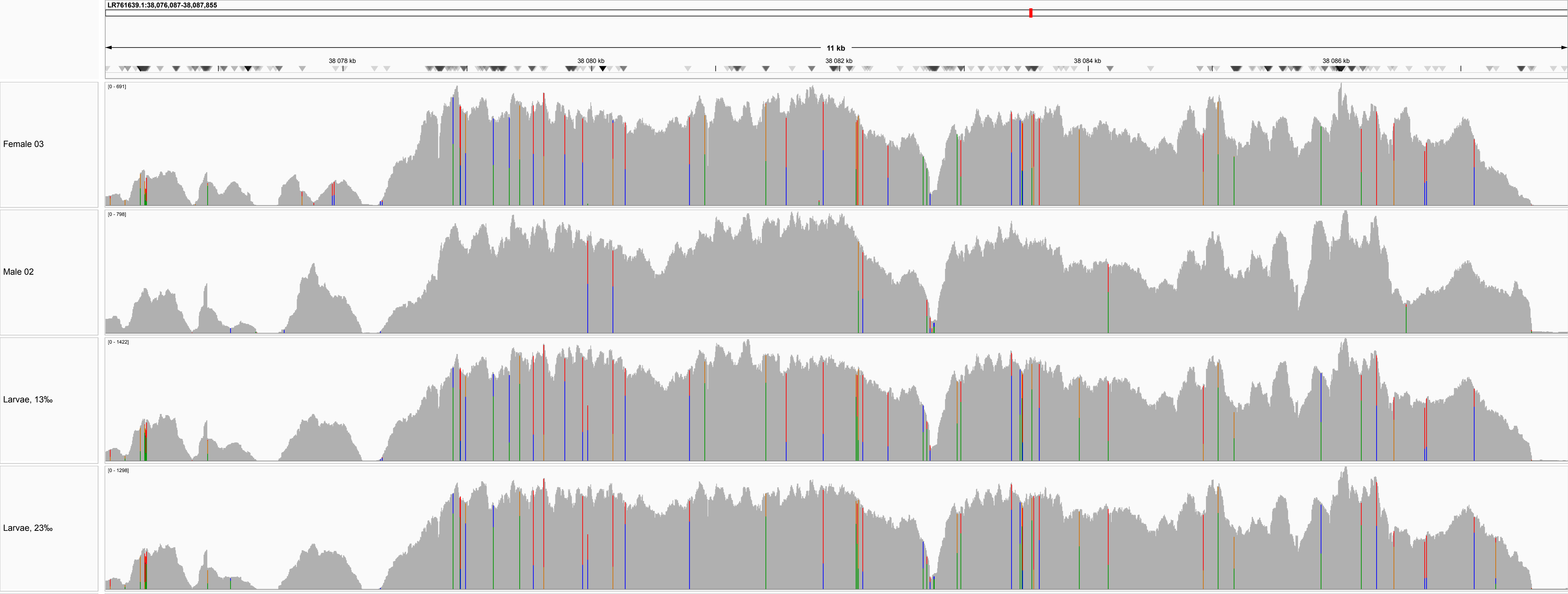

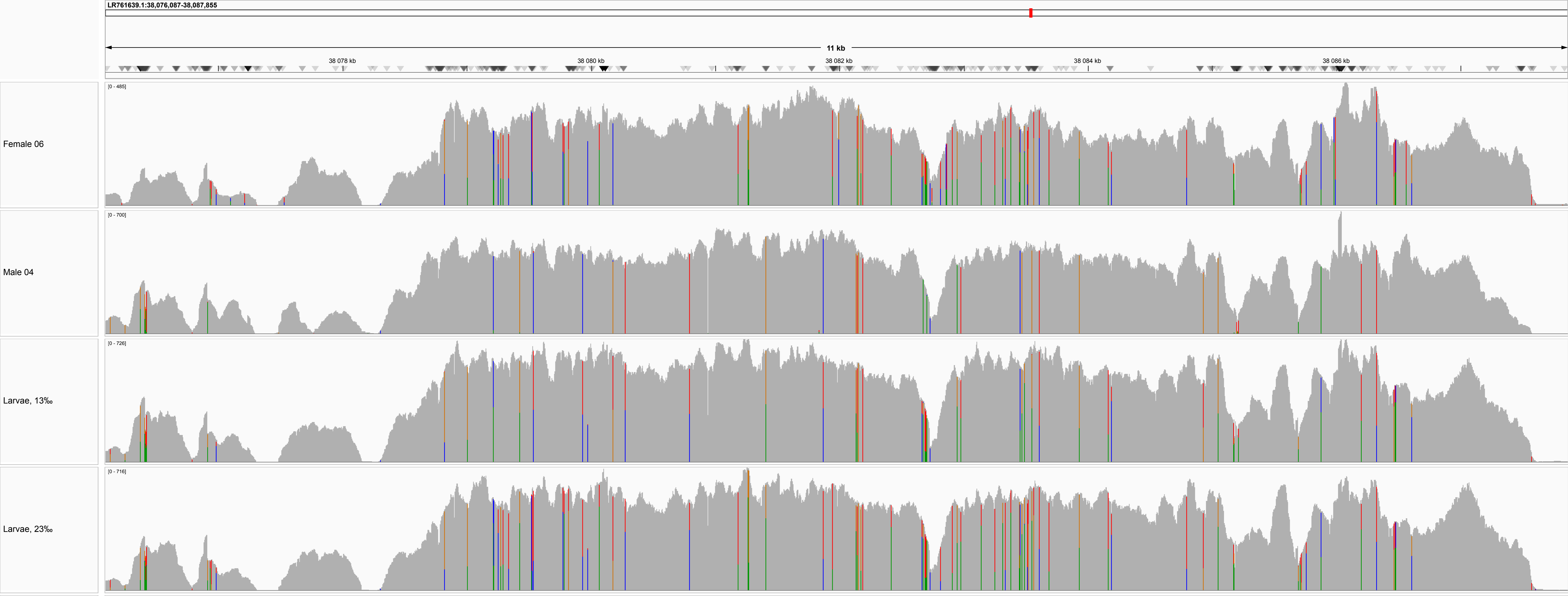

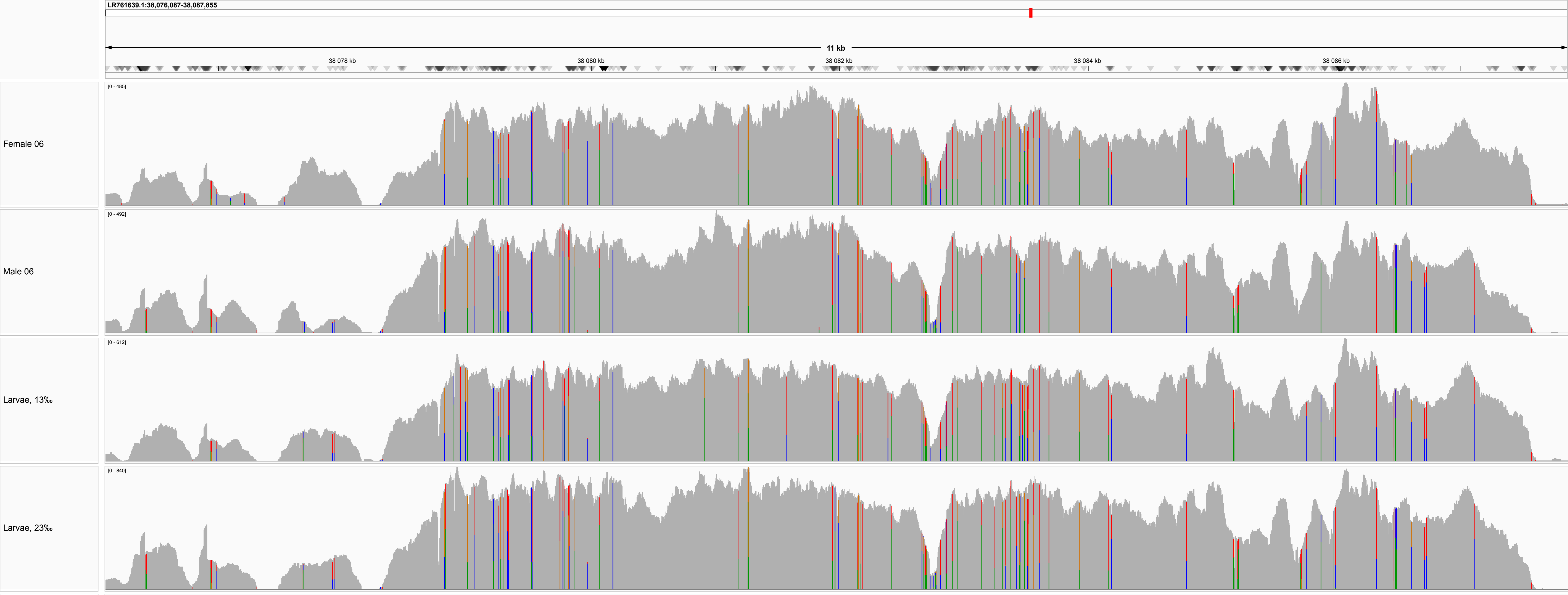

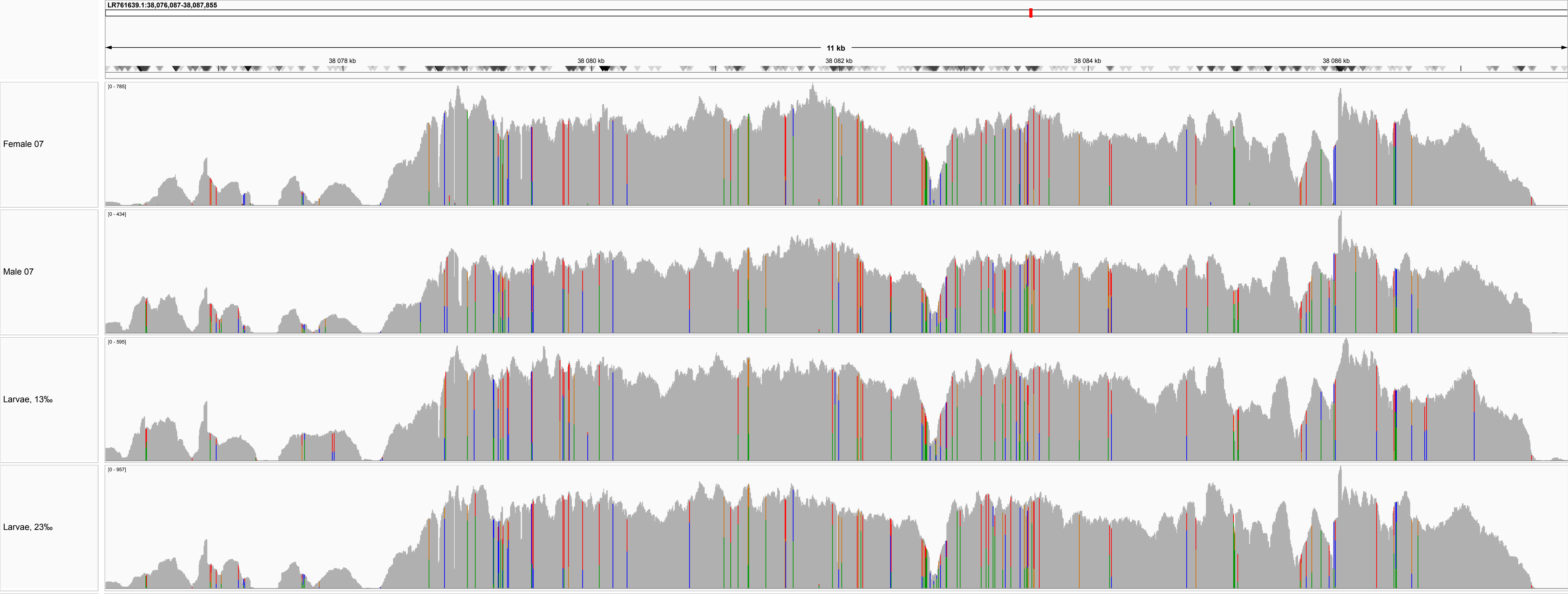

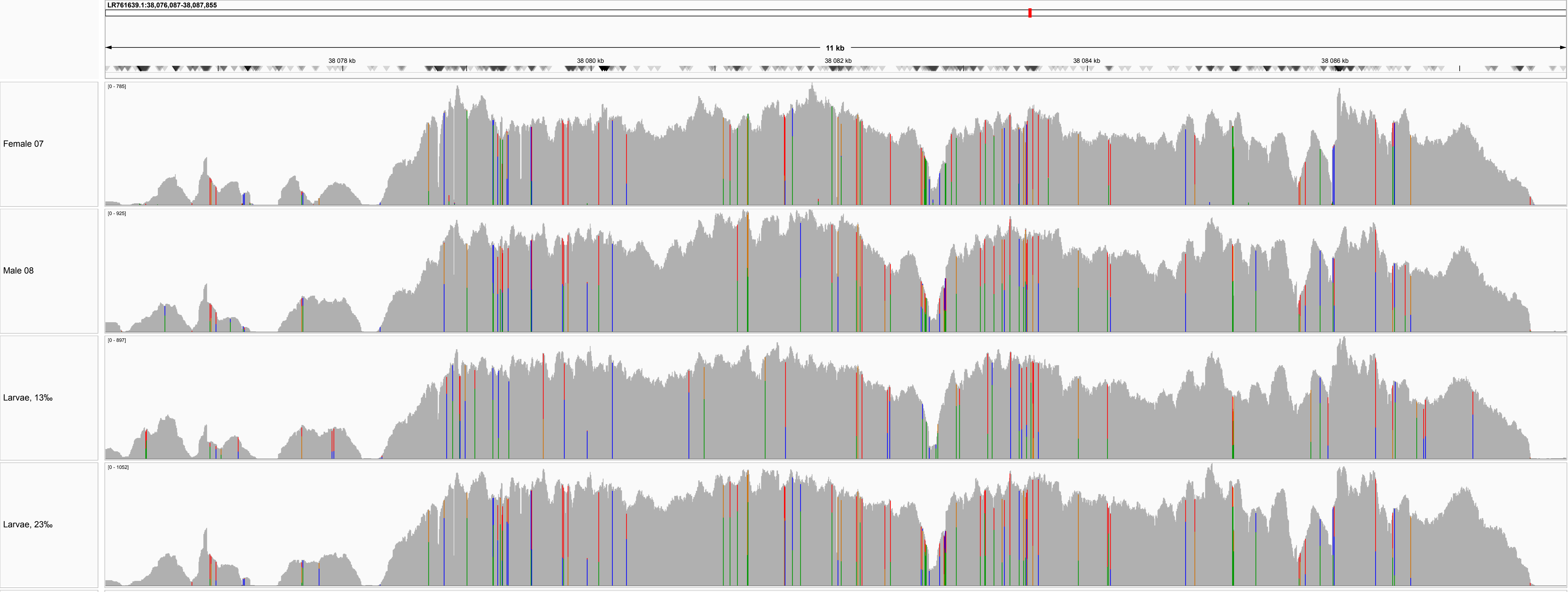

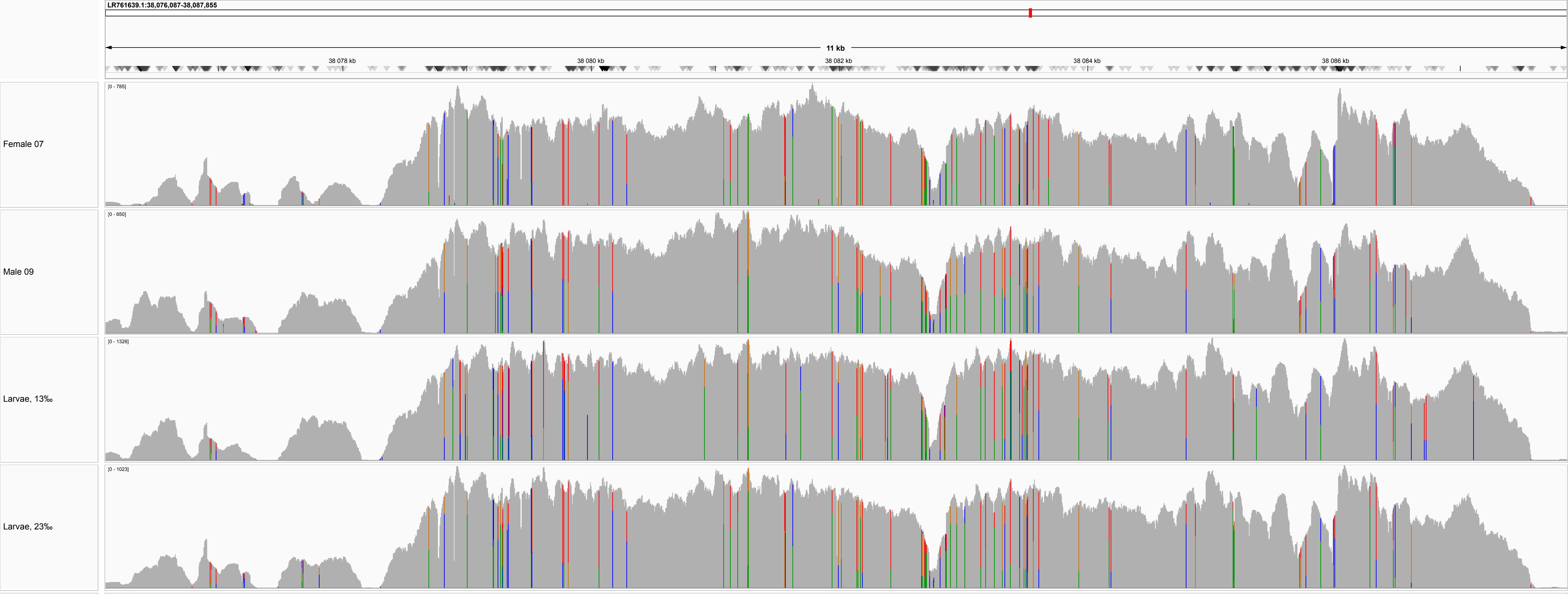

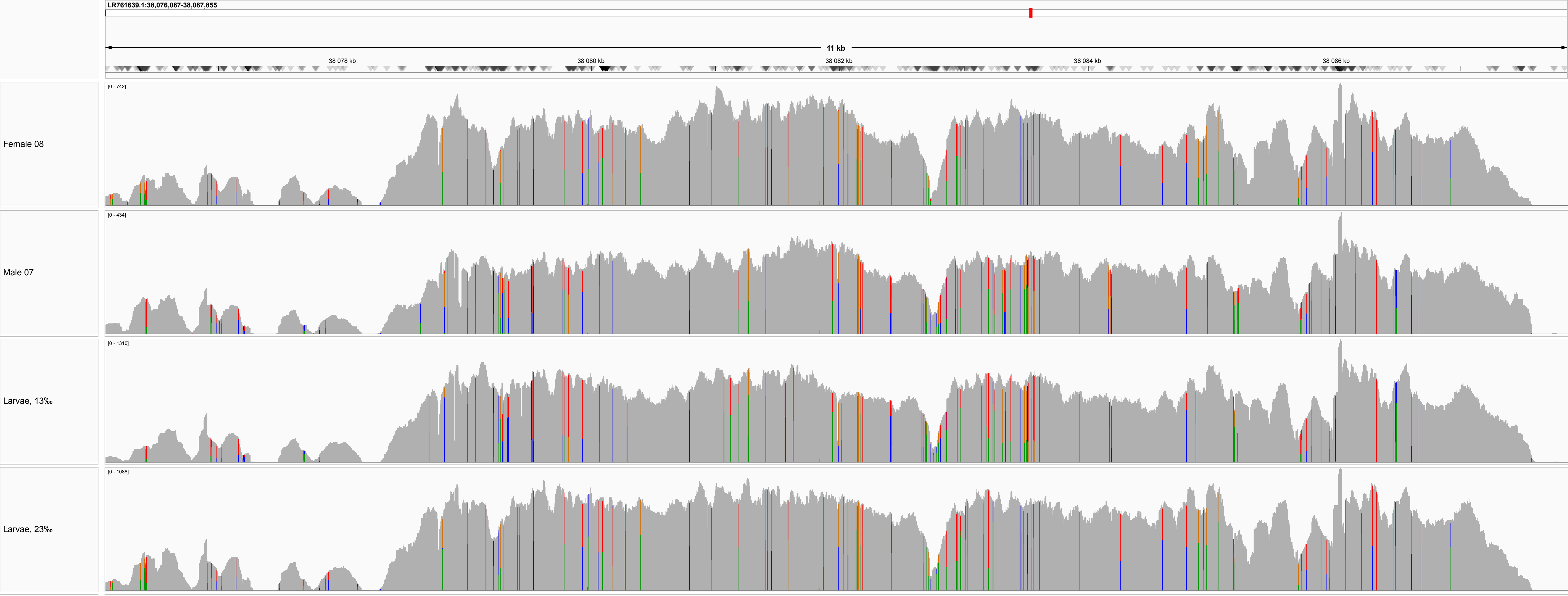

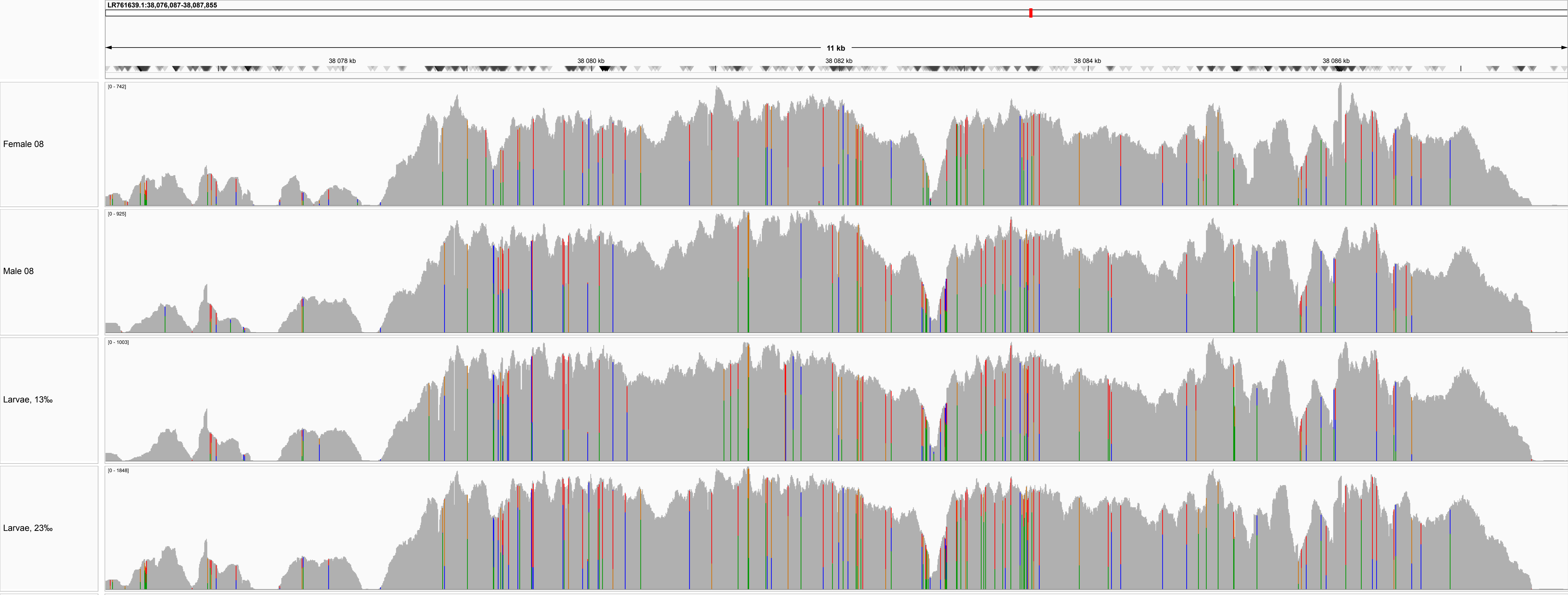

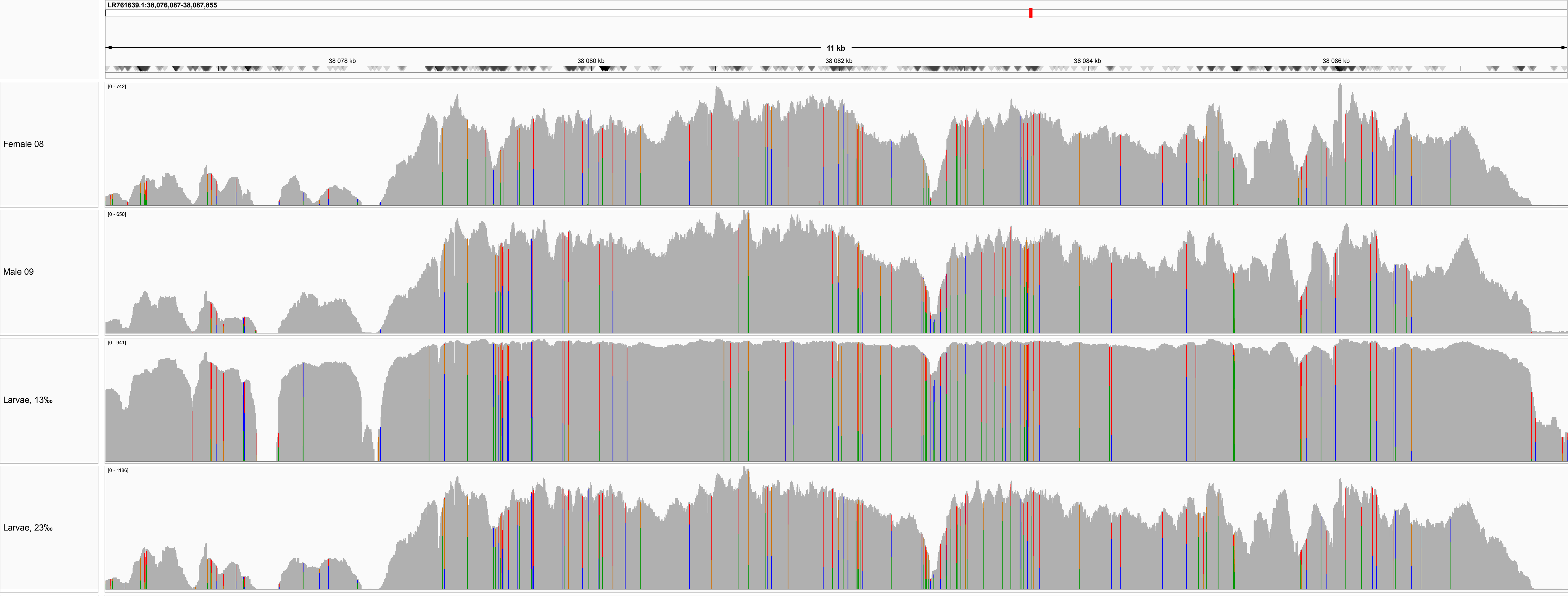

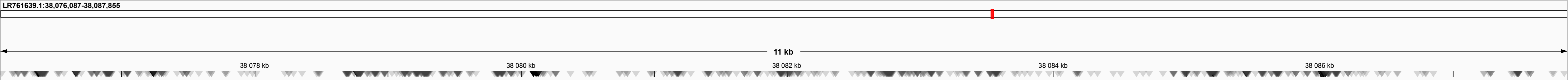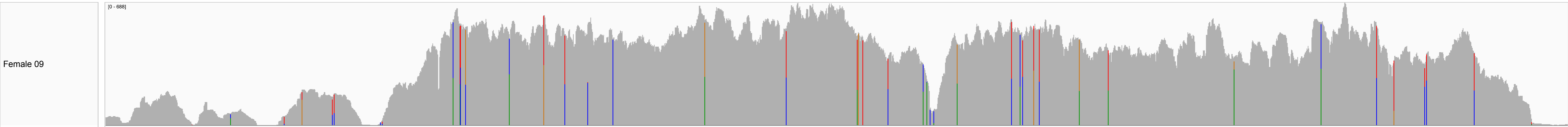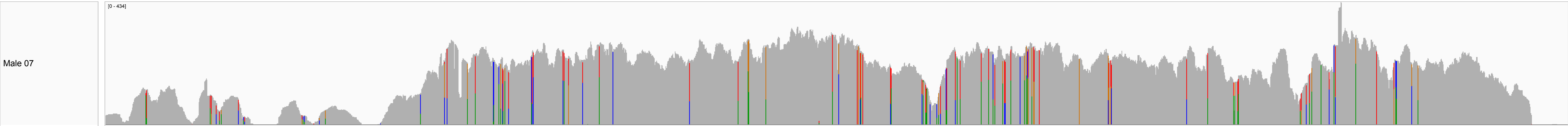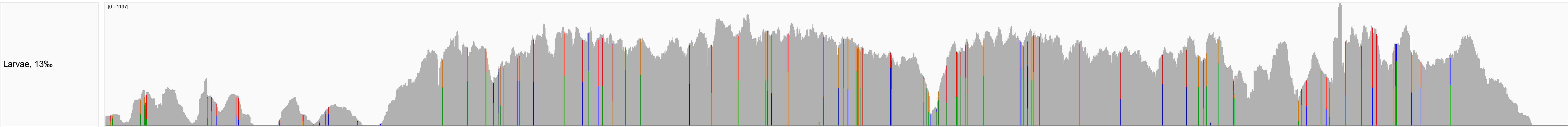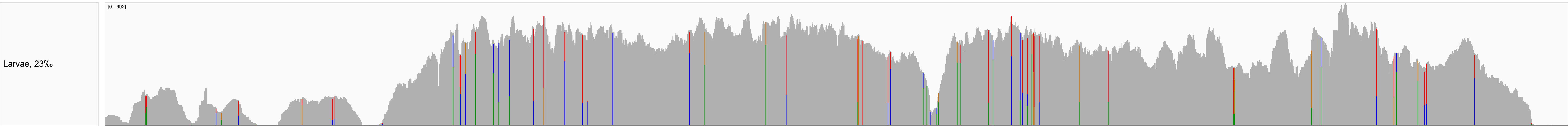

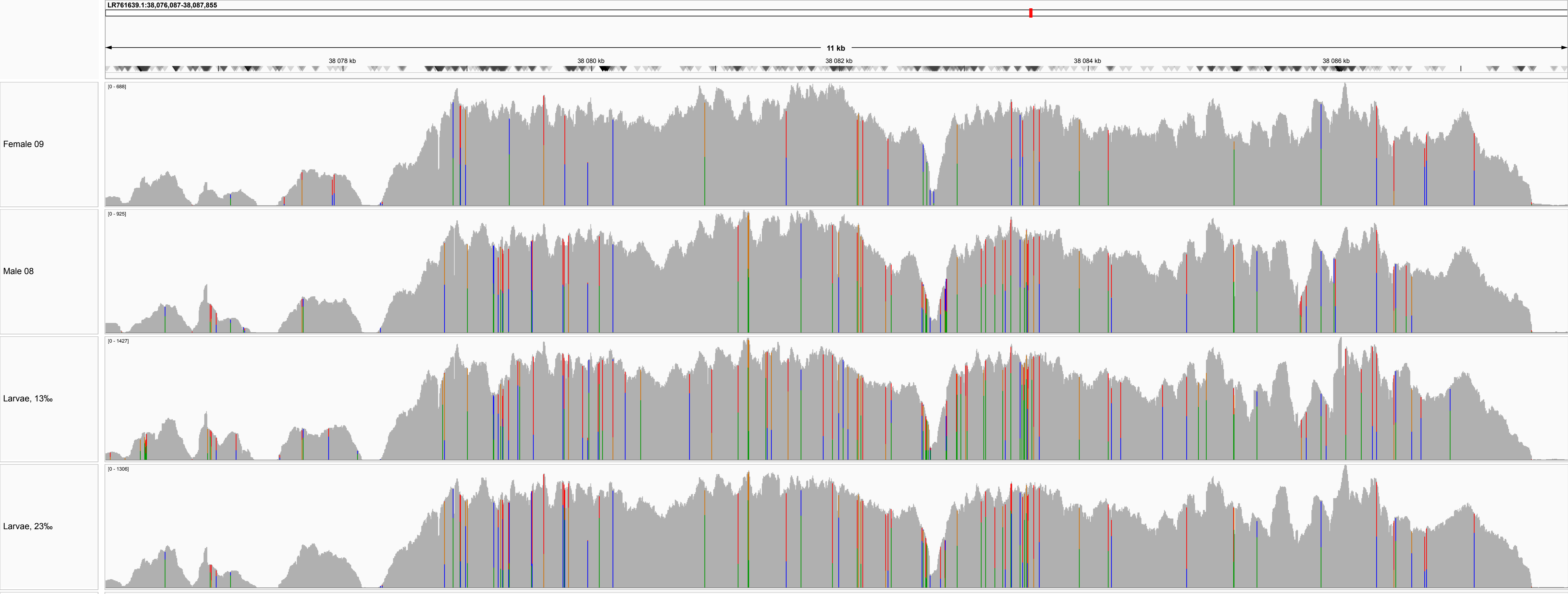

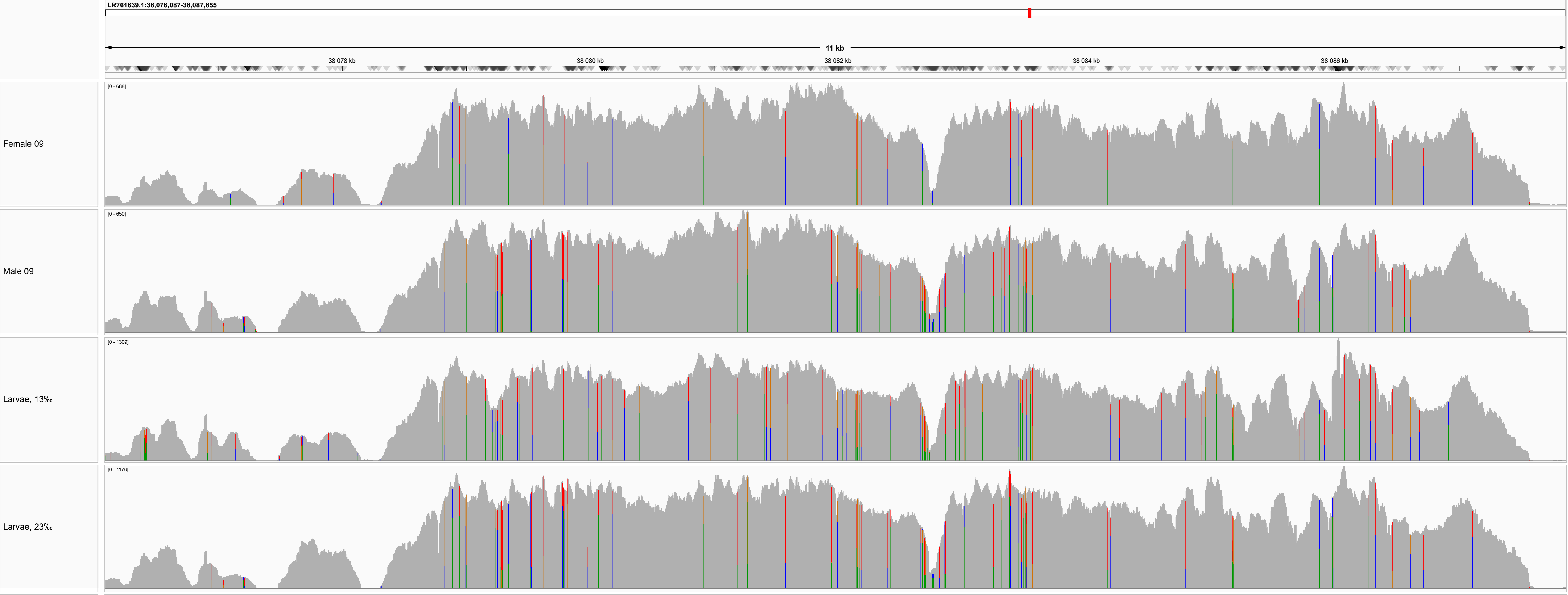

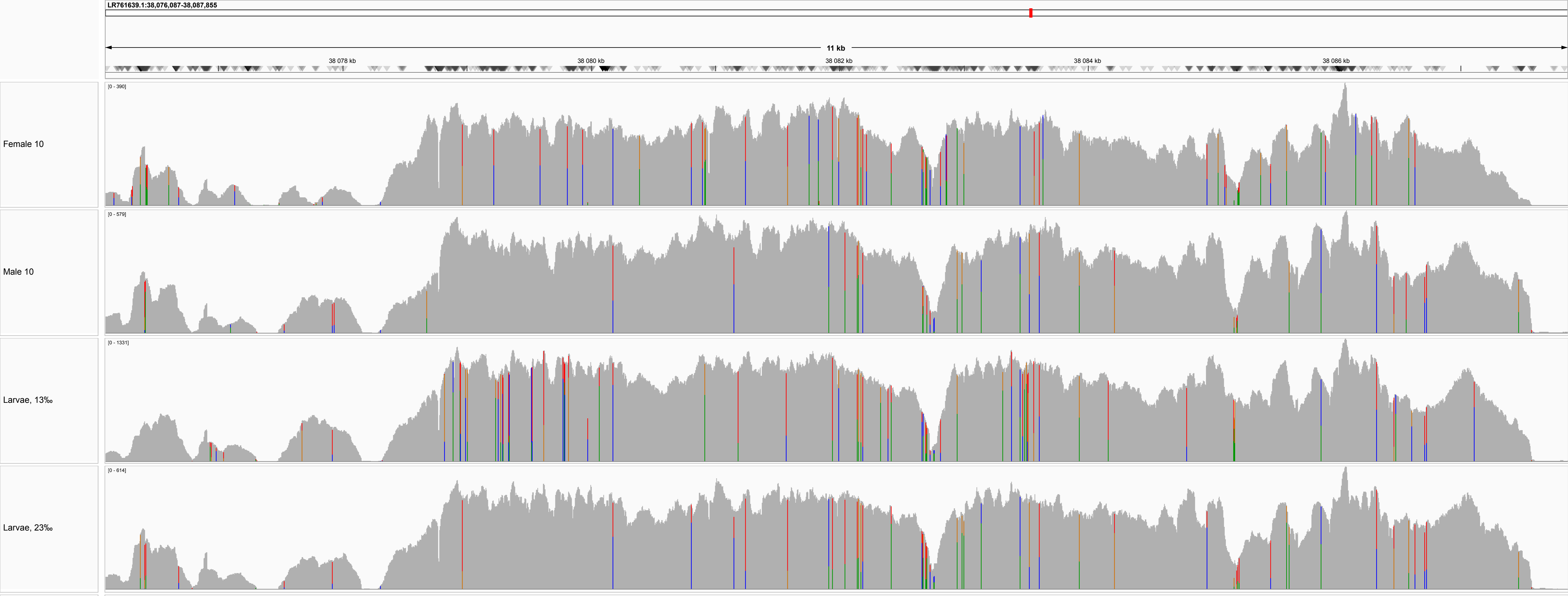

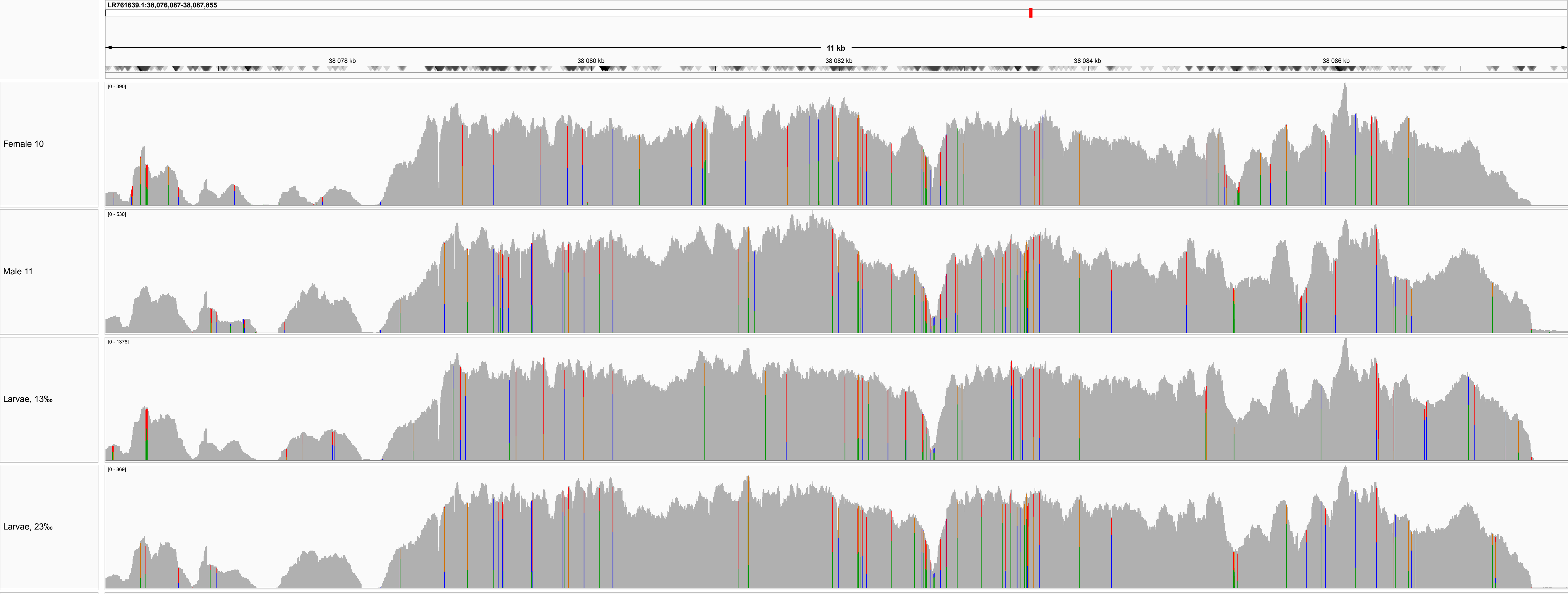

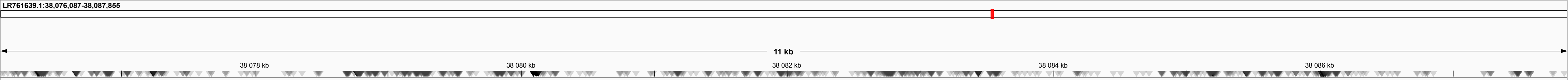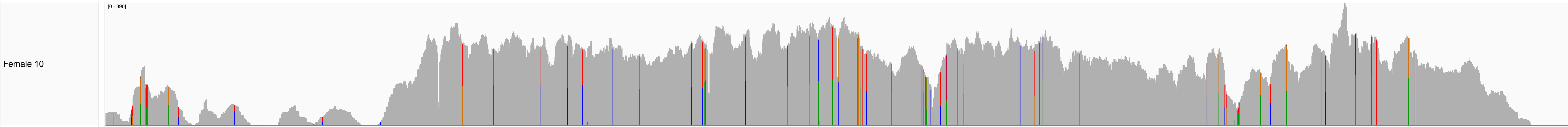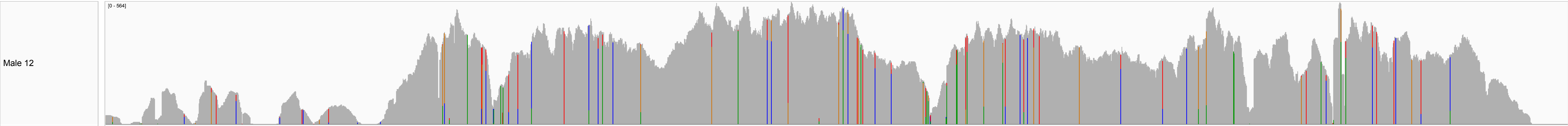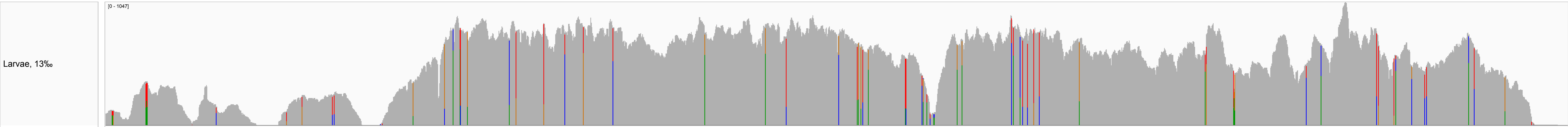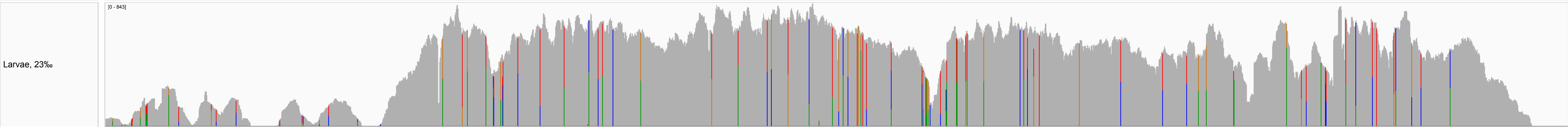

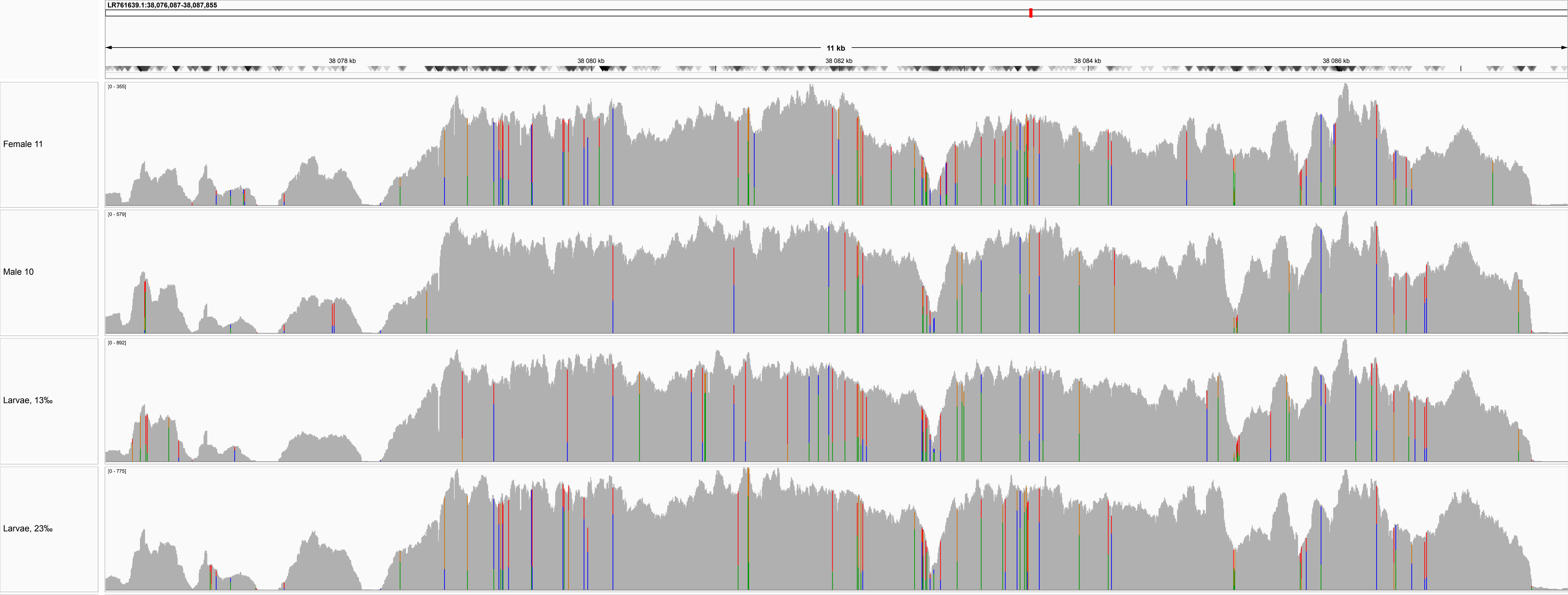

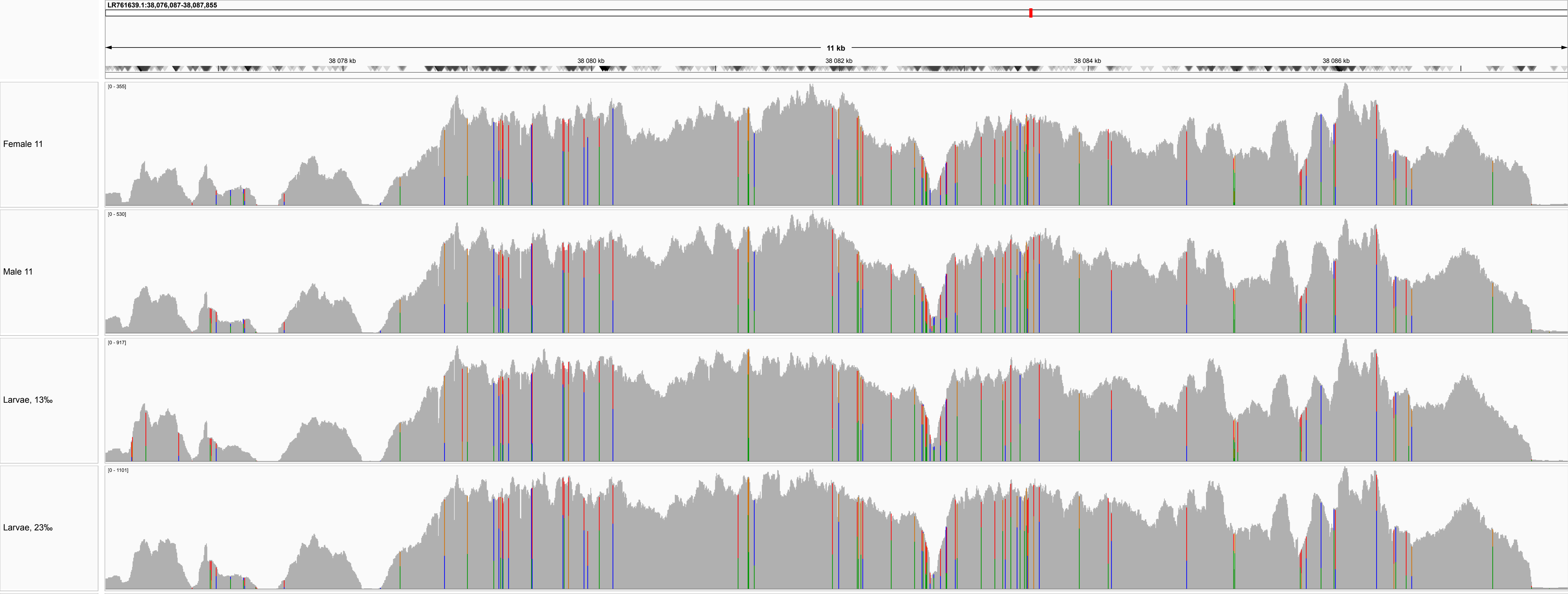

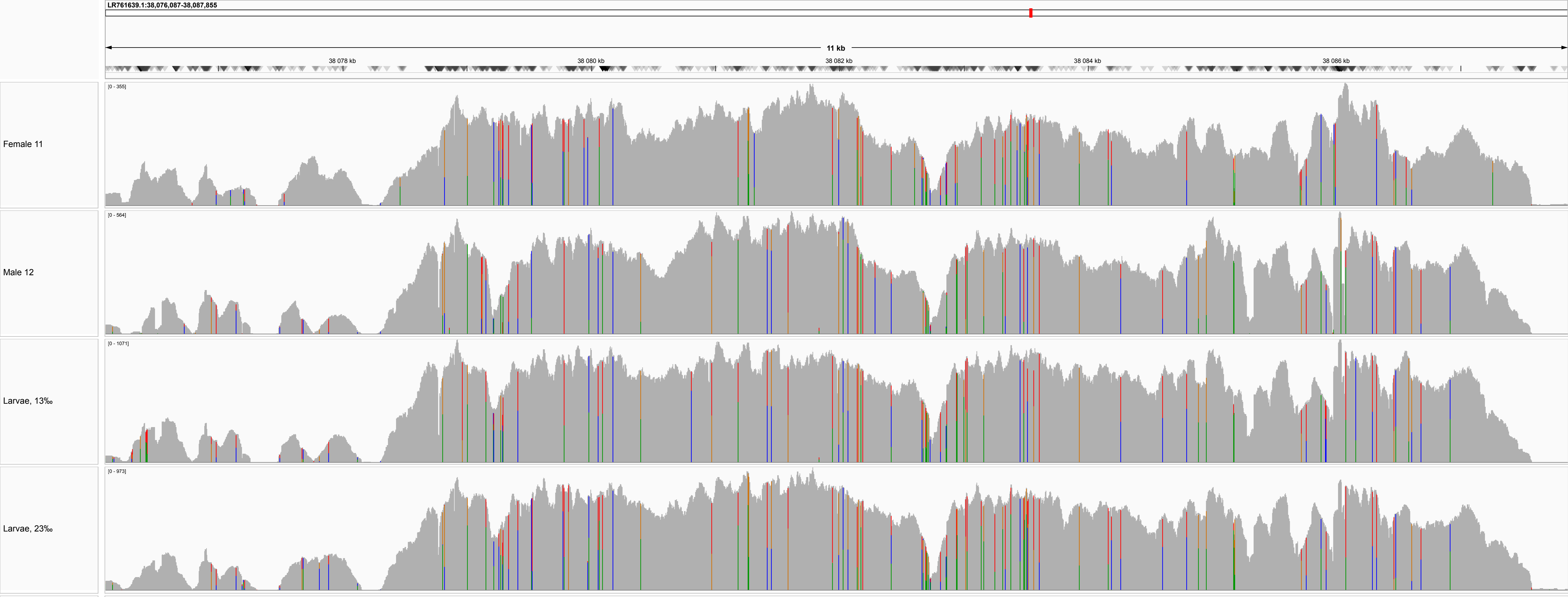

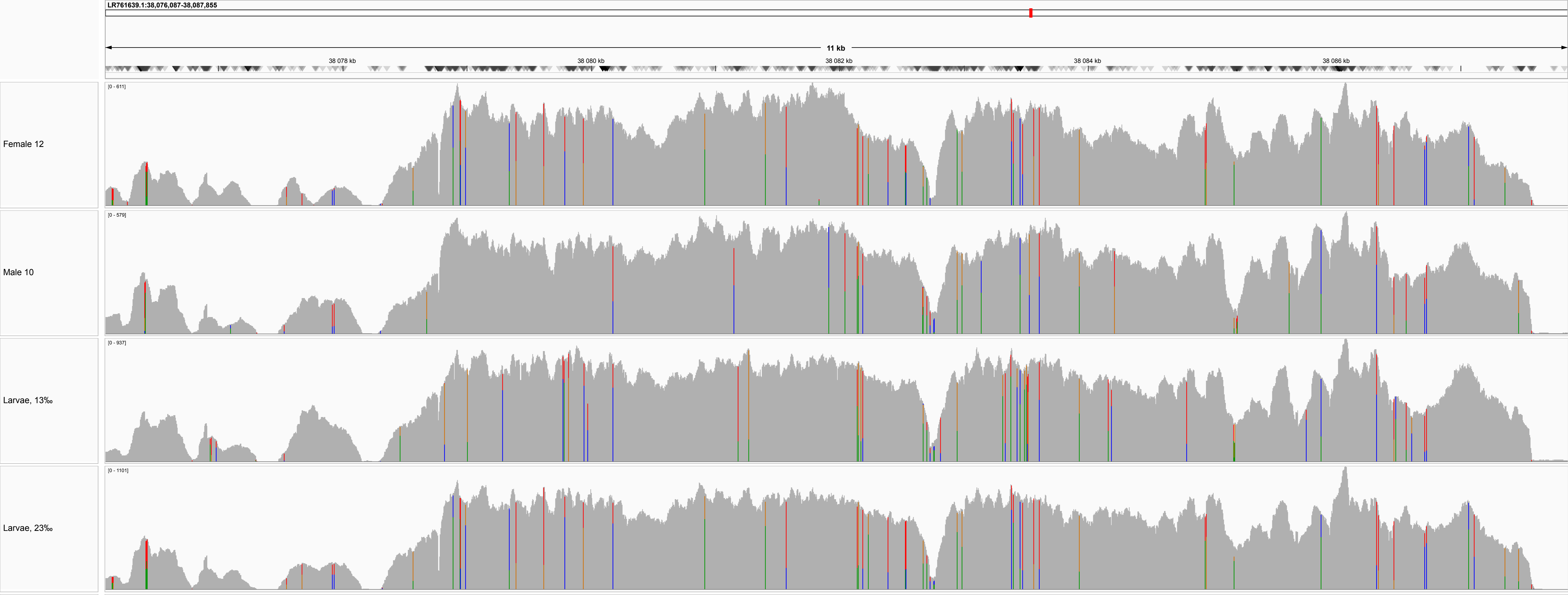

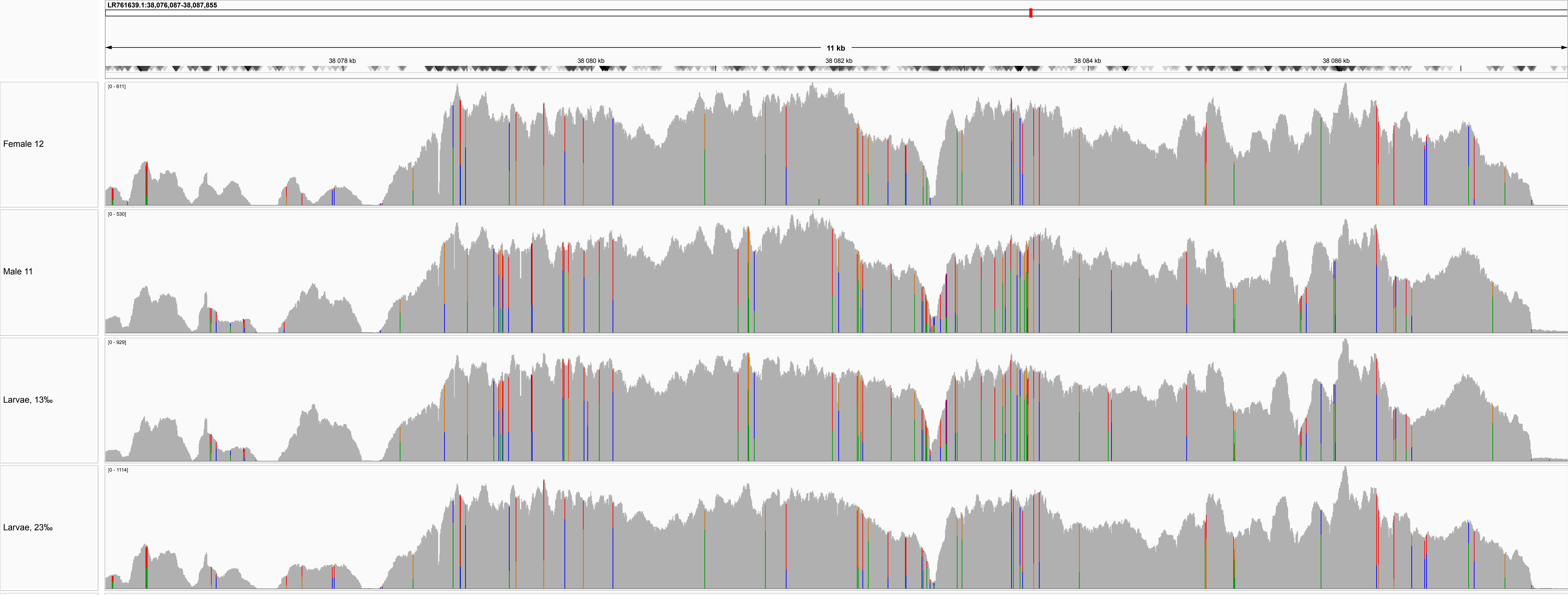

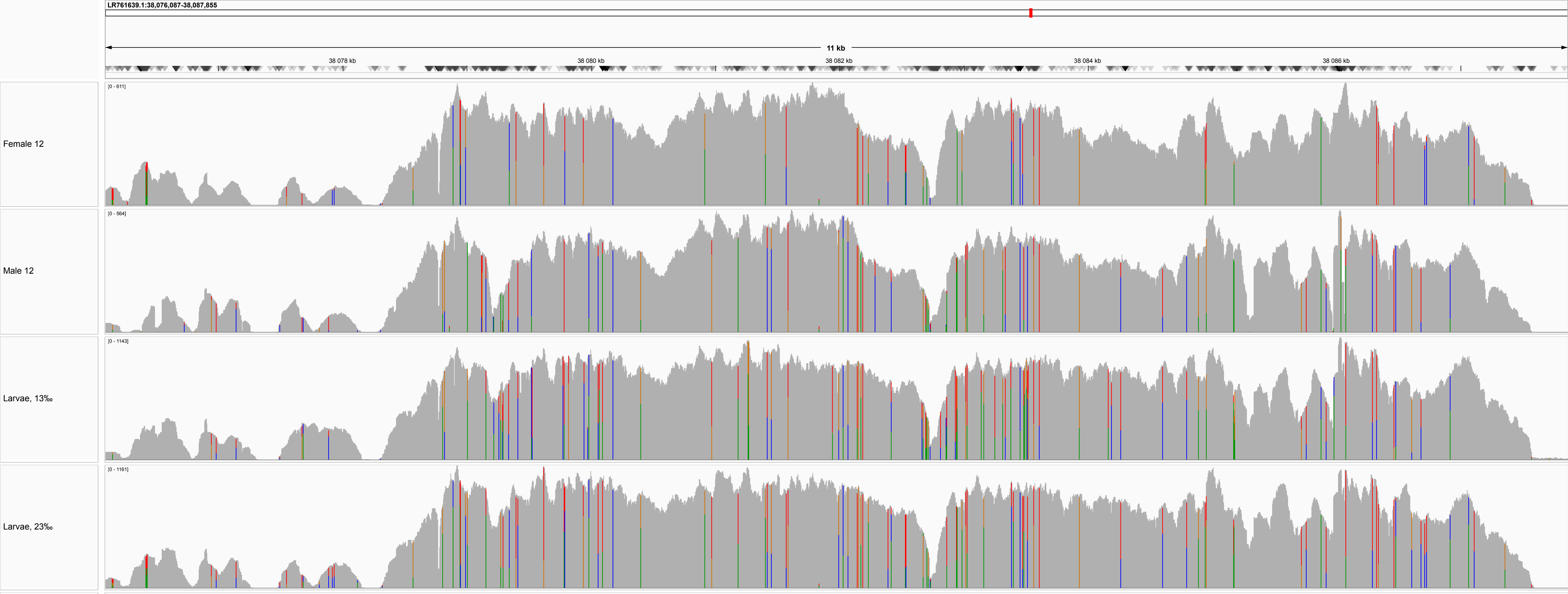

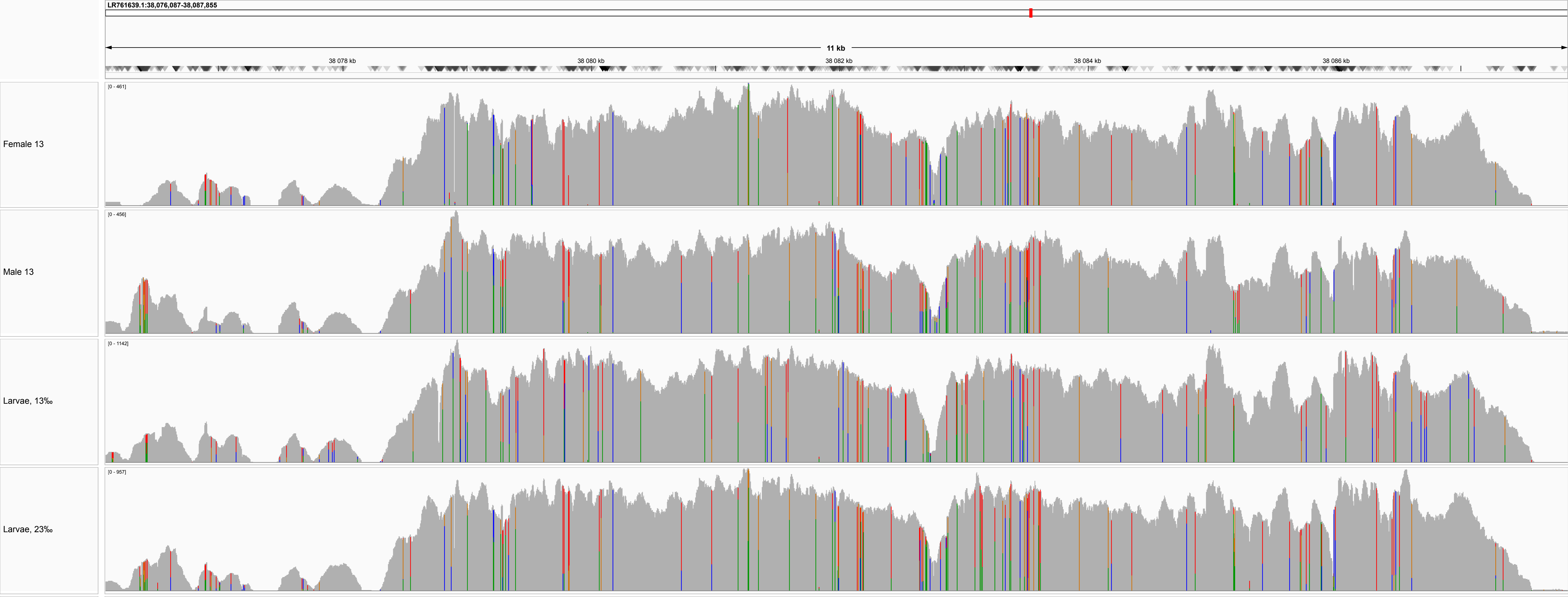

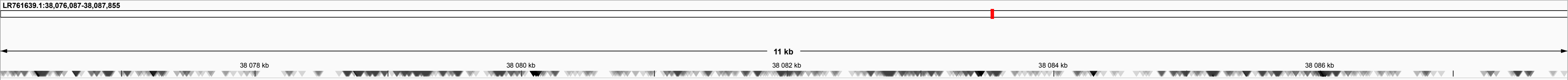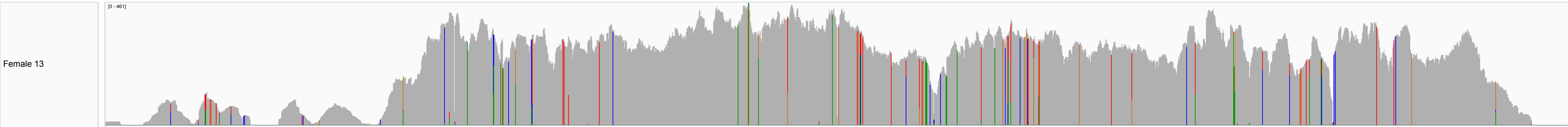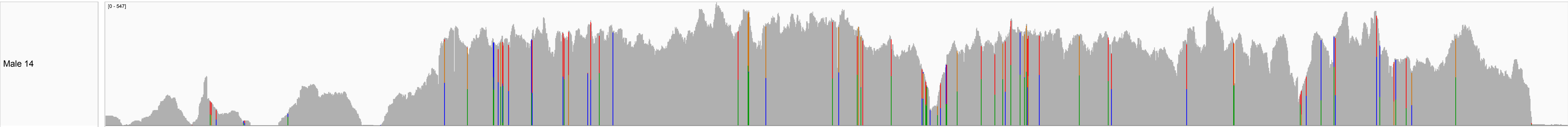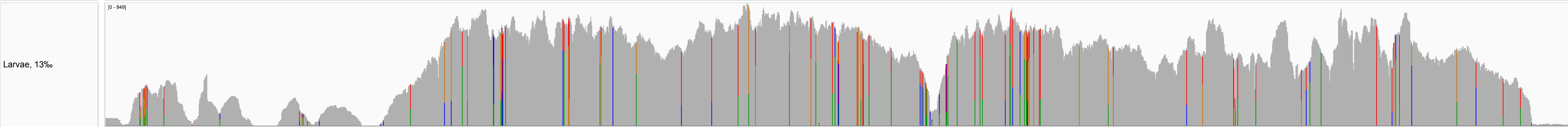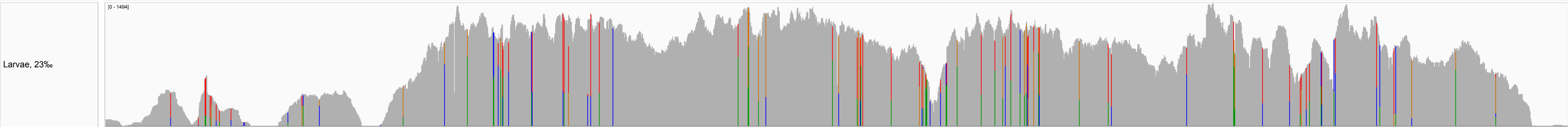

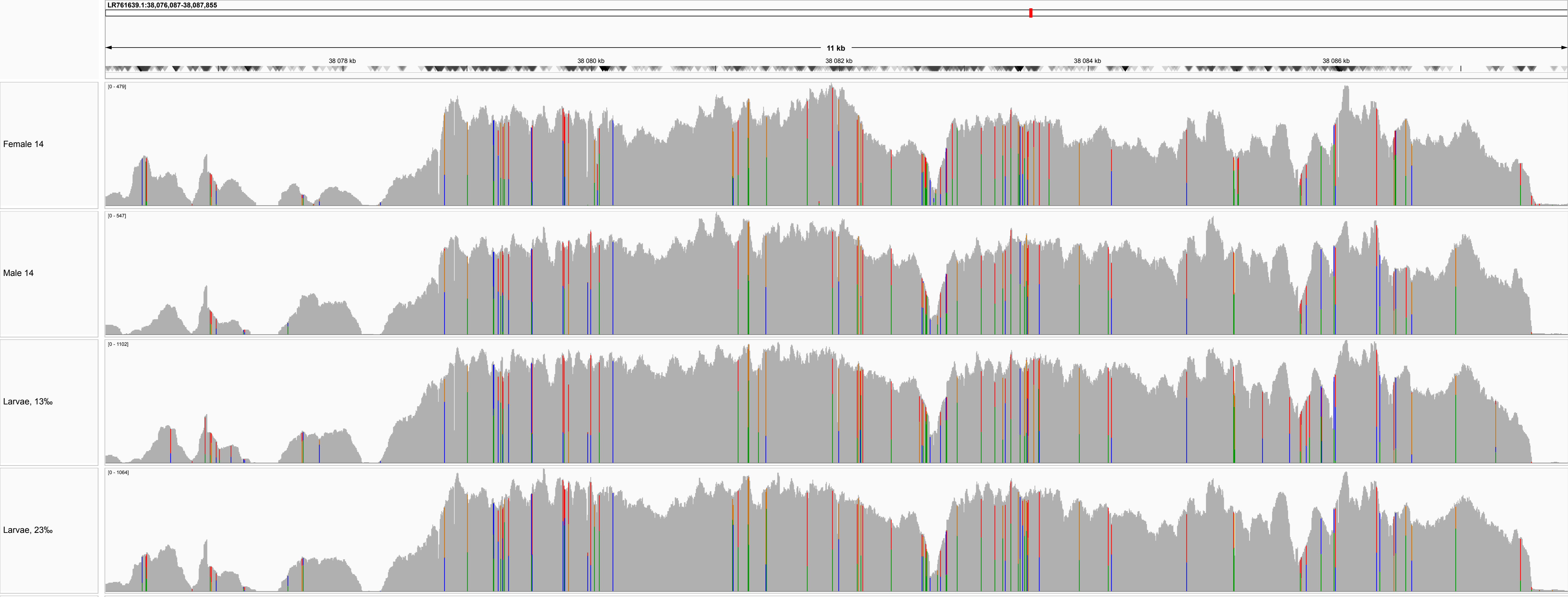

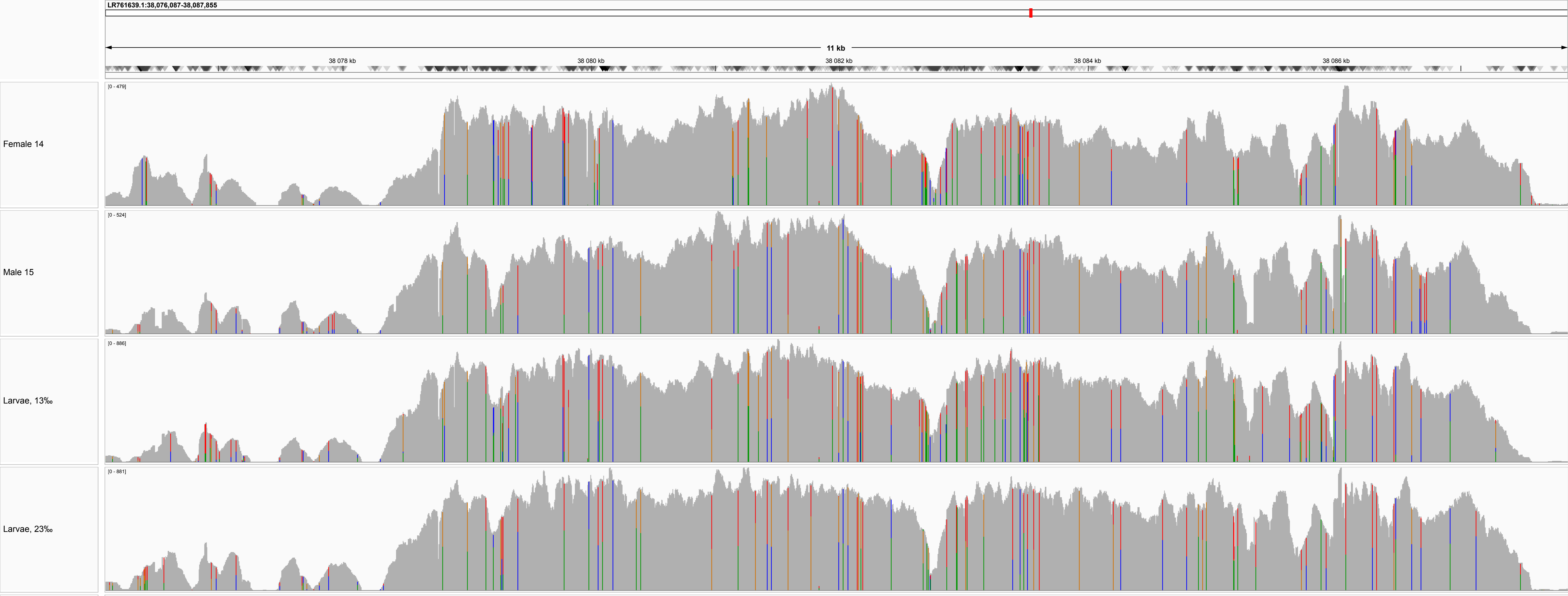

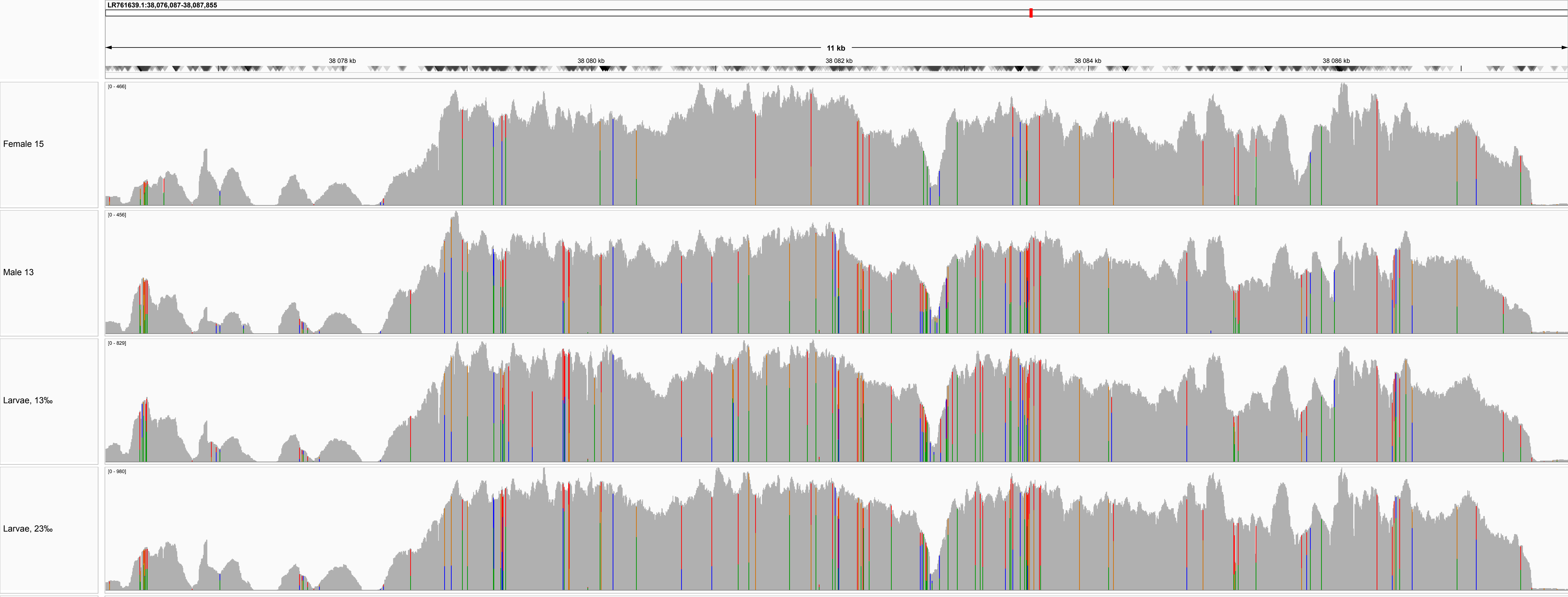

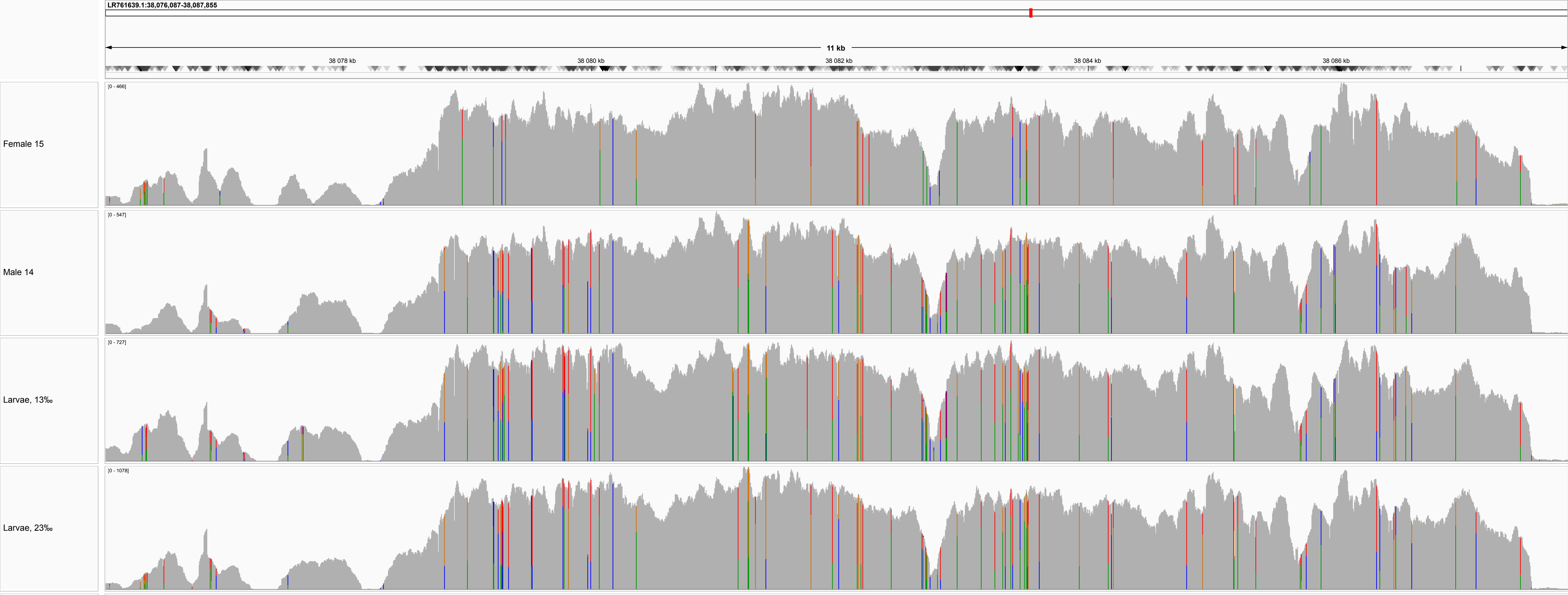

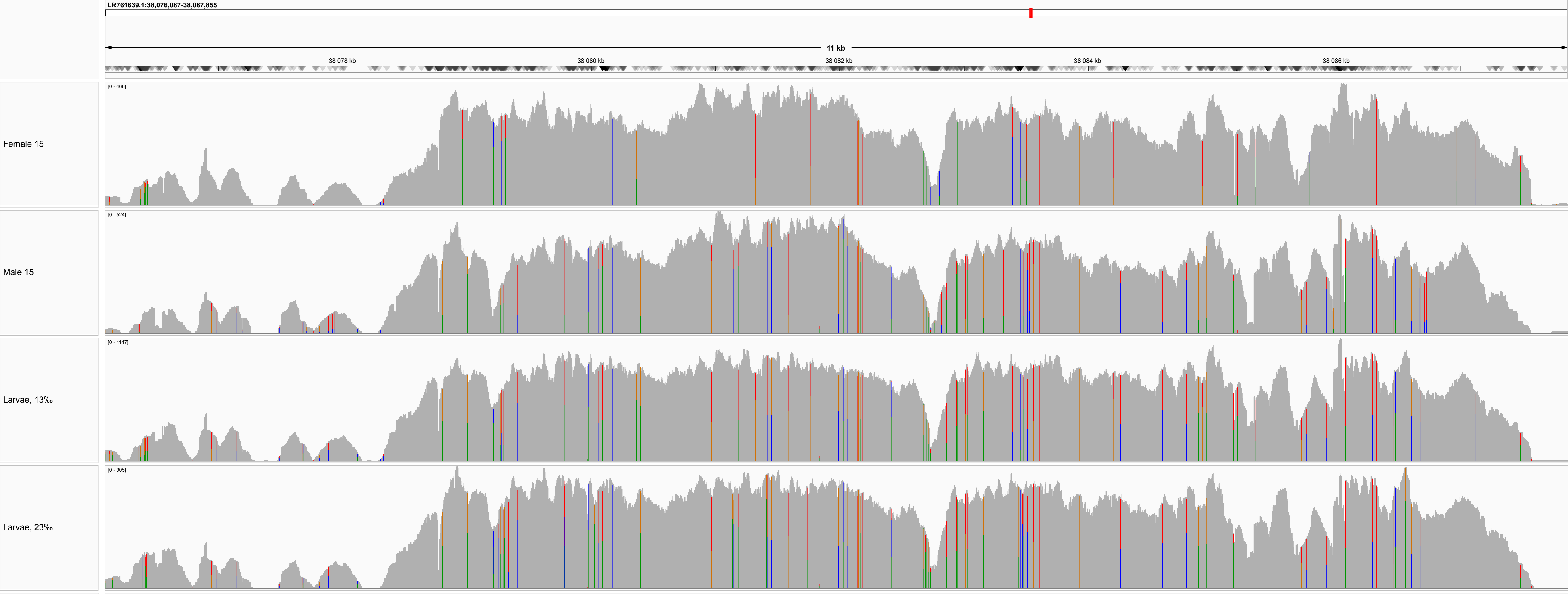

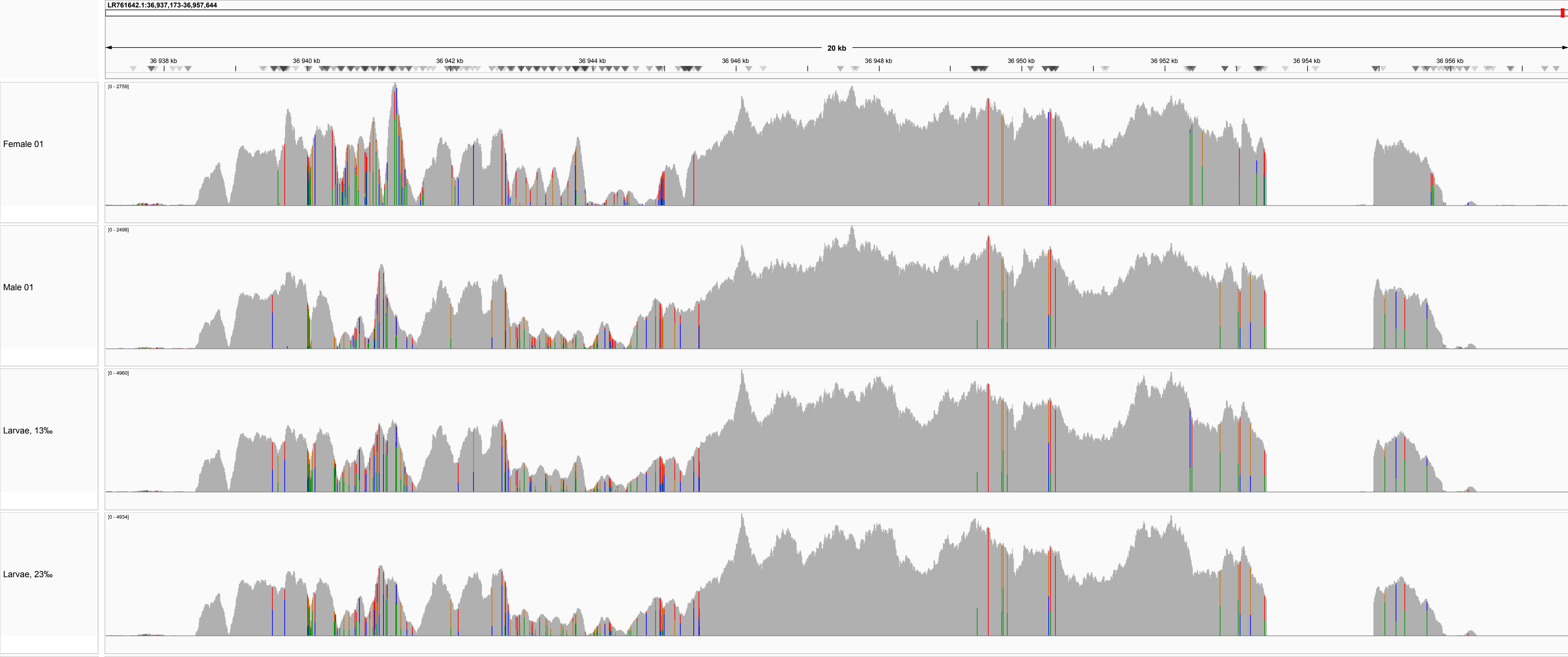

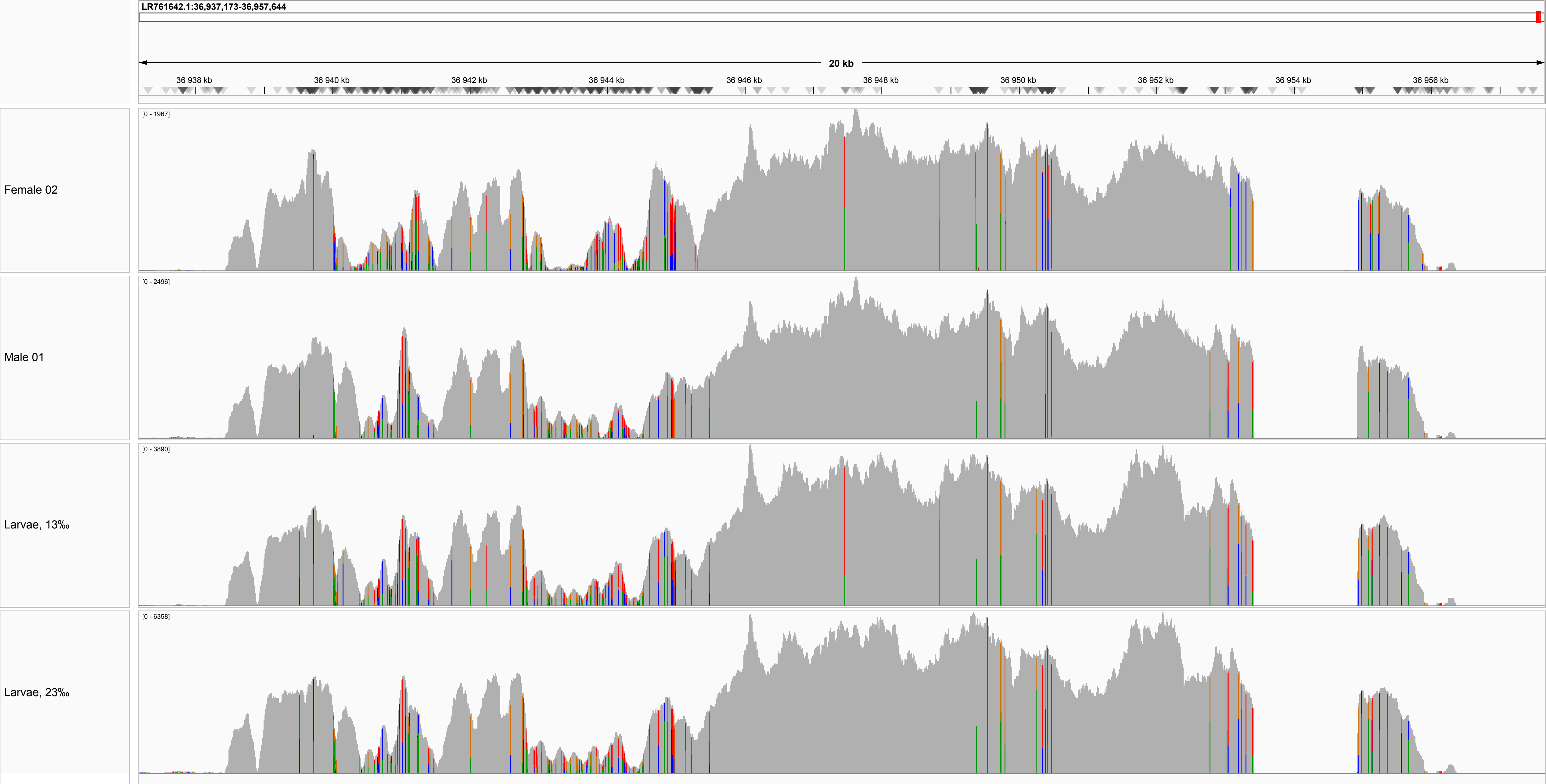

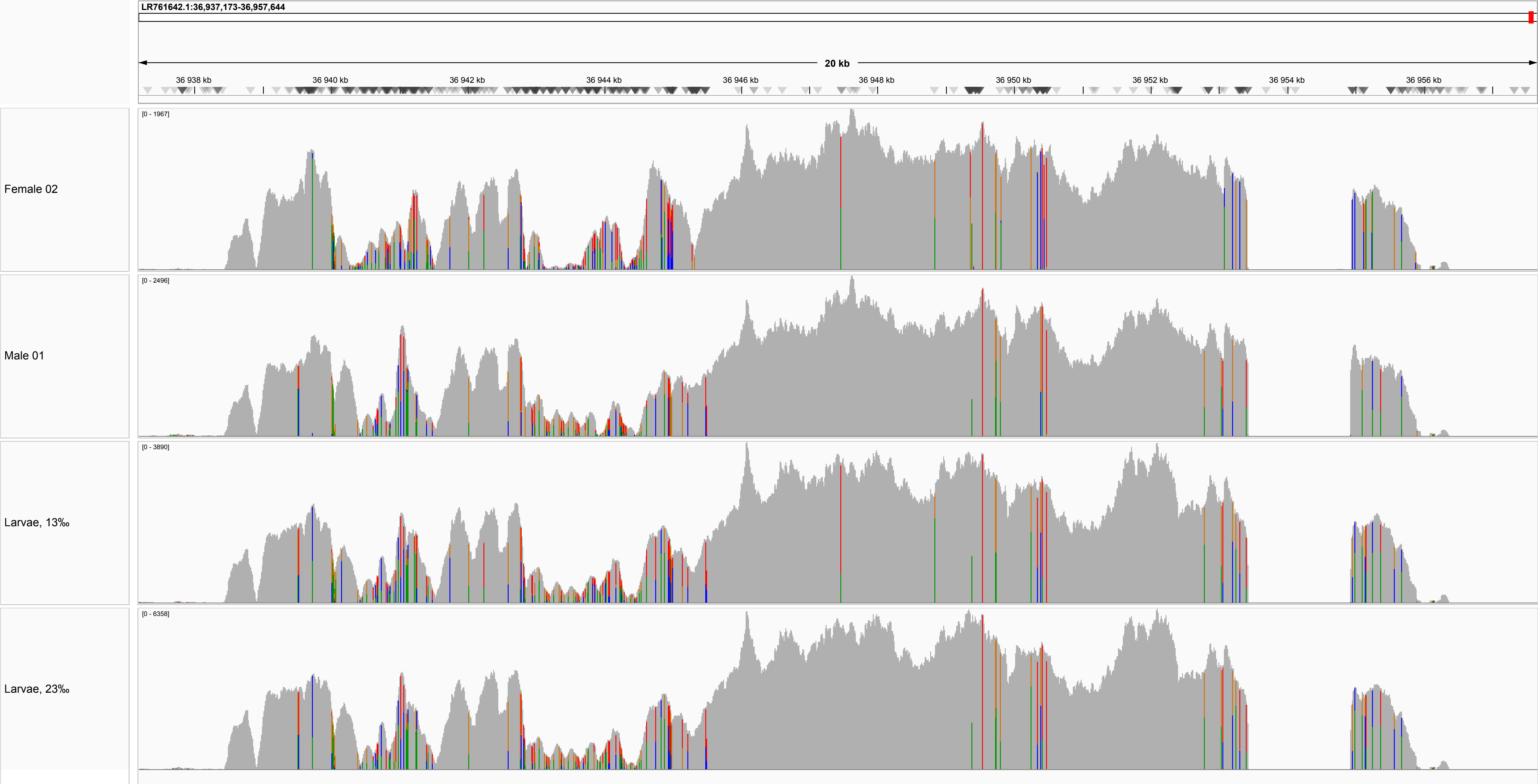

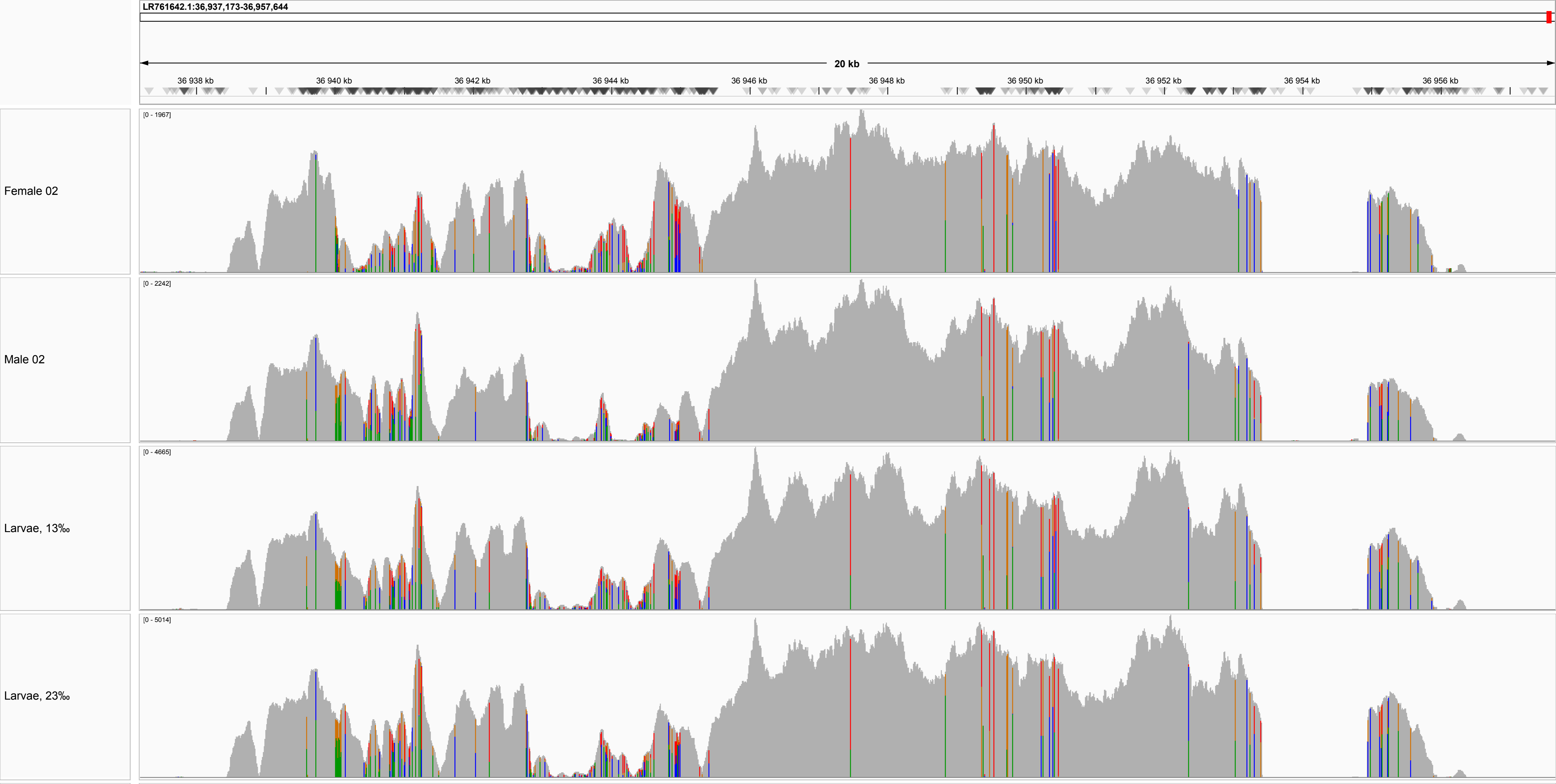

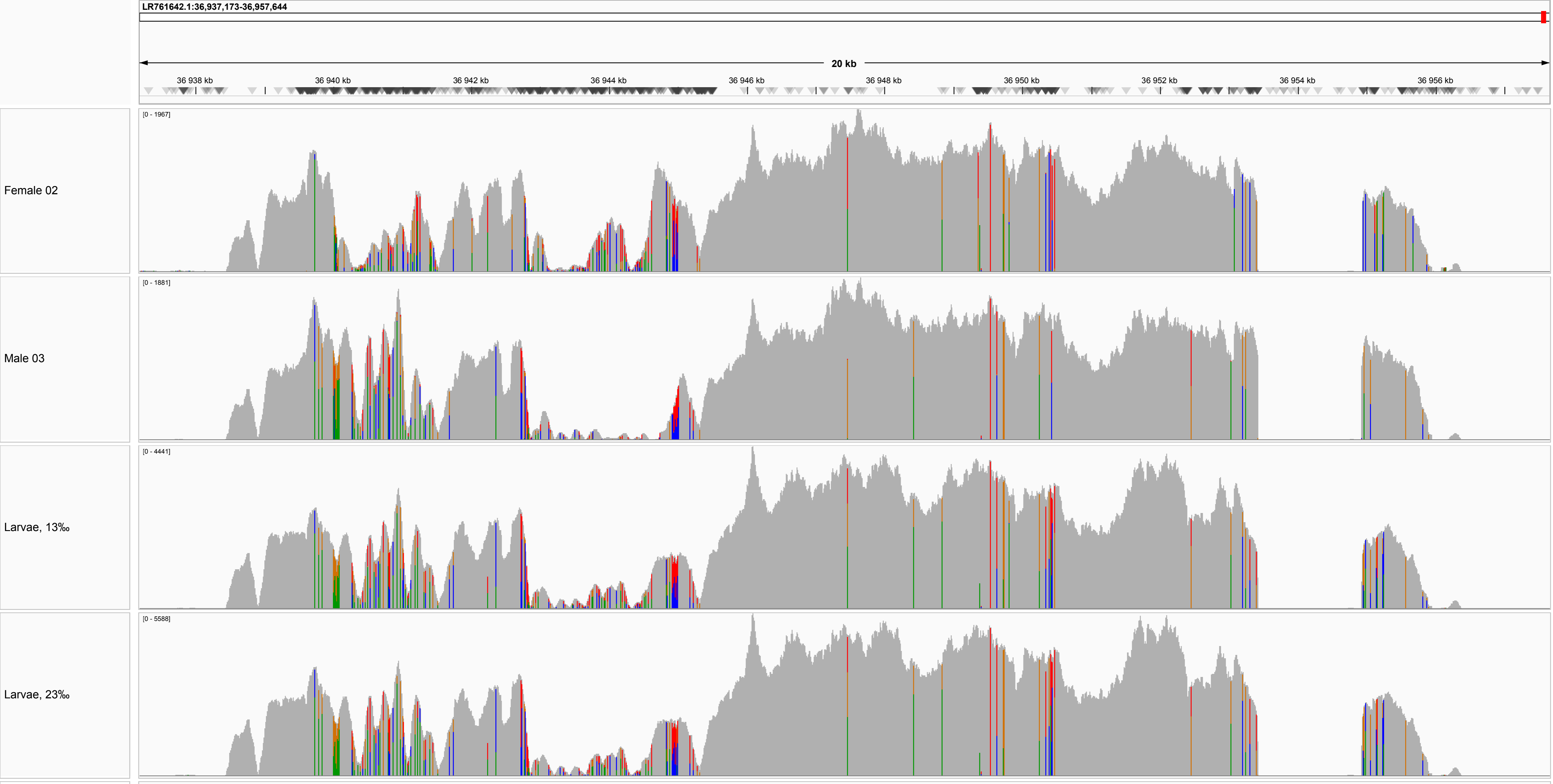

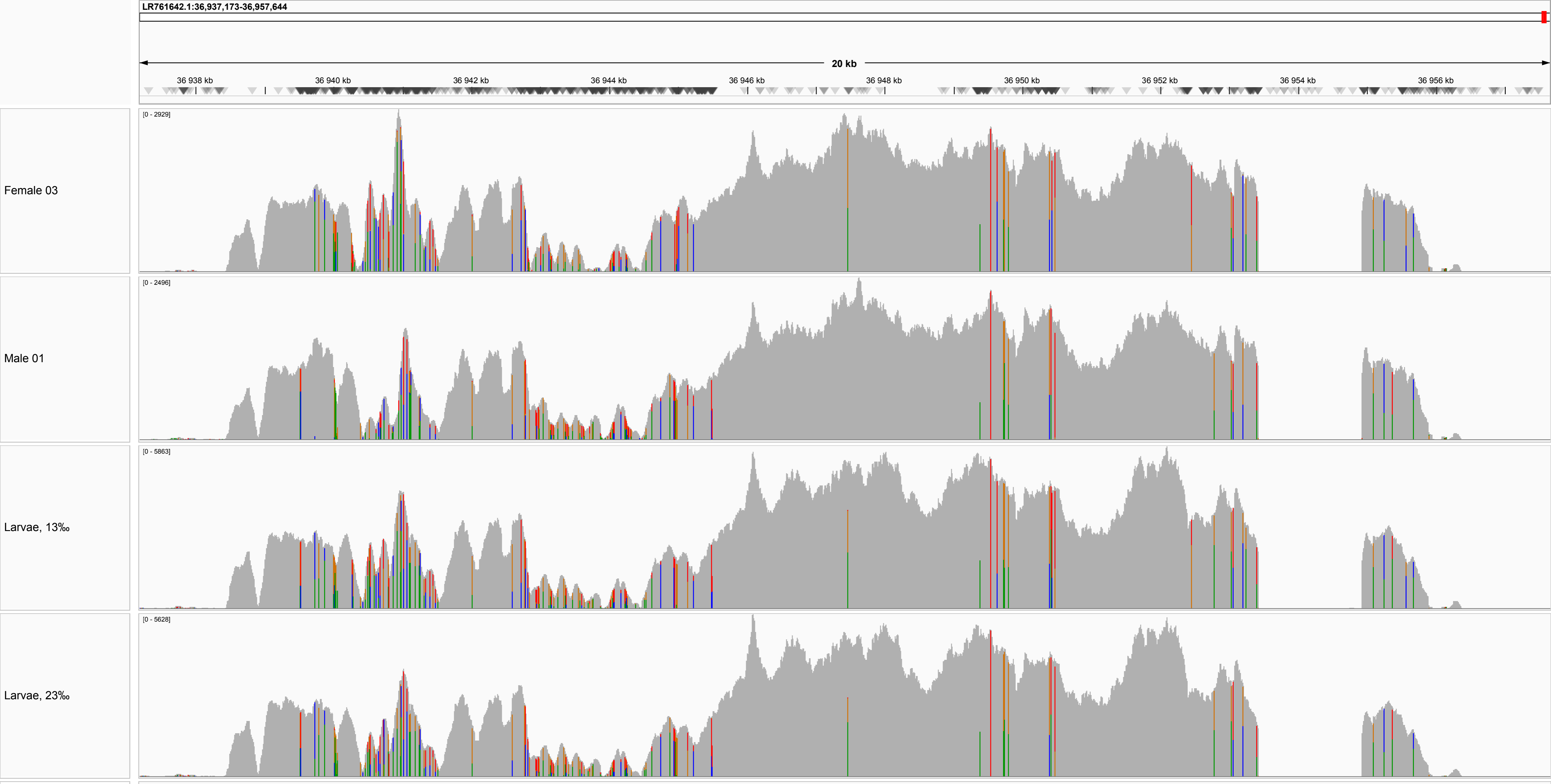

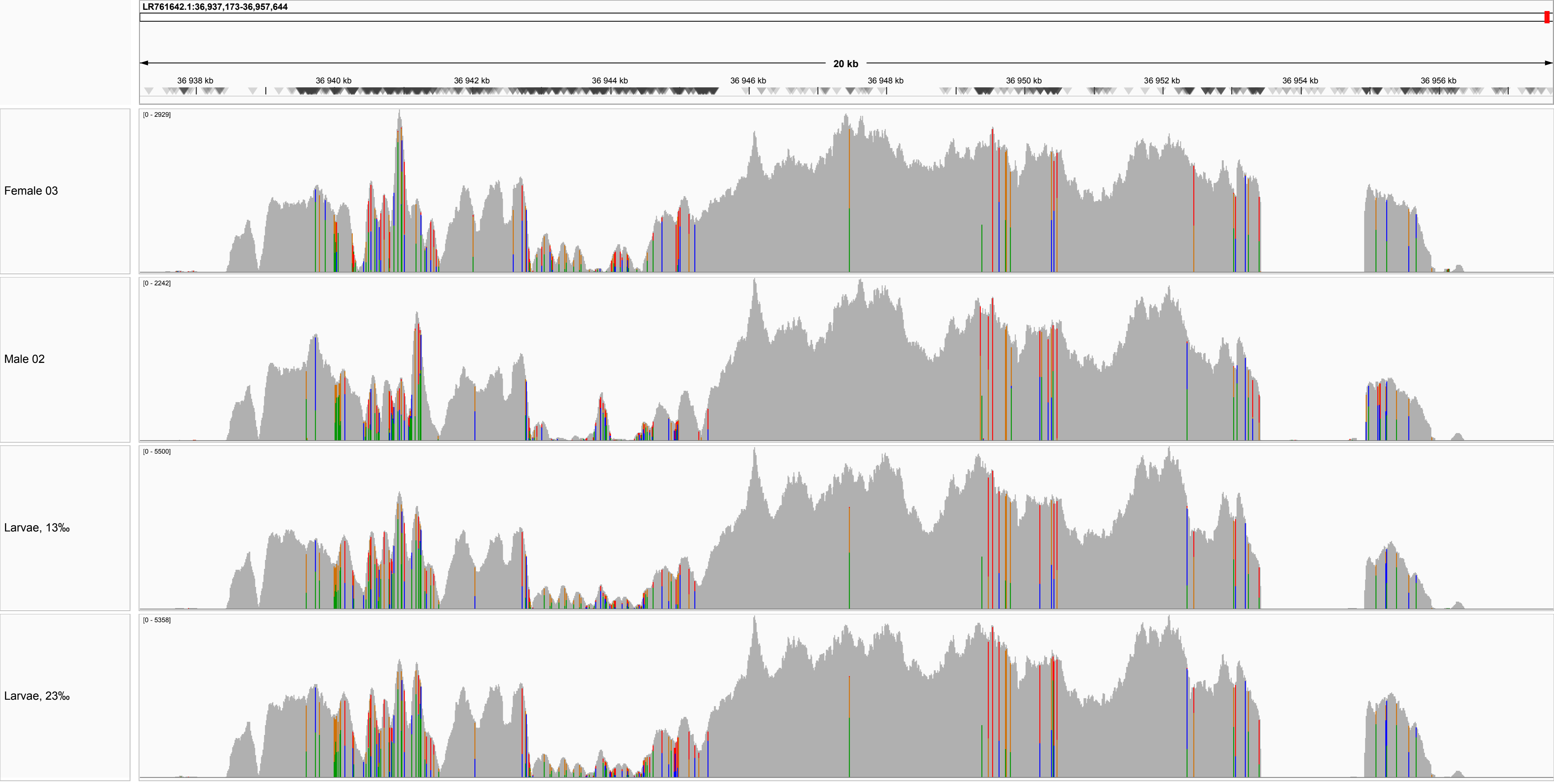

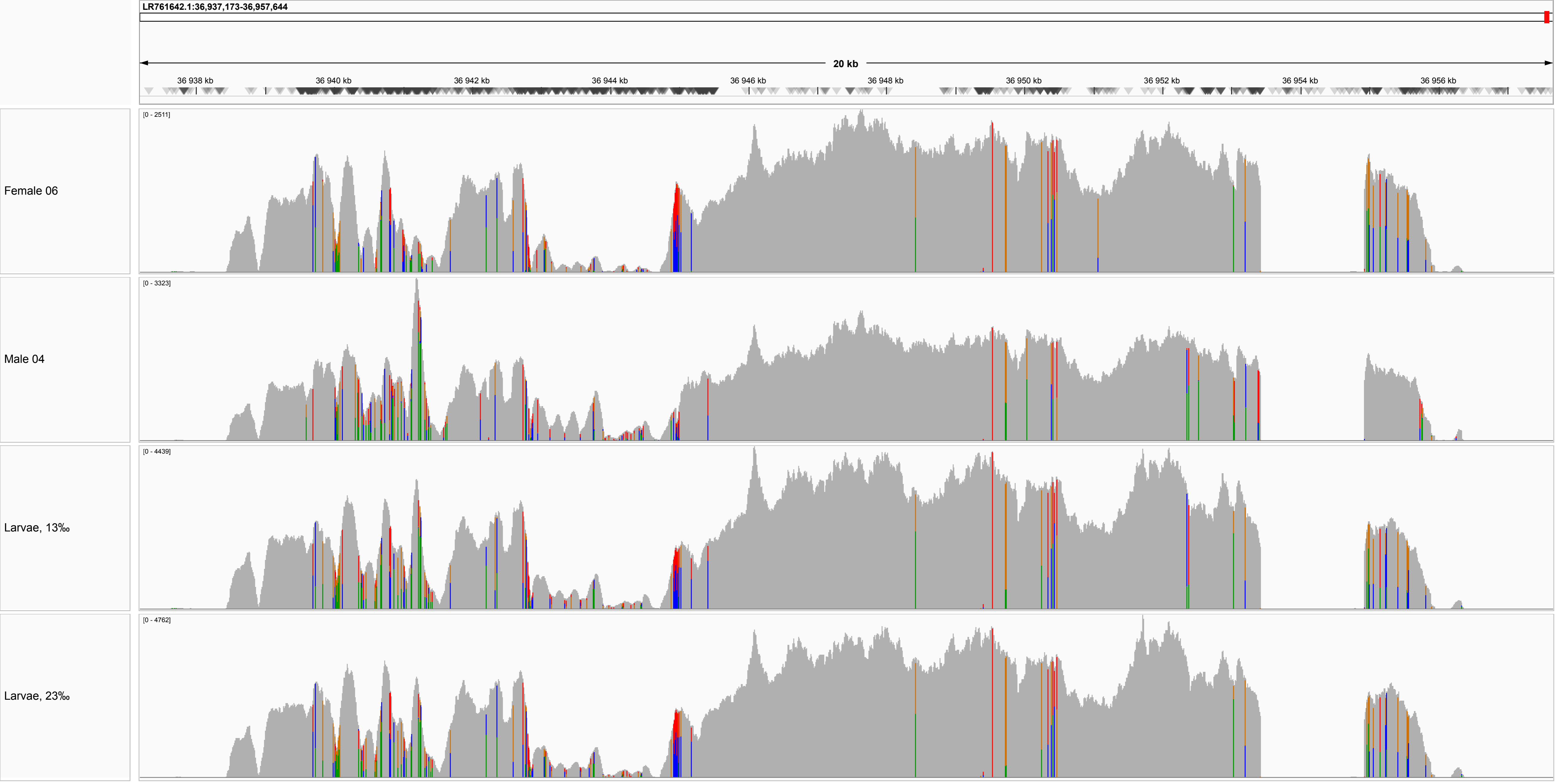

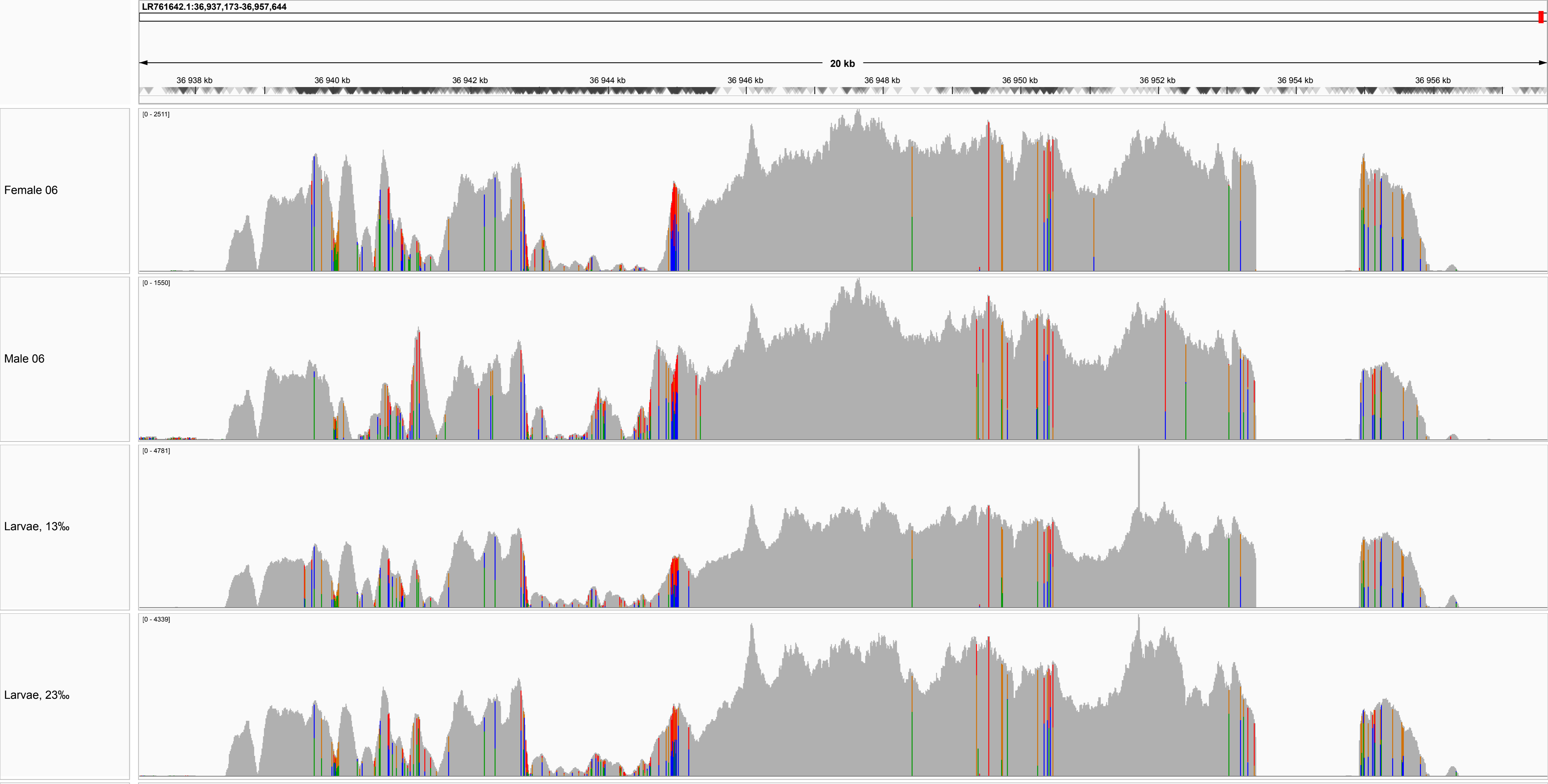

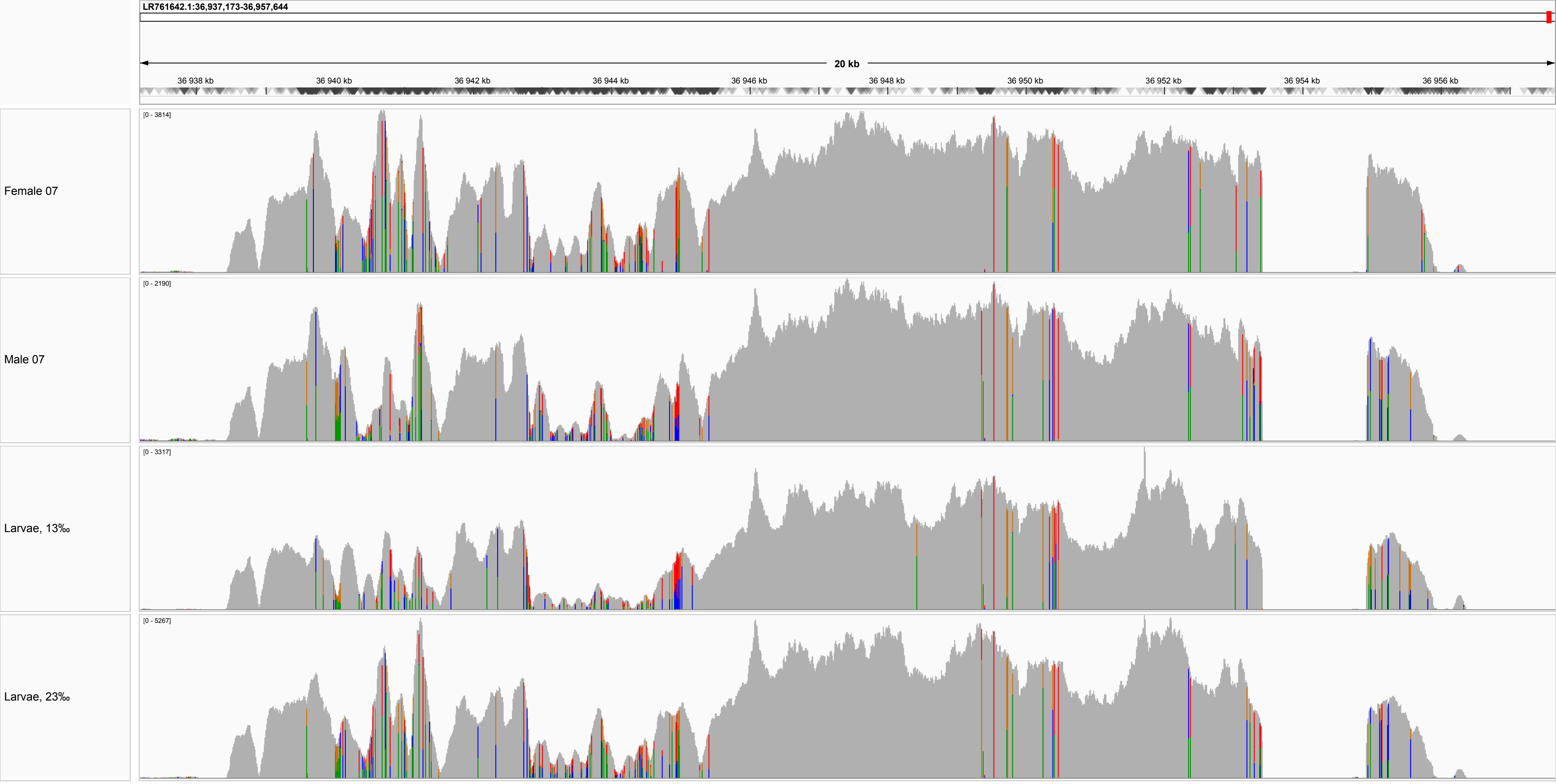

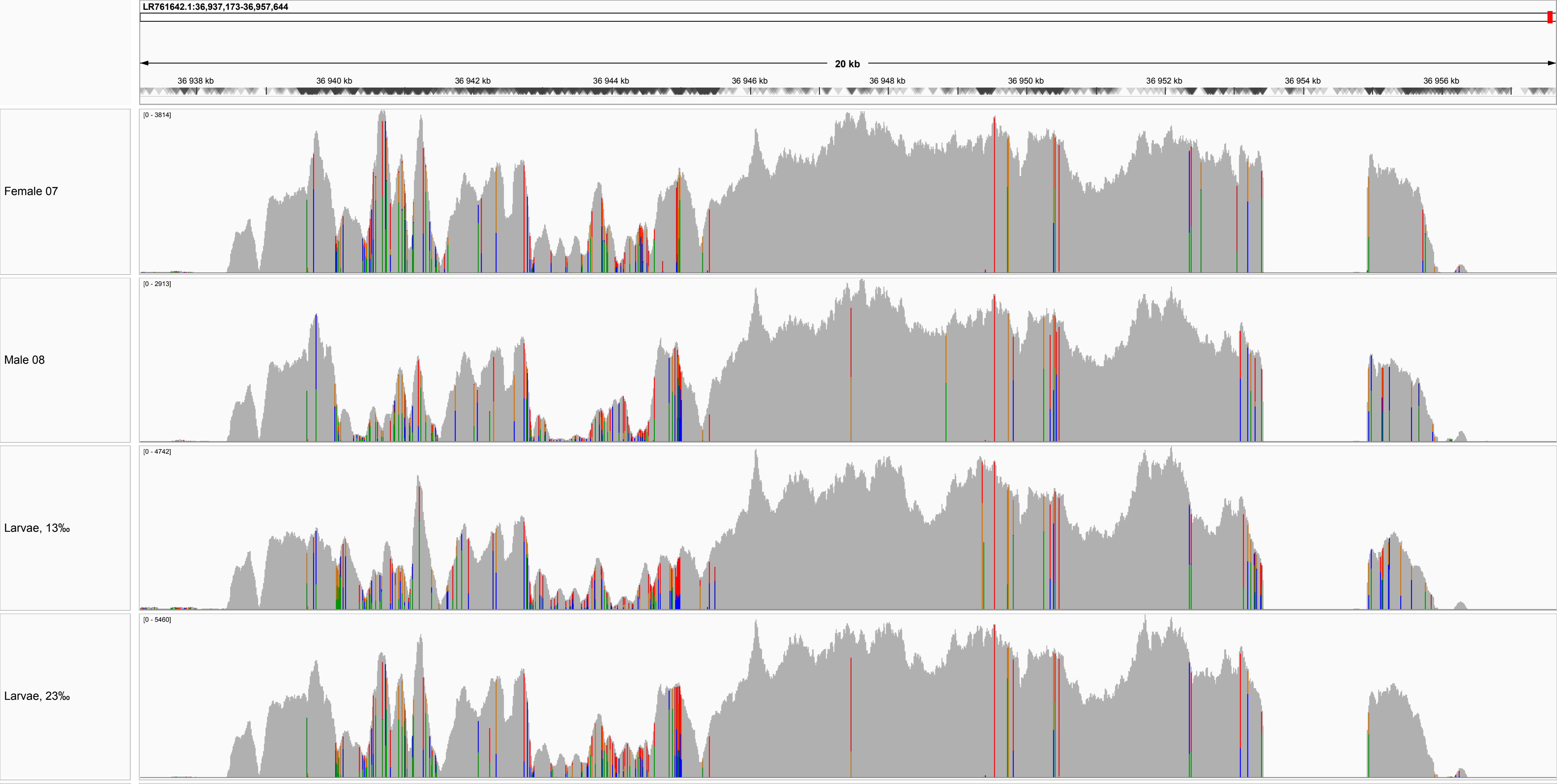

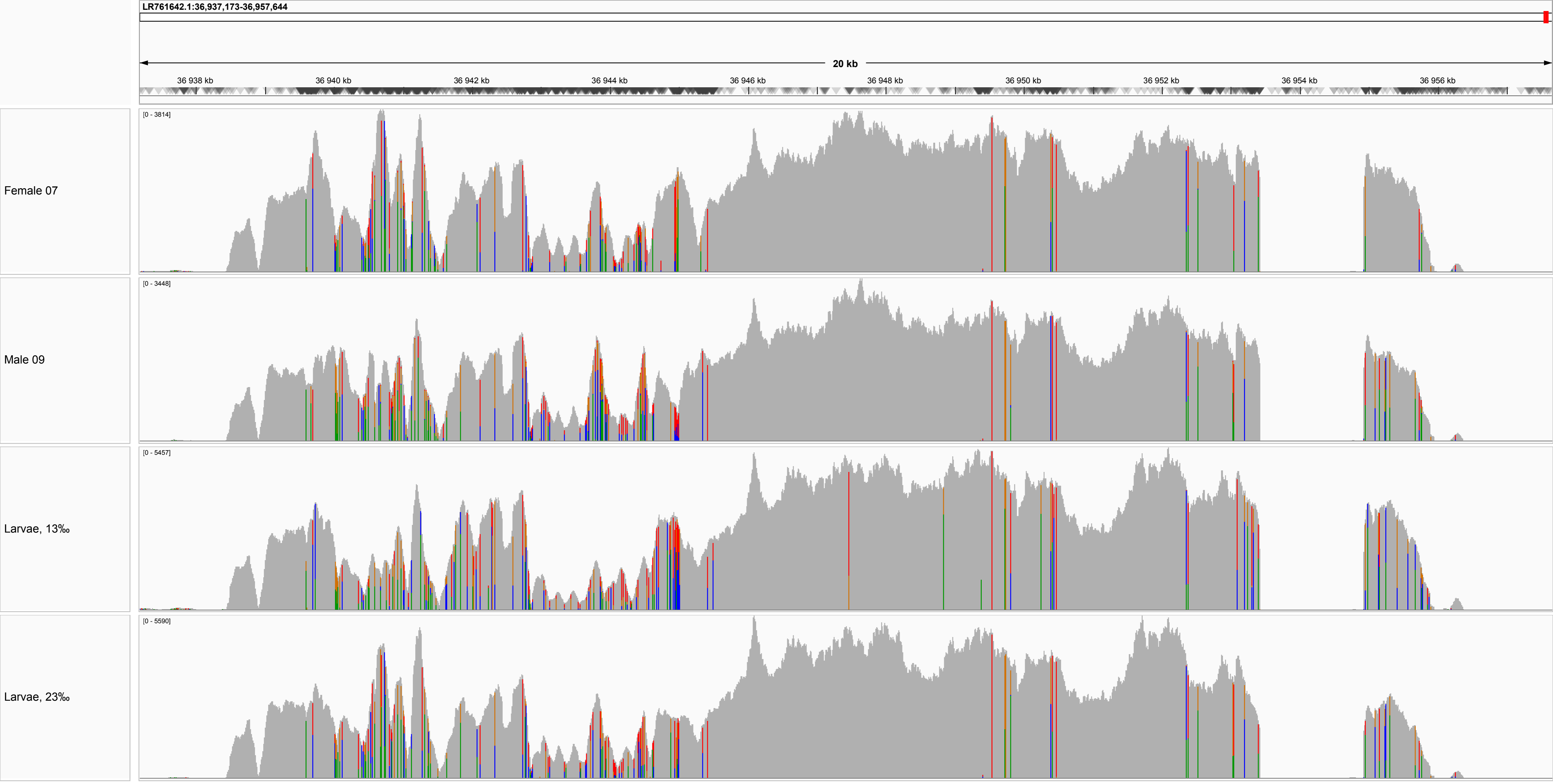

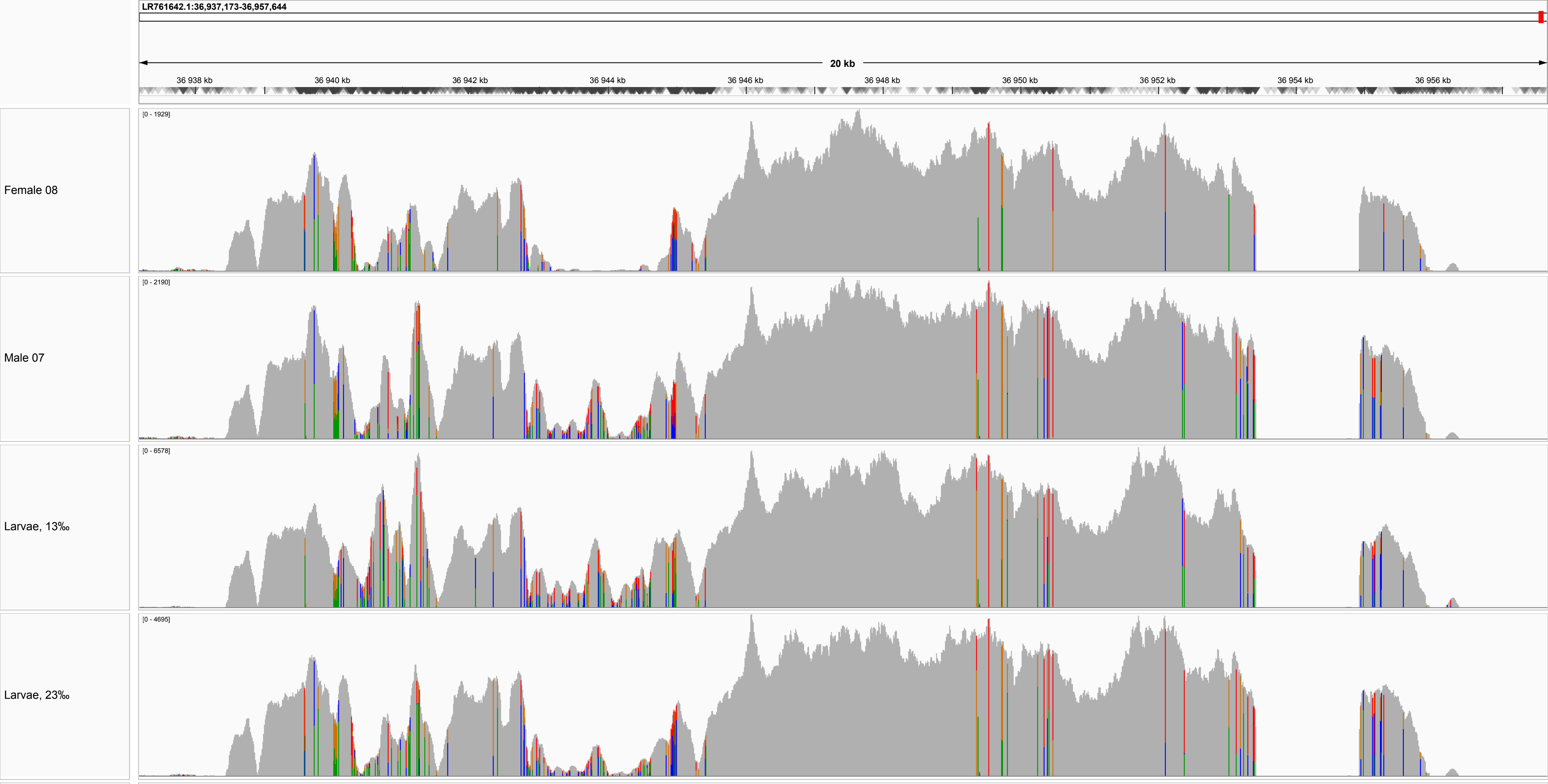

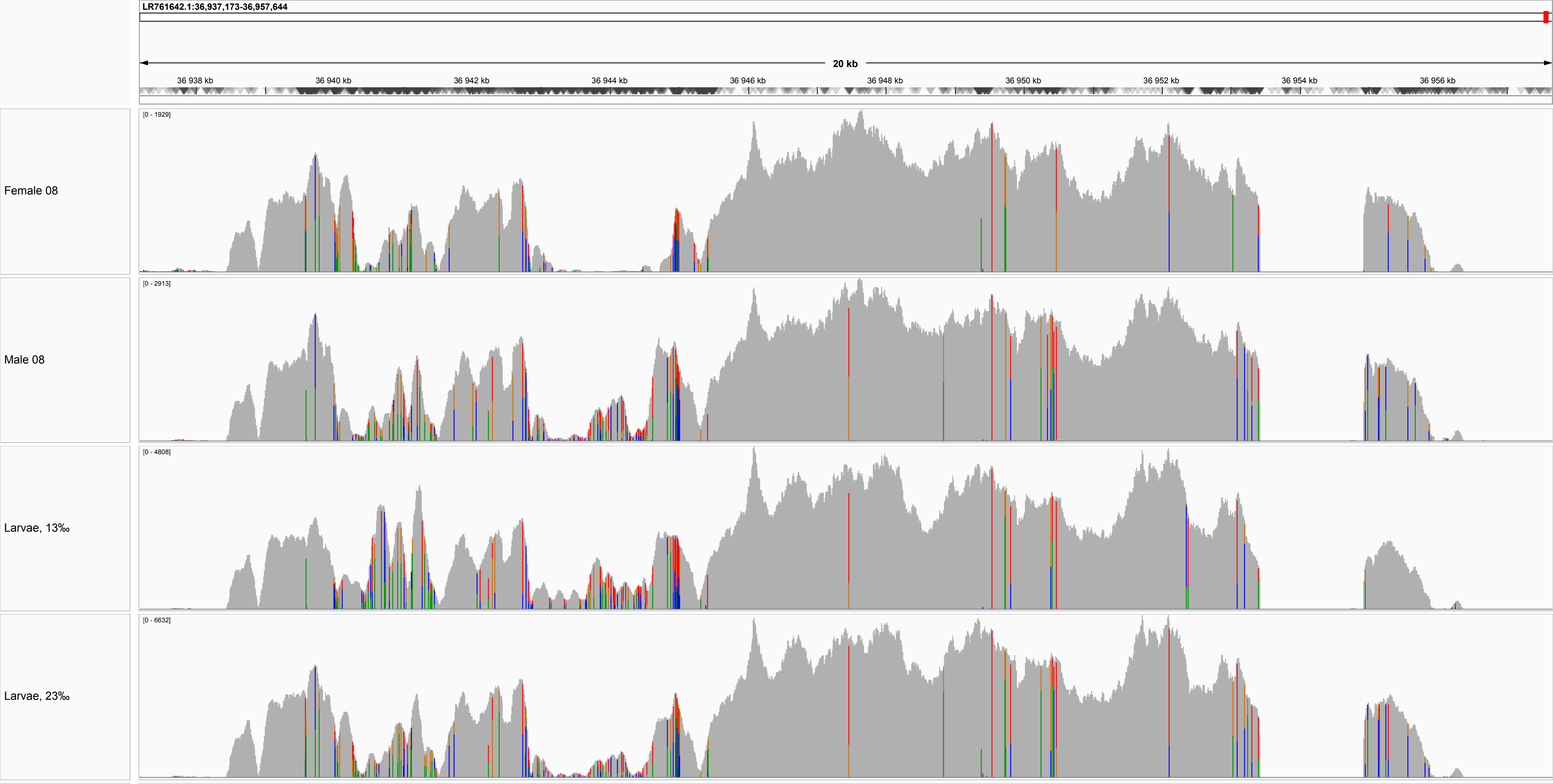

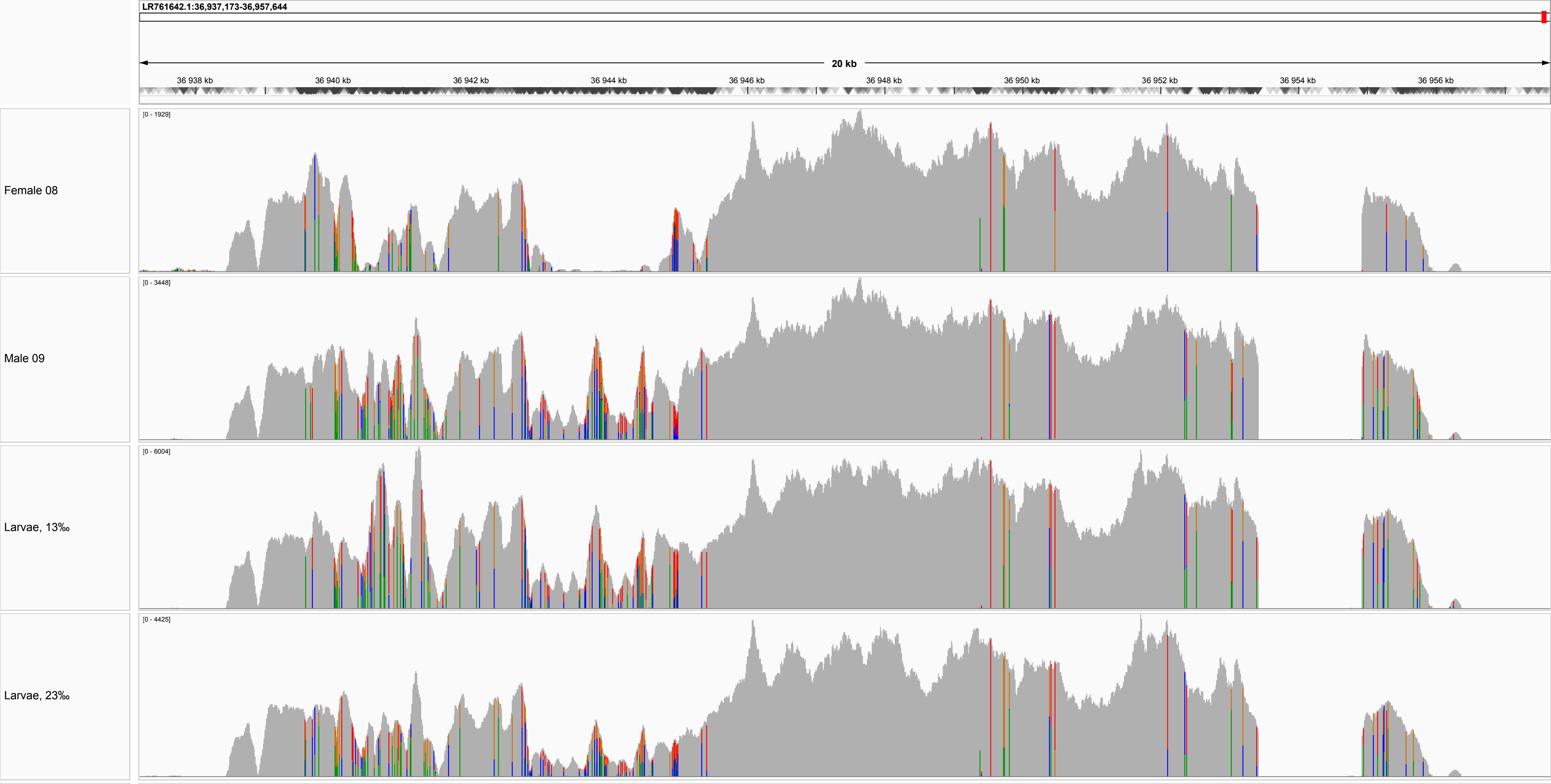

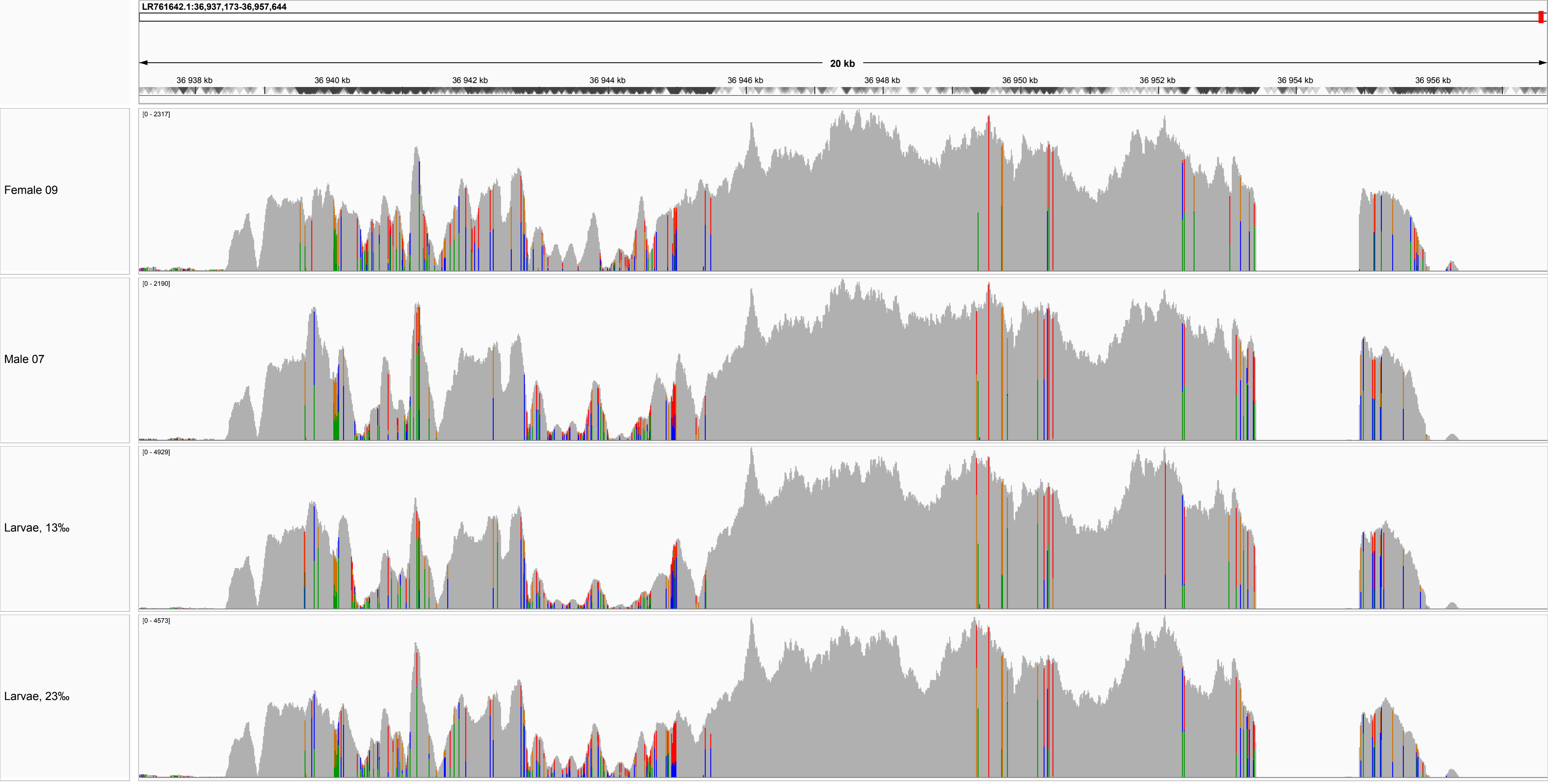

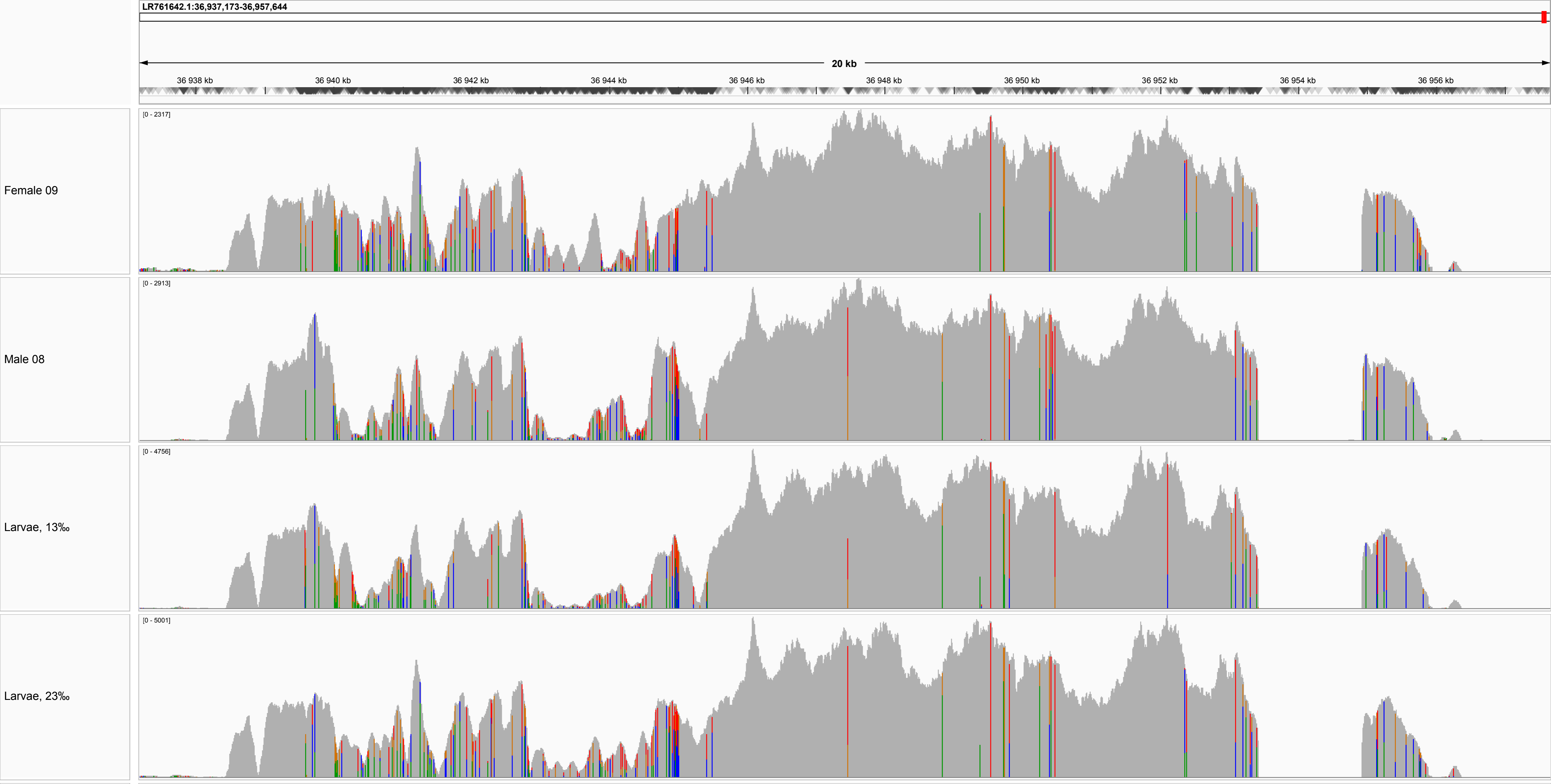

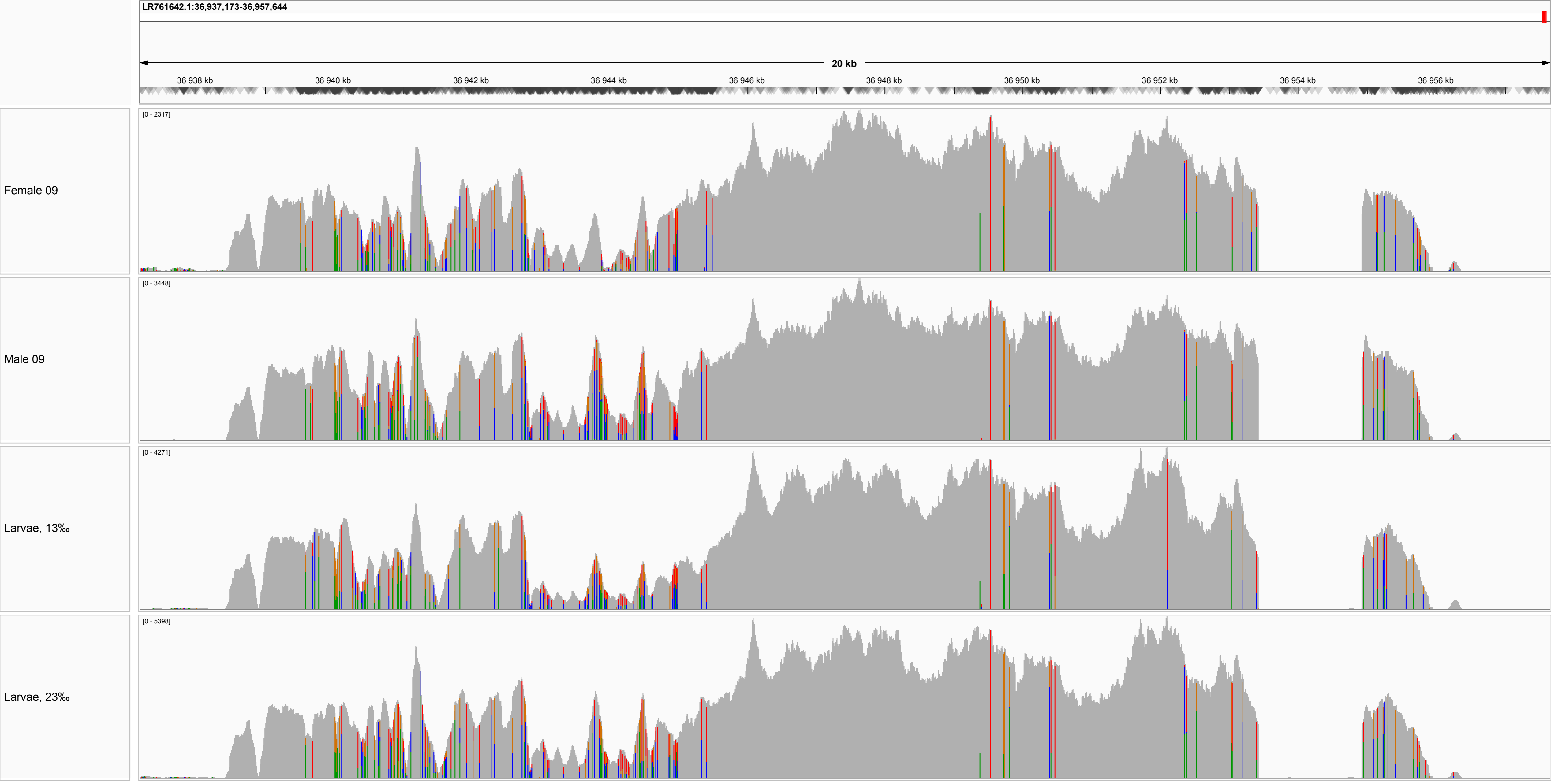

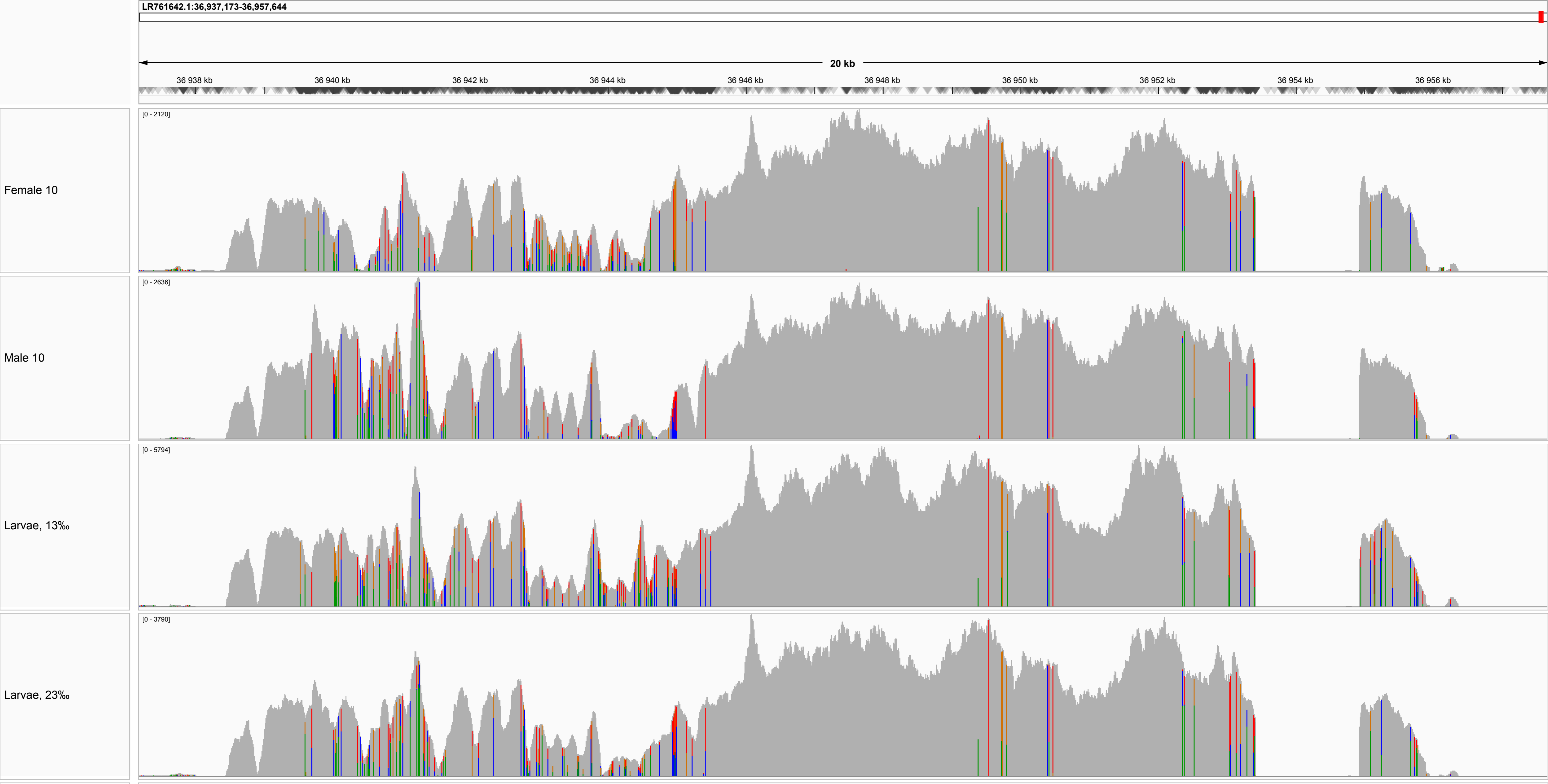

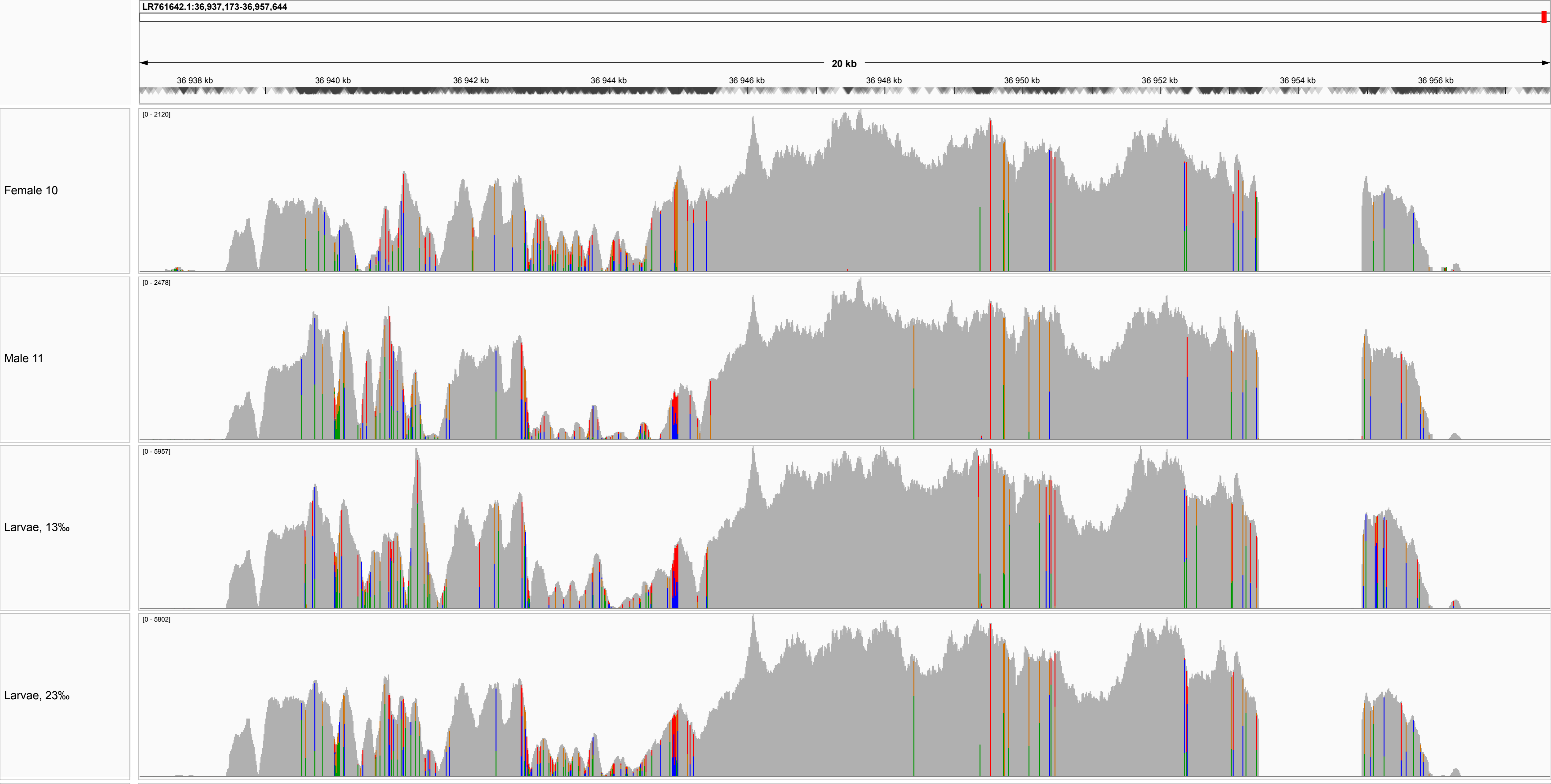

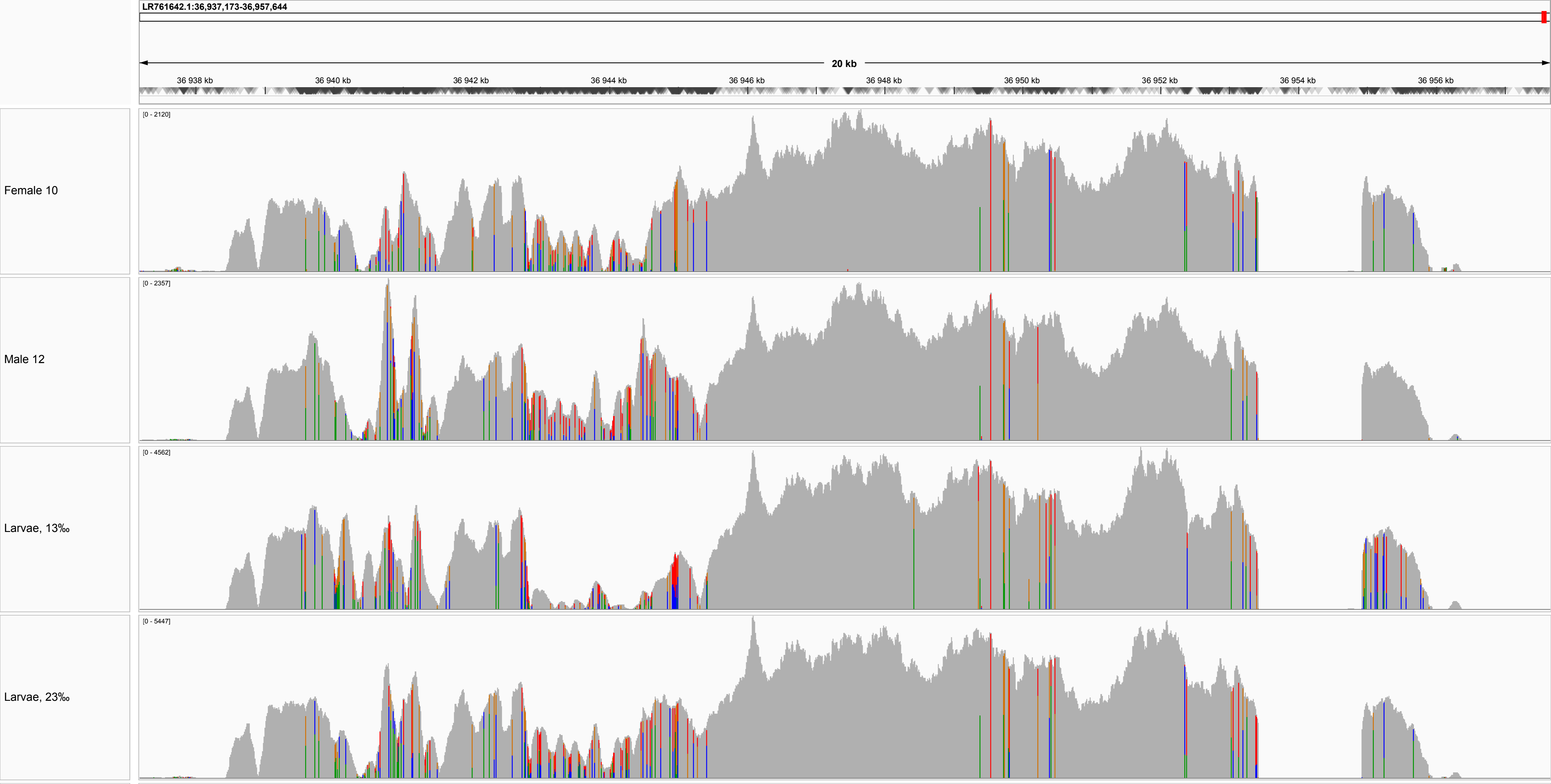

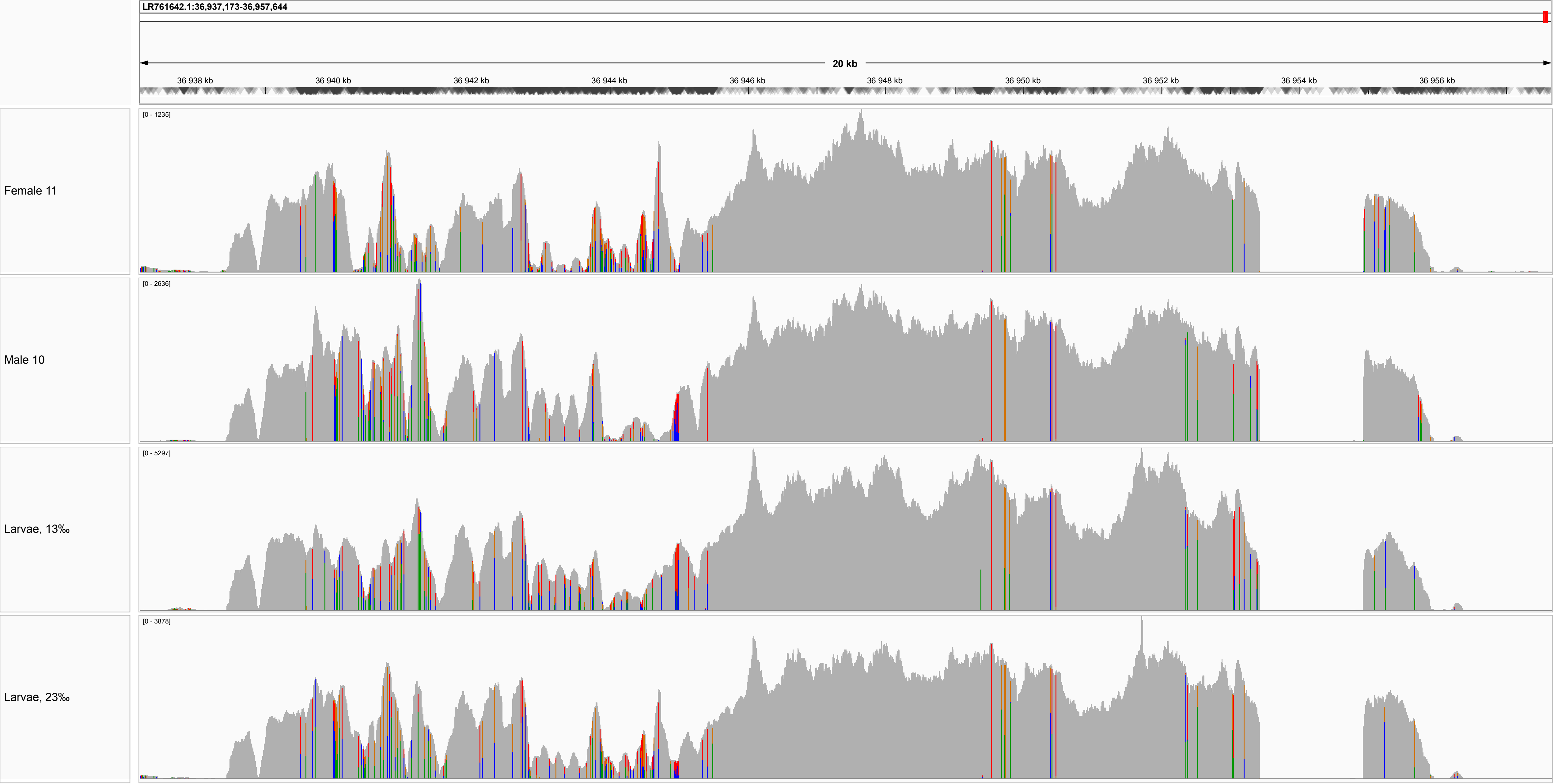

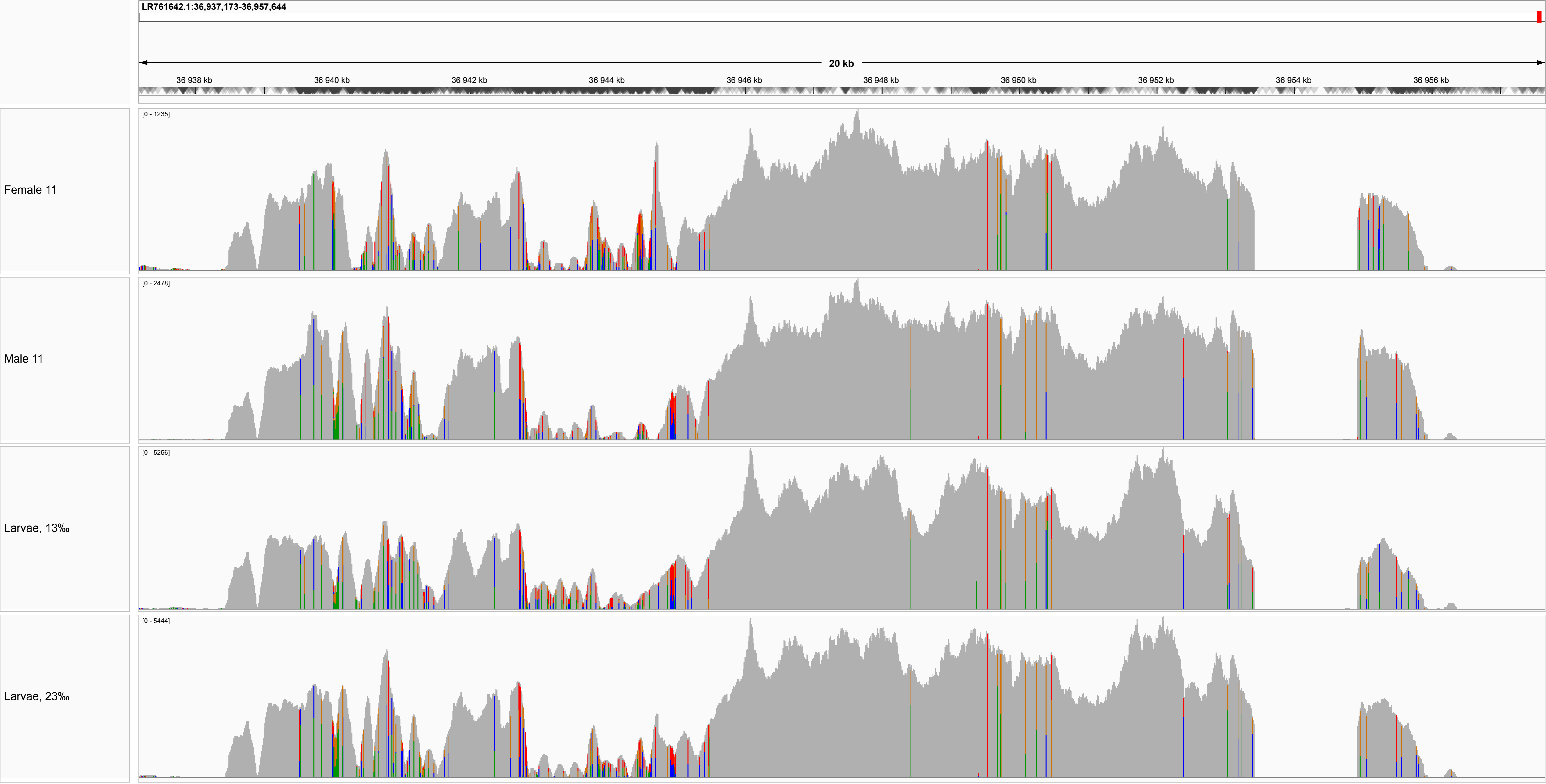

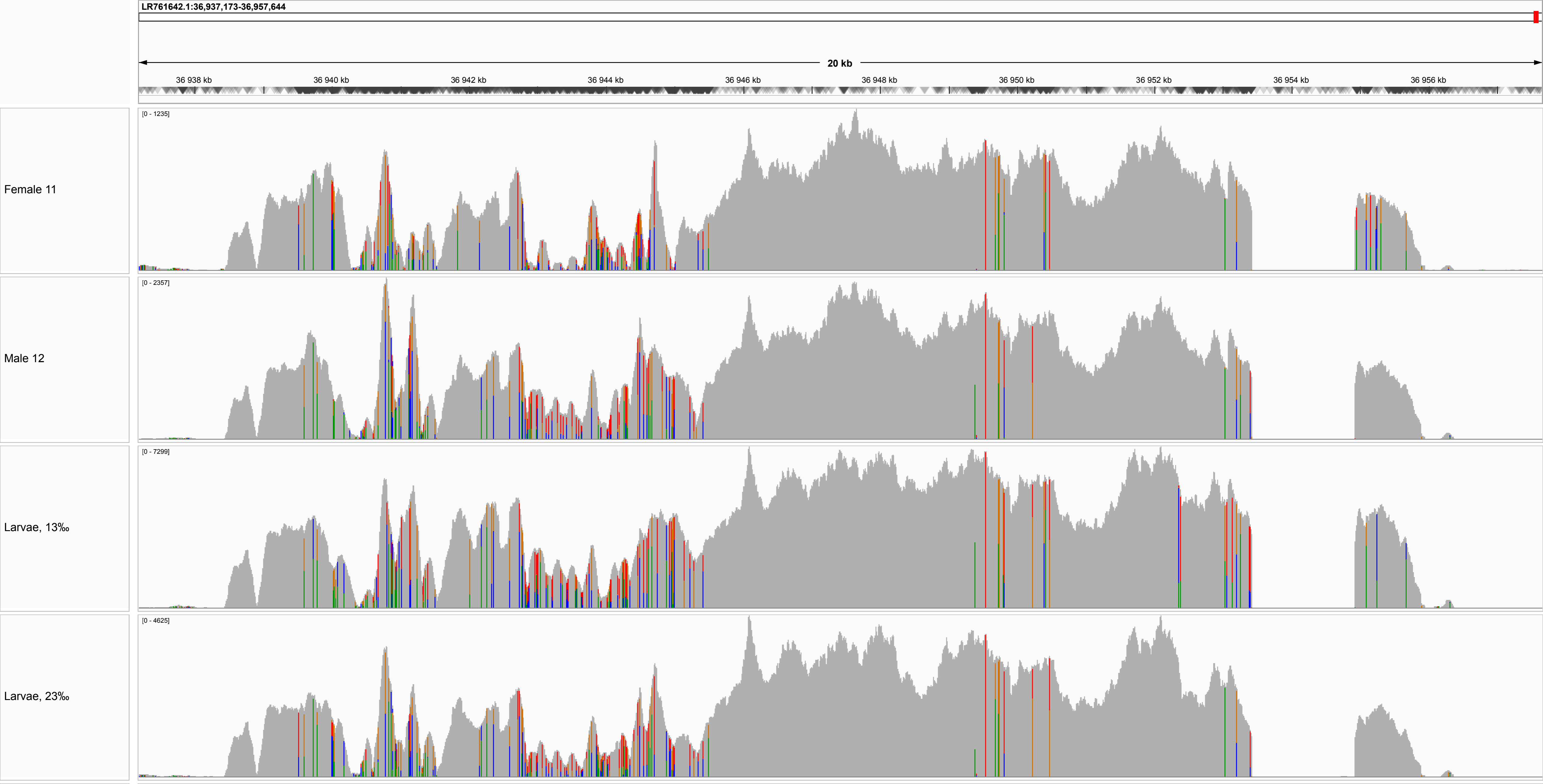

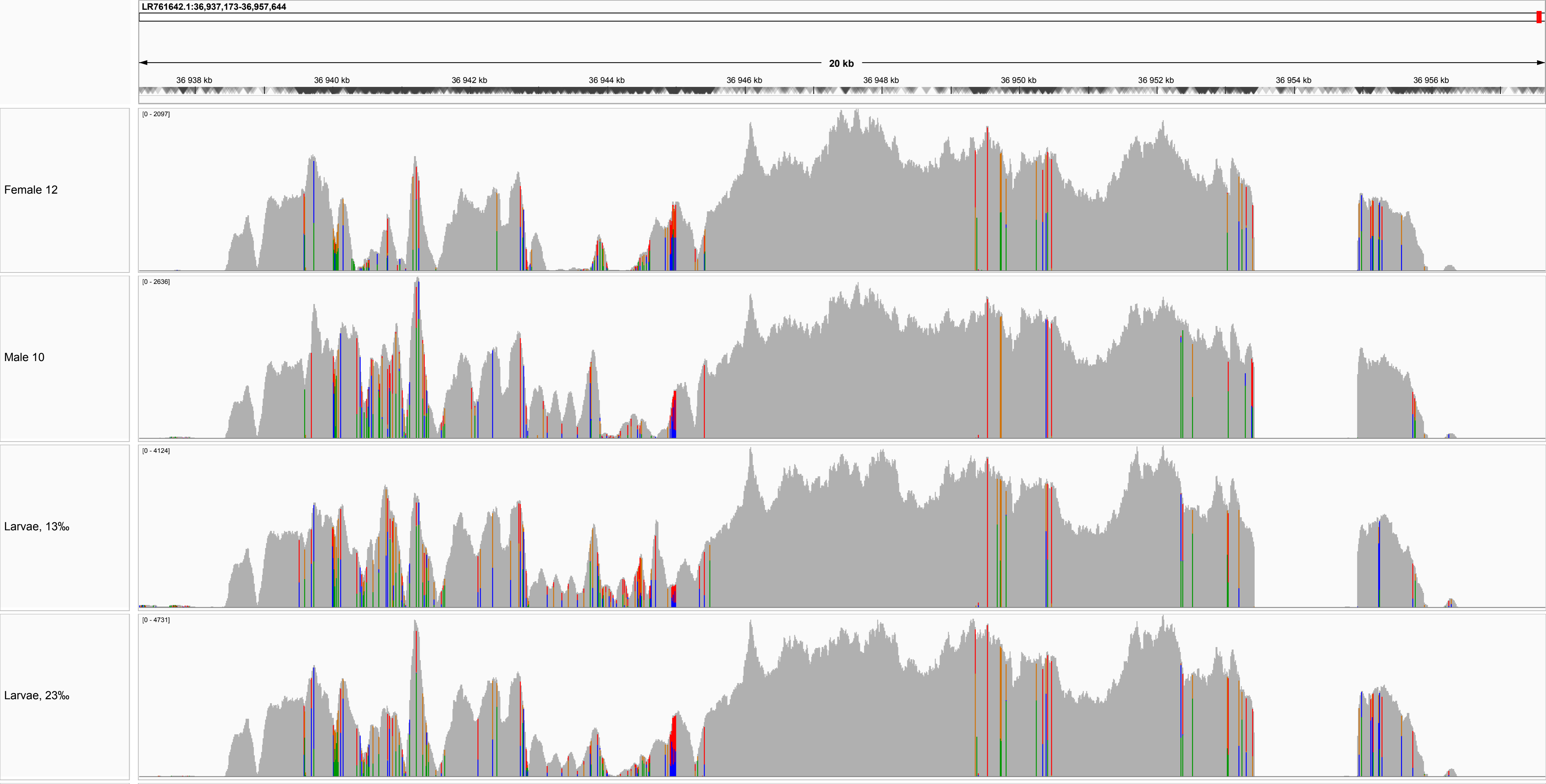



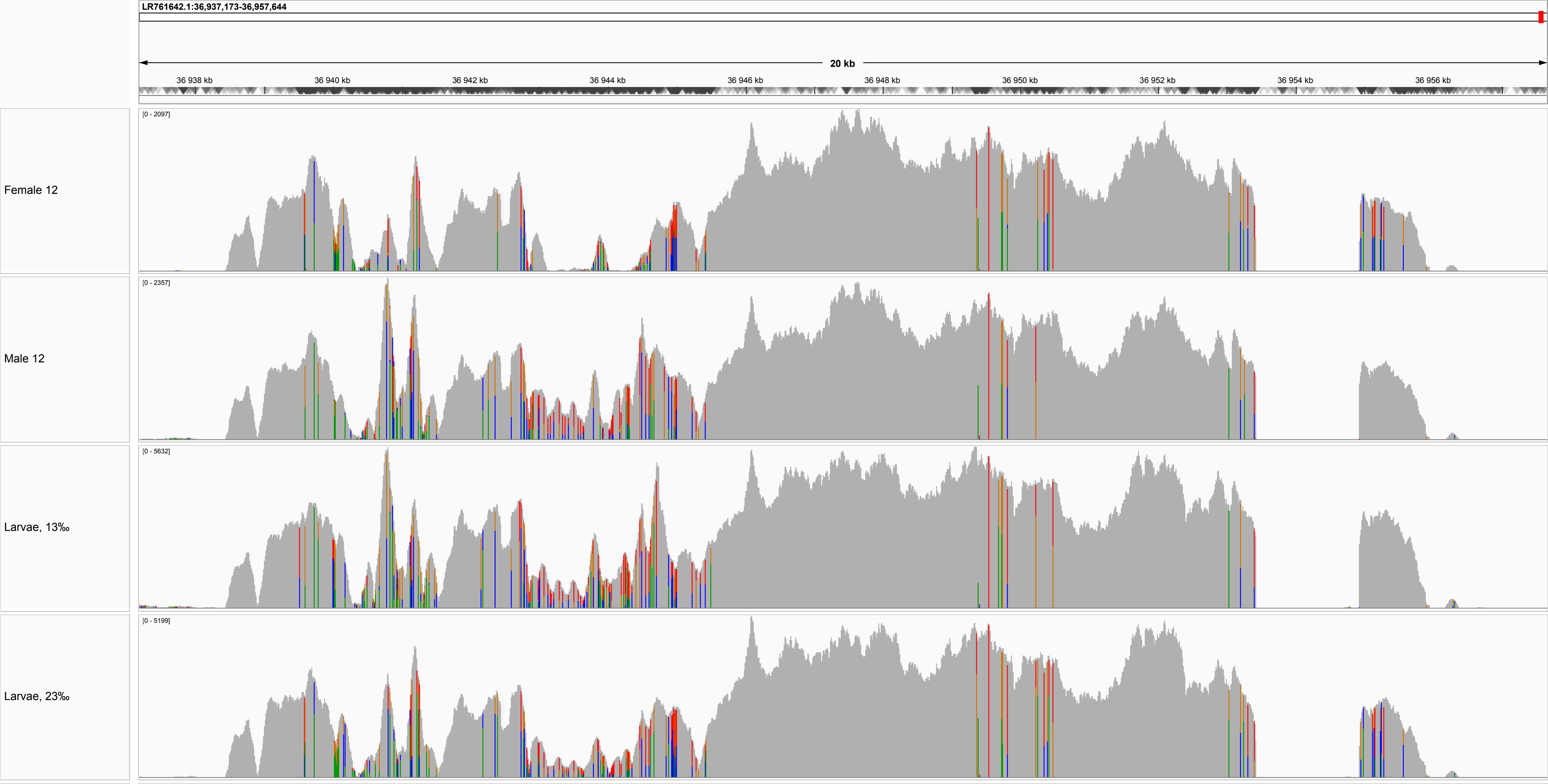

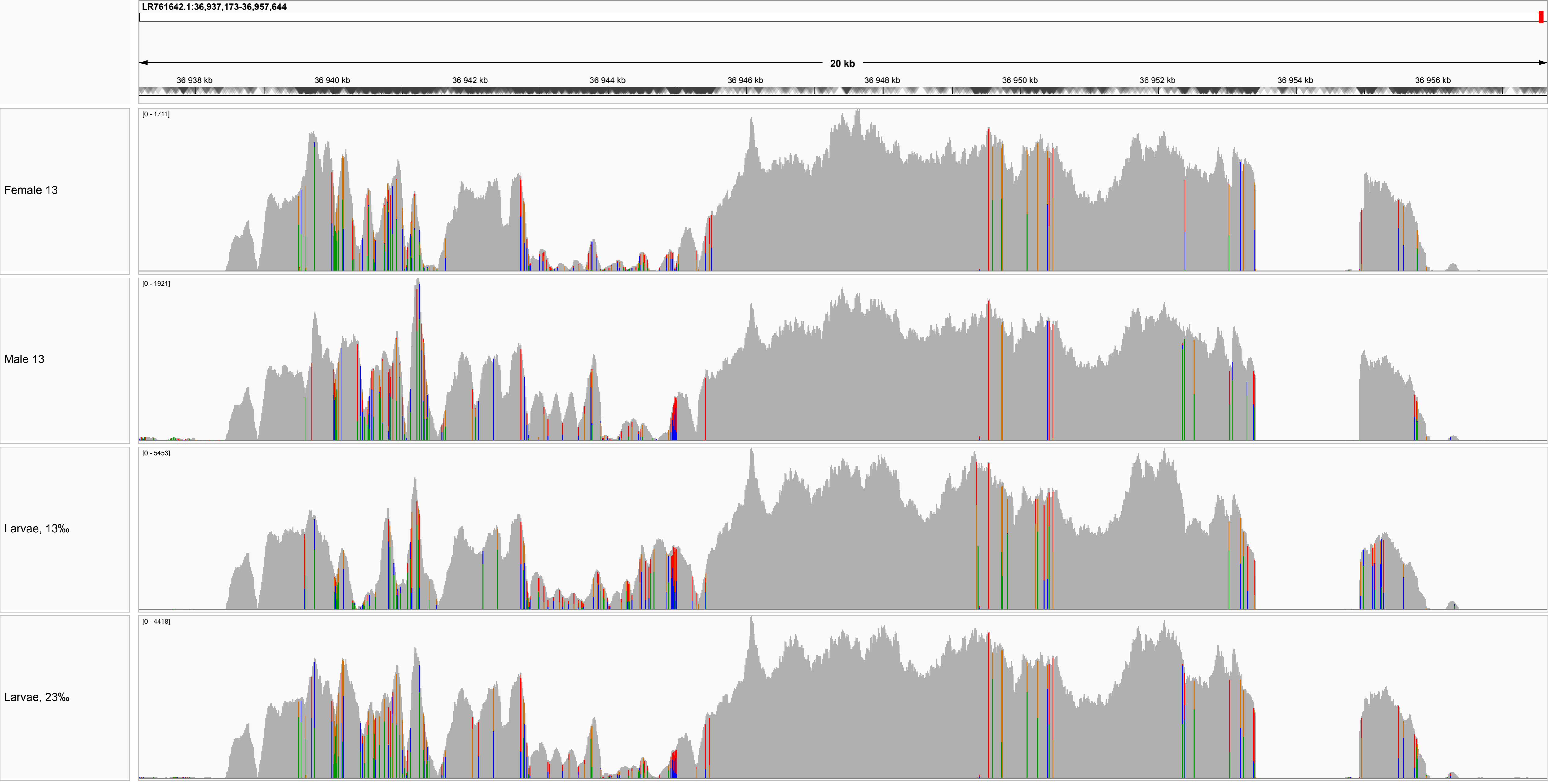

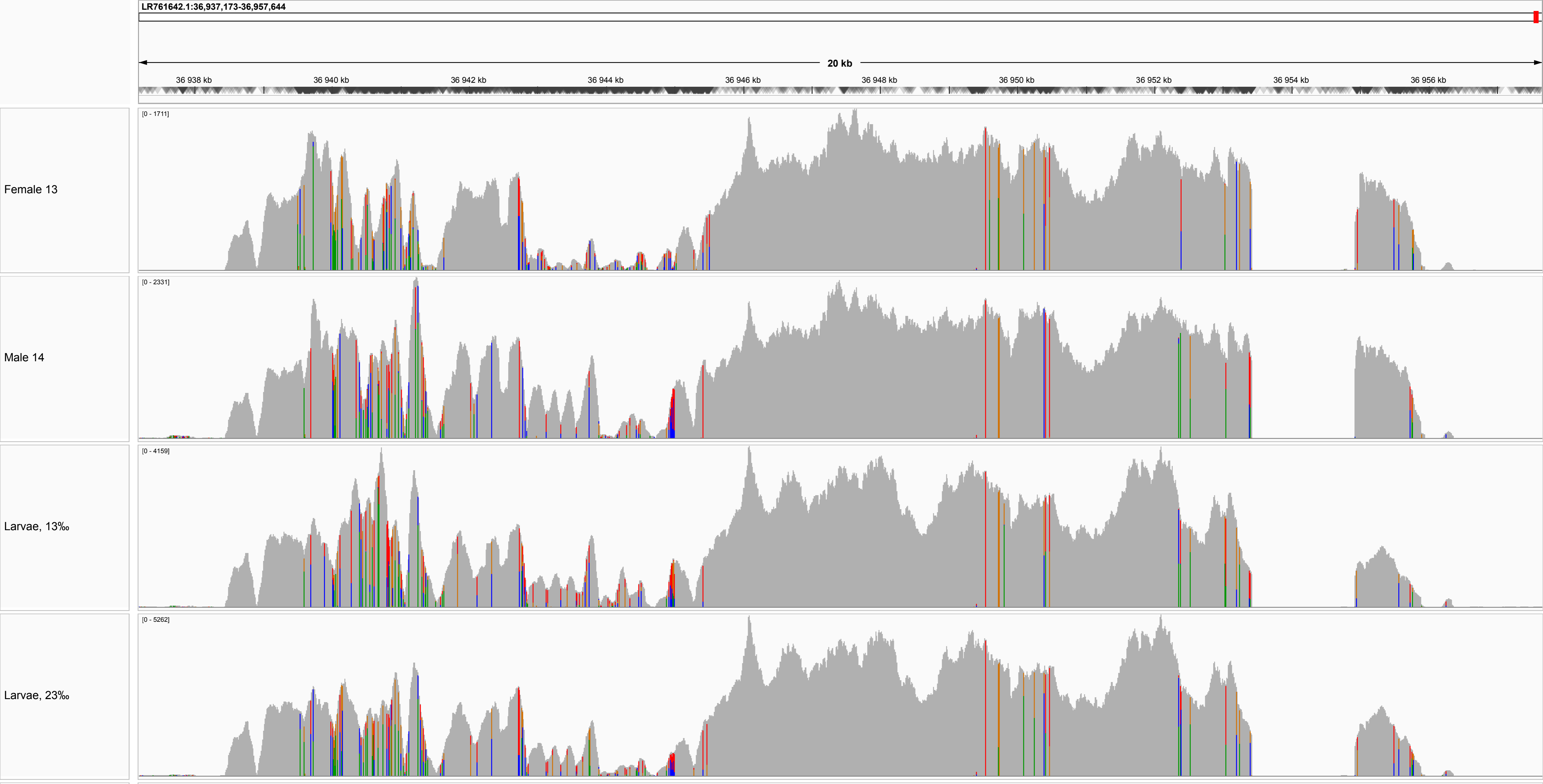

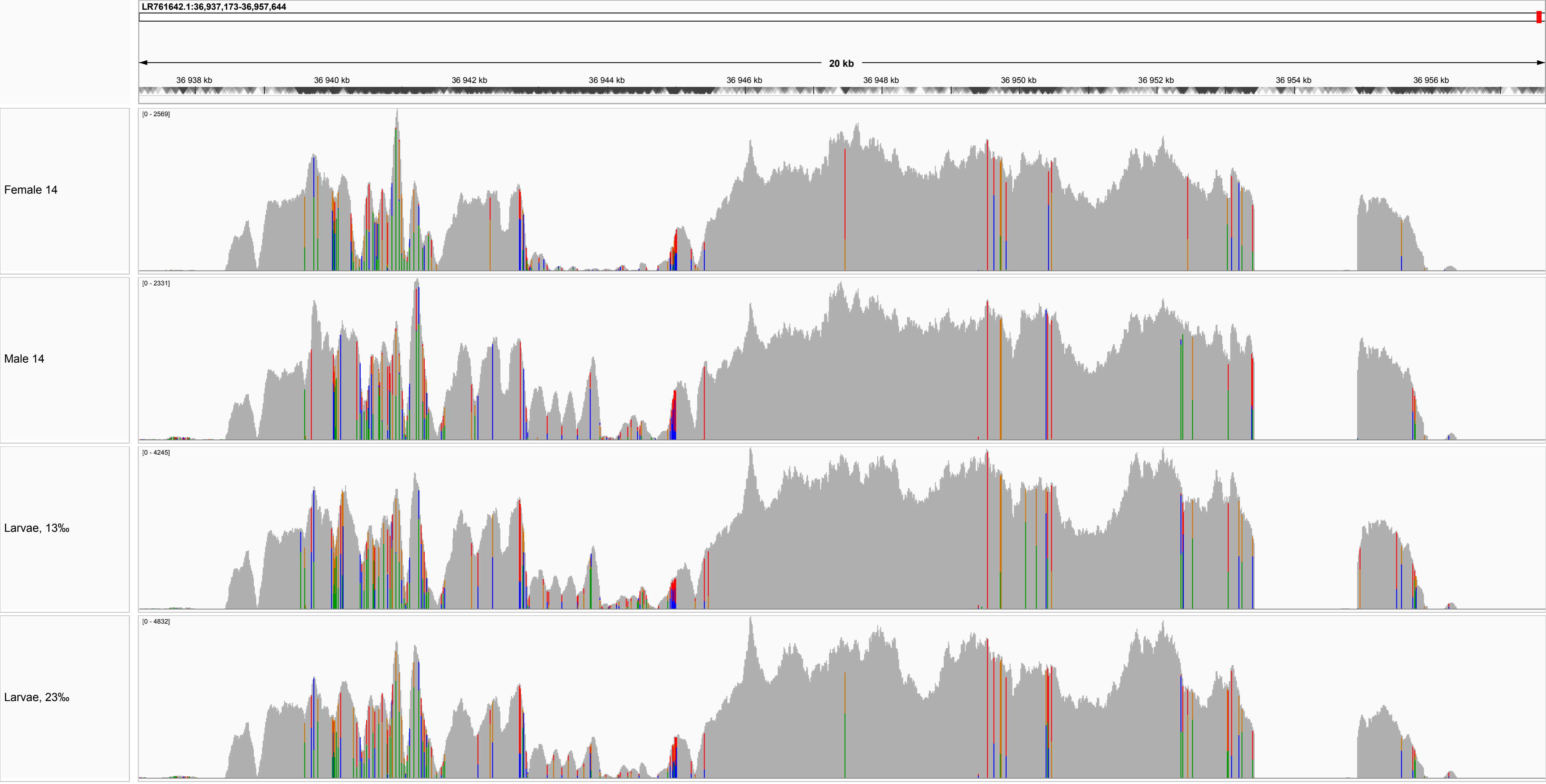

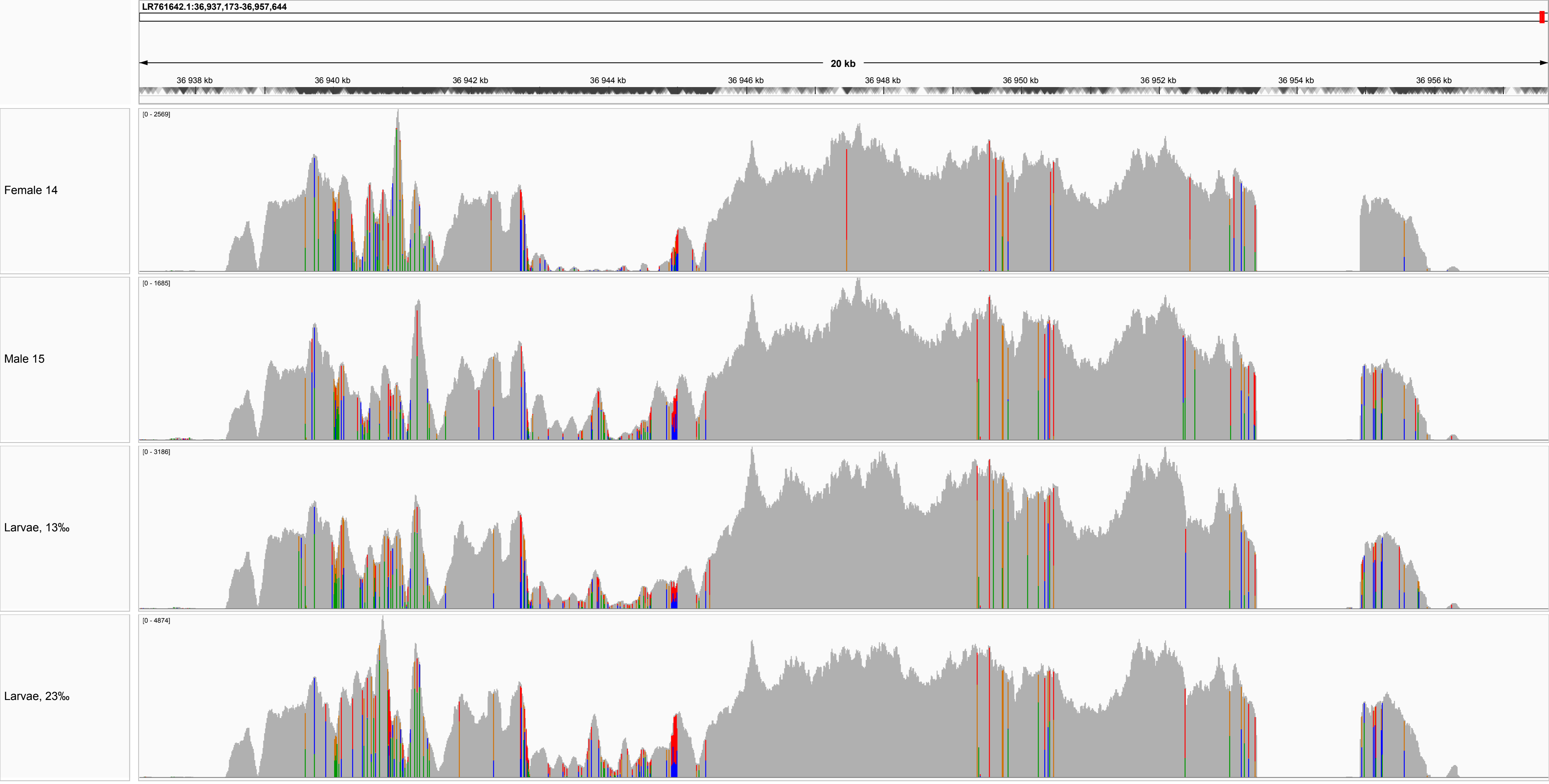

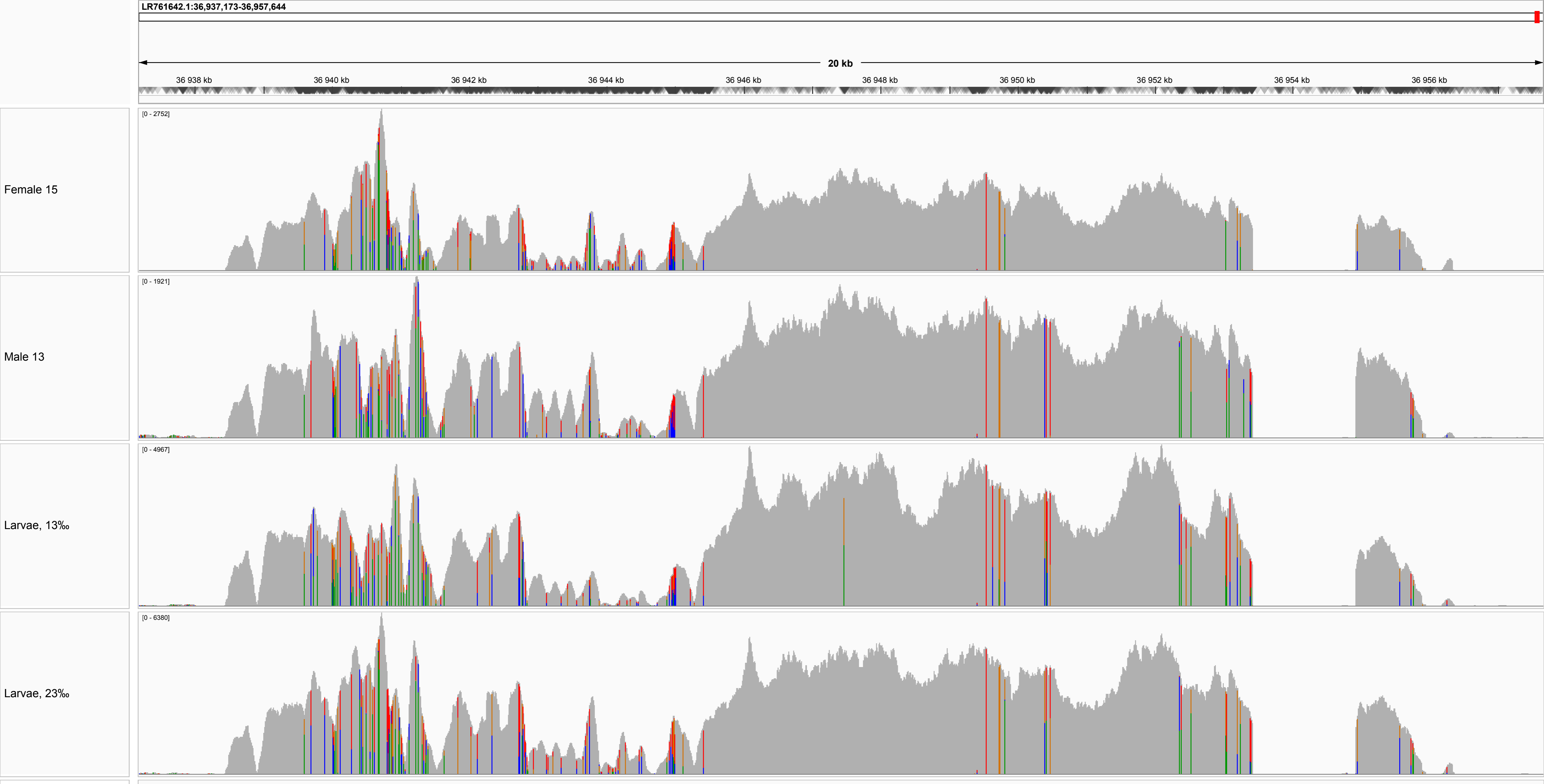

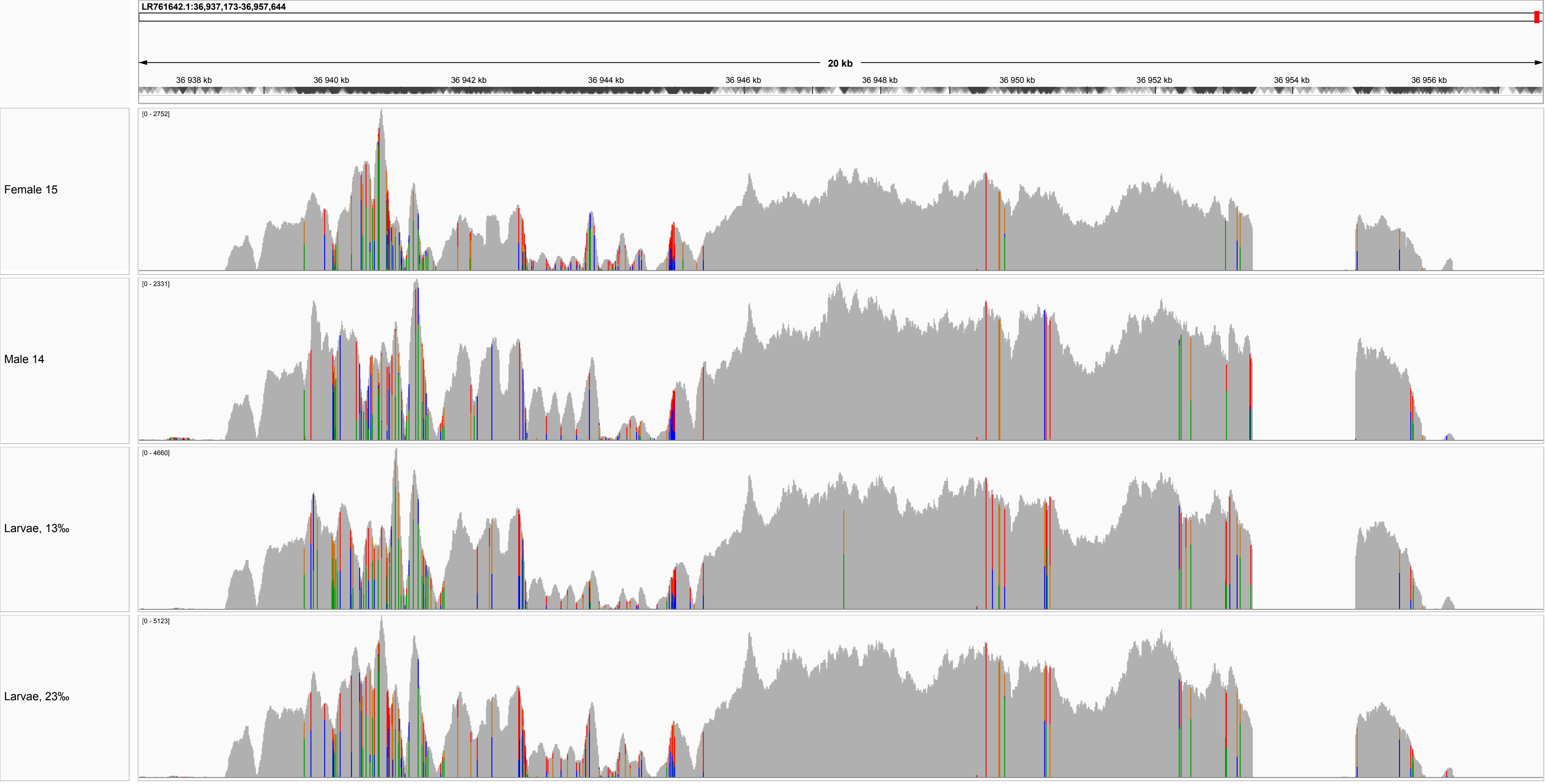

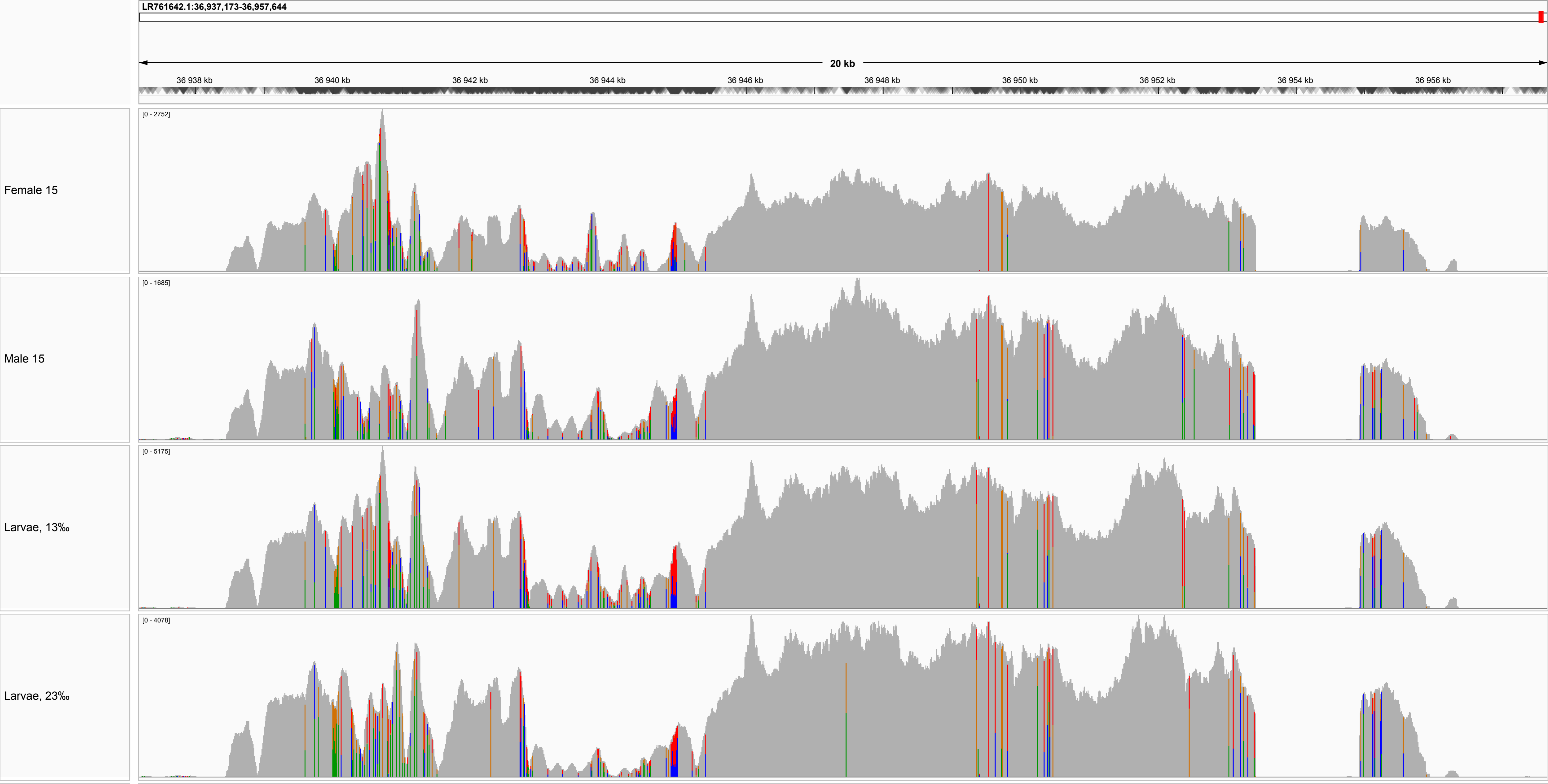

Supplement: Supplementary file 9 — Data S9. Alignment summary plots of the two annotated high ∆G peak regions in LG6 and LG9 showing sequencing depth along the reference sequence as well as sequence divergence from the reference sequence (highlighted in colour:). The sequencing depth scale for each plot (min‐max) is given in brackets on the top left of each plot. Plots are organised in family tetrads for each family from crosses of oysters from the present invasion front, from top to bottom: Female (Mother), Male (Father), Larval full‐sib family in salinity 13‰, Larval full‐sib family in 23‰. [file MEC-34-e17684-s004.pdf]

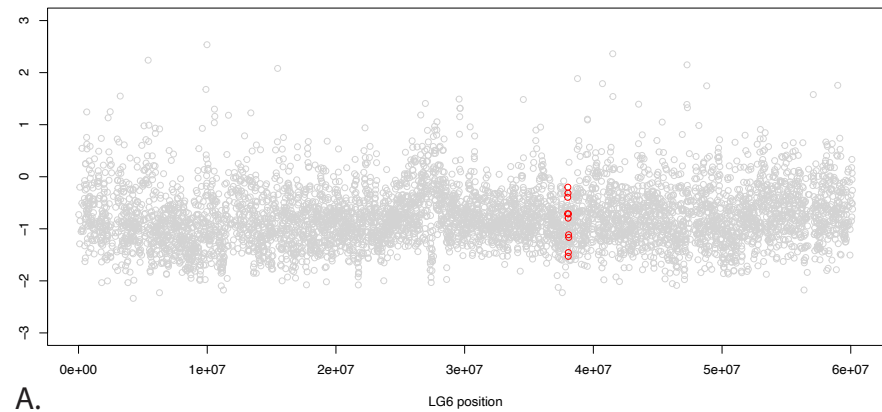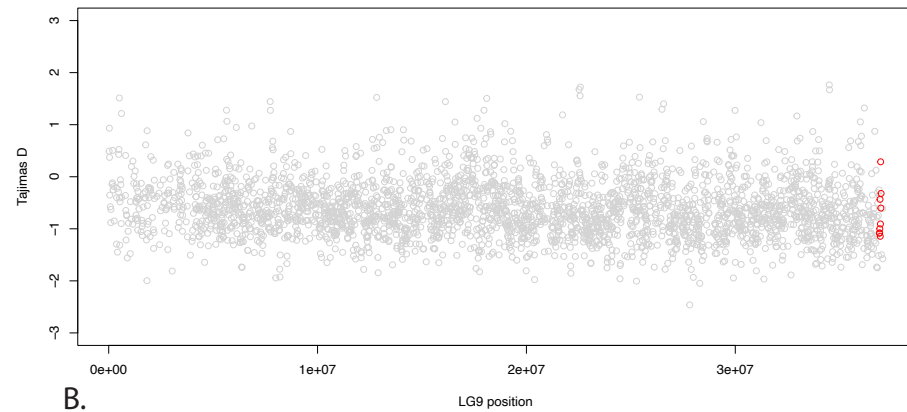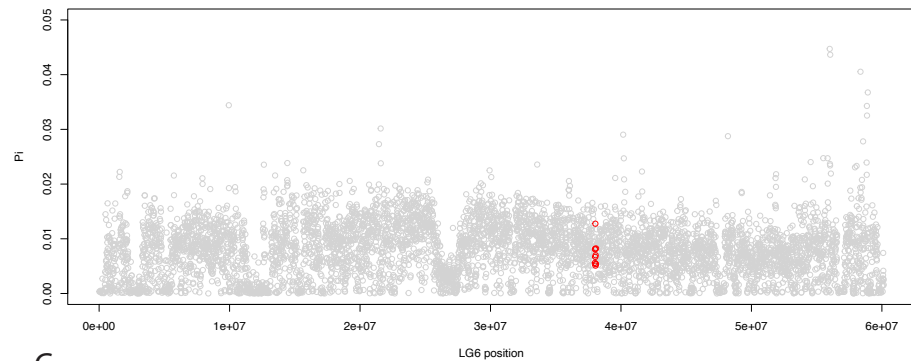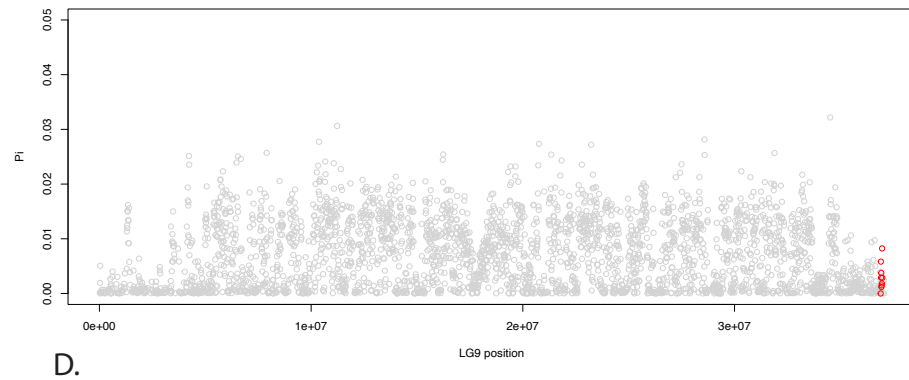

Supplementary Data S9. Nucleotide diversity and Tajima's D along LG6 and 9.

Supplement: Supplementary file 10 — Data S10. Nucleotide diversity and Tajima’s D along LG6 and 9. [file MEC-34-e17684-s001.pdf]
