# Supplementary material for: The Roles of Plasticity and Selection in Rapid Phenotypic Changes at the Pacific Oyster Invasion Front in Europe
Source: Mol Ecol. 2025 Feb 7;34(23):e17684. doi: 10.1111/mec.17684 (PMC12684338; doi:10.1111/mec.17684)
Supplement: Supplementary file 11 — Data S11. Local PCA across the genomes of the parents from the current invasion front, for each scaffold. Window size of 10,000 SNPs. The first five dimensions of an MDS on the eigenvector loadings are shown. [file MEC-34-e17684-s008.pdf]

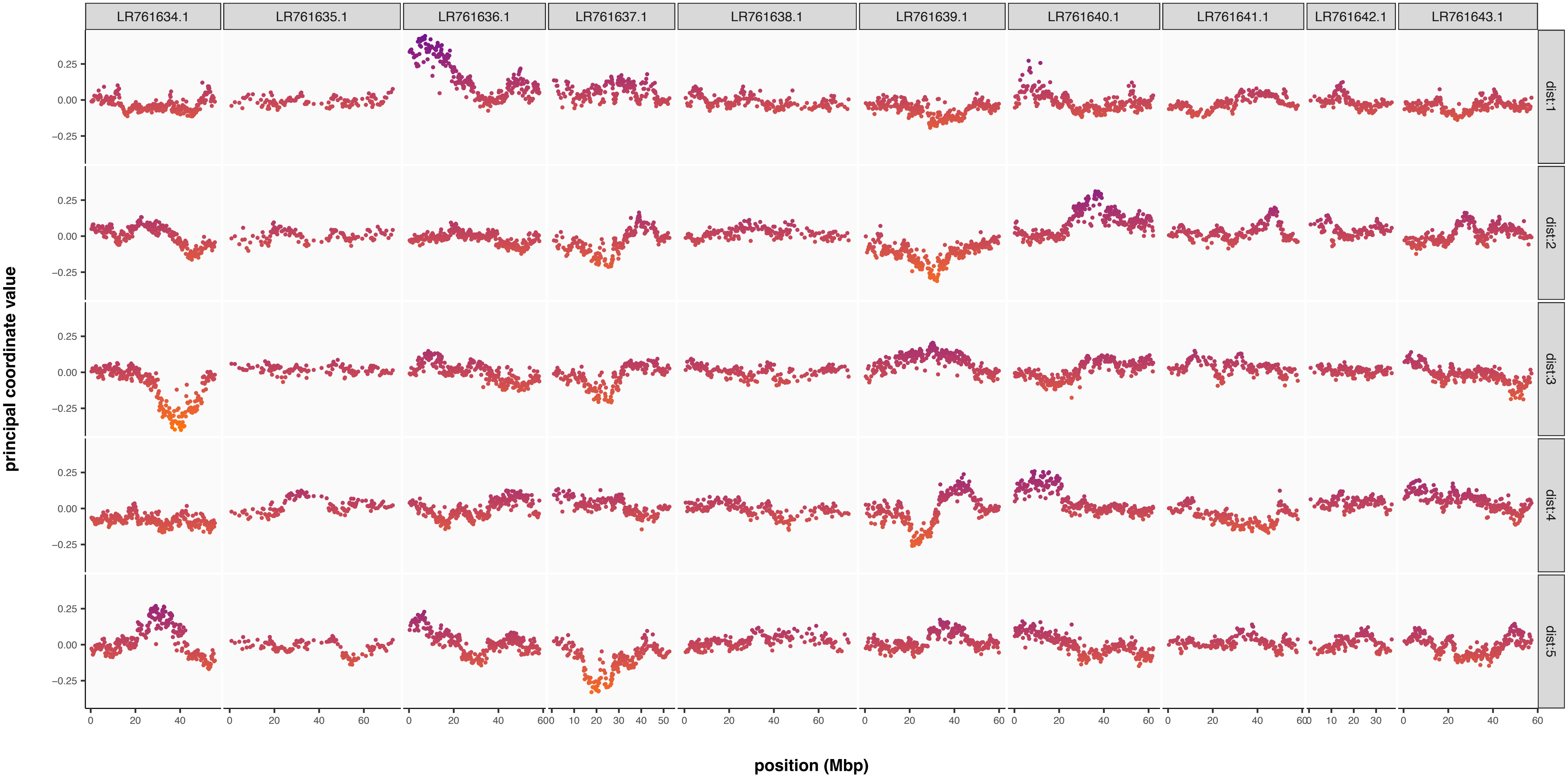

Supplementary Data S10. Local PCA across the genomes of the parents from the current invasion front, for each scaffold. Window size of 10,000 SNPs. The first five dimensions of an MDS on the eigenvector loadings are shown.
